# Inferring Growth Control Mechanisms in Growing Multi-cellular Spheroids of NSCLC Cells from Spatial-Temporal Image Data

**DOI:** 10.1371/journal.pcbi.1004412

**Published:** 2016-02-11

**Authors:** Nick Jagiella, Benedikt Müller, Margareta Müller, Irene E. Vignon-Clementel, Dirk Drasdo

**Affiliations:** 1 Institute for Computational Biology, Helmholtz Zentrum München, Neuherberg, Germany; 2 INRIA Paris, Centre de recherche Inria de Paris, Paris, France; 3 Interdisciplinary Centre for Bioinformatics, Leipzig University, Leipzig, Germany; 4 Institute for Pathology Heidelberg (iPH), Heidelberg University Hospital, Heidelberg, Germany; 5 Faculty of Medical and Life Sciences, Furtwangen University, Furtwangen, Germany; 6 Laboratoire Jacques Louis Lions, Sorbonne Universités UPMC Univ. Paris 6, Paris, France; University of Oxford, UNITED KINGDOM

## Abstract

We develop a quantitative single cell-based mathematical model for multi-cellular tumor spheroids (MCTS) of SK-MES-1 cells, a non-small cell lung cancer (NSCLC) cell line, growing under various nutrient conditions: we confront the simulations performed with this model with data on the growth kinetics and spatial labeling patterns for cell proliferation, extracellular matrix (ECM), cell distribution and cell death. We start with a simple model capturing part of the experimental observations. We then show, by performing a sensitivity analysis at each development stage of the model that its complexity needs to be stepwise increased to account for further experimental growth conditions. We thus ultimately arrive at a model that mimics the MCTS growth under multiple conditions to a great extent. Interestingly, the final model, is a minimal model capable of explaining all data simultaneously in the sense, that the number of mechanisms it contains is sufficient to explain the data and missing out any of its mechanisms did not permit fit between all data and the model within physiological parameter ranges. Nevertheless, compared to earlier models it is quite complex i.e., it includes a wide range of mechanisms discussed in biological literature. In this model, the cells lacking oxygen switch from aerobe to anaerobe glycolysis and produce lactate. Too high concentrations of lactate or too low concentrations of ATP promote cell death. Only if the extracellular matrix density overcomes a certain threshold, cells are able to enter the cell cycle. Dying cells produce a diffusive growth inhibitor. Missing out the spatial information would not permit to infer the mechanisms at work. Our findings suggest that this iterative data integration together with intermediate model sensitivity analysis at each model development stage, provide a promising strategy to infer predictive yet minimal (in the above sense) quantitative models of tumor growth, as prospectively of other tissue organization processes. Importantly, calibrating the model with two nutriment-rich growth conditions, the outcome for two nutriment-poor growth conditions could be predicted. As the final model is however quite complex, incorporating many mechanisms, space, time, and stochastic processes, parameter identification is a challenge. This calls for more efficient strategies of imaging and image analysis, as well as of parameter identification in stochastic agent-based simulations.

## Author Summary

We here present how to parameterize a mathematical agent-based model of growing MCTS almost completely from experimental data. MCTS show a similar establishment of pathophysiological gradients and concentric arrangement of heterogeneous cell populations as found in avascular tumor nodules. We build a process chain of imaging, image processing and analysis, and mathematical modeling. In this model, each individual cell is represented by an agent populating one site of a three dimensional un-structured lattice. The spatio-temporal multi-cellular behavior, including migration, growth, division, death of each cell, is considered by a stochastic process, simulated numerically by the Gillespie algorithm. Processes on the molecular scale are described by deterministic partial differential equations for molecular concentrations, coupled to intracellular and cellular decision processes. The parameters of the multi-scale model are inferred from comparisons to the growth kinetics and from image analysis of spheroid cryosections stained for cell death, proliferation and collagen IV. Our final model assumes ATP to be the critical resource that cells try to keep constant over a wide range of oxygen and glucose medium concentrations, by switching between aerobic and anaerobic metabolism. Besides ATP, lactate is shown to be a possible explanation for the control of the necrotic core size. Direct confrontation of the model simulation results with image data on the spatial profiles of cell proliferation, ECM distribution and cell death, indicates that in addition, the effects of ECM and waste factors have to be added to explain the data. Hence the model is a tool to identify likely mechanisms at work that may subsequently be studied experimentally, proposing a model-guided experimental strategy.

## Introduction

**Fig 1:**
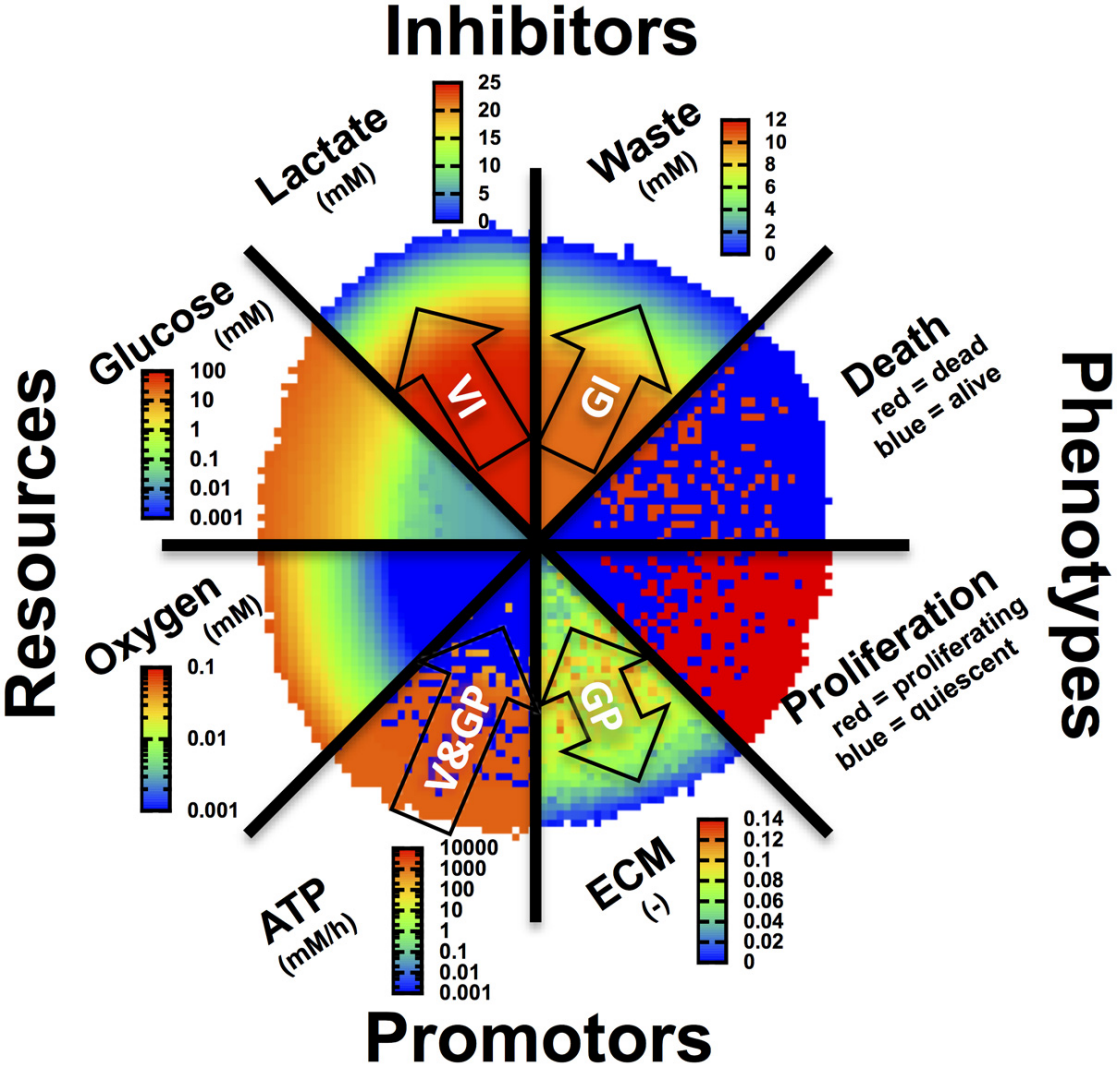
Radial organization of spheroids. Combination of images of a cross-section of a tumor simulated with the model presented in this article depicting the spatial organization of cellular phenotypes (proliferating, dying) and the molecular agents considered by the proposed model as main resources (oxygen, glucose), growth/viability promotors (GP/VP) or growth/viability inhibitors (GI/VI). The arrows point into the direction from high to low concentrations. The image shall be compared with the corresponding scheme in ref. [8], which shows a combination of images of spheroid median sections studied with different technologies: autoradiography, TUNEL assay, bioluminescence imaging, and probing with oxygen micro-electrodes.

In early development, tumors grow up to 1–2mm in diameter, nourished by the nutrients and oxygen provided by the existing vasculature. Either 2D or 3D cell culture systems are utilized as biological models to study that phase, or aspects usually occurring in later phases of tumor growth and development. Current 2D cell culture approaches are only of limited use to investigate tumor progression in these stages, as they neglect crucial histo-morphological and functional features of these avascular micro-metastases or inter-capillary micro-regions of solid in vivo tumors. During the last decades, great effort has been undertaken to generate biological 3D models that describe the early phases of tumor development in a tissue context more accurately. They can thus serve as intermediate systems between traditional 2D cell culture and complex in vivo models ([3, 4]). Of these approaches, Multicellular Tumor Spheroids (MCTS) offer easy handling and fast generation, even for larger batches, and automation ([5, 6]). MCTS as a model system can be well characterized and have been shown to reproduce the spatial organization and micro-environmental factors of in vivo micro tumors, such as relevant gradients of nutrients and other molecular agents and deposition of Extracellular Matrix (ECM) (see Fig 1) ([7, 8]). Furthermore, gene expression studies revealed substantial differences in both the baseline profiles and profiles after stimulation between 2D and MCTS cultures. The latter is decisively closer to patients profile gene expression ([9–11]). Consequently, MCTS have now been established as experimental systems for both basic research and high throughput screening of clinically relevant drugs (reviewed by: [12, 13]). As already explained in [14] the organization of the different cell phenotypes within a MCTS (growing, quiescent and dead) is supposed to be radial, and to be controlled by different factors: growth promoters (GP), viability promoters (VP), growth inhibitors (GI) and viability inhibitors (VI). In the case of spheroids the promoters are mainly delivered from the growth medium surrounding the tumor (with exception of ECM) while inhibitors are generally assumed to be produced by the tumor itself. As a consequence, the local composition and interplay of those factors favor different transitions between cell phenotypes at different distances from the tumor border.

To understand the dynamics of avascular tumor growth, several mathematical models were proposed linking the growth kinetics on the multi-cellular level (radius/volume in time) with mechanisms on cell or subcellular scales (cell growth, contact-inhibition, nutrient limitation etc.). They can be classified in two main approaches: (1) continuum models of the different components densities that evolve in time and space following PDEs (see e.g. [15–18]), (2) agent-based models that describe each cell individually and how it grows, divides, moves and dies (see e.g. [19–21]). When cells are modeled as agents, oxygen, nutrients and/or growth factors or inhibitors are often modeled by continuum models [1, 2, 22–31] or with simplified assumed profiles [32, 33]. Hybrid models on the other hand combine within the same framework the two model types for the cells, depending on the tumor zone (e.g. [26]).

Despite the large variety of models, identification of a plausible mechanistic model and its quantitative parameterization able to quantitatively explain a large set of data and predict the outcome of experiments that were not used to calibrate the model remains a difficult task. Issues are the large number of parameters and the lack of validation of the underlying mechanisms. Different models have successfully been fitted to the growth dynamics of cell populations (e.g. [2, 22] consider only the population size but not the diameter, [1, 34] consider both) relying on different mechanisms but leading to the same growth curves. For example the transition from exponential to linear radius growth phase can be due to contact-inhibition or nutrient-limitation. Based on the growth curves alone, model selection could not be made, indicating that development of a mathematical model only relying on growth curves is insufficient. In this paper we pursue a quantitative image-based approach based on bright field micrographs. This is in-line with a recent trend in large-scale simulations of brain tumors based on magnetic resonance imaging (MRI) [35, 36] following inspiring work by Swanson and co-workers [37]. Histological information has been used recently by Frieboes et. al. [38], who included histological staining measurements in a partial-differential-equation tissue model of Non-Hodgkin lymphoma growth, and by Macklin et. al. [39–41] who developed a multiscale model, mimicking cells as individual agents subject to forces, to predict ductal carcinoma growth in individual patients, including histological information.

In this paper we study mathematical model development and model parameterization by comparison with experimental data for different oxygen and glucose concentrations in the medium for the non-small cell lung cancer (NSCLC) cell line SK-MES-1. These data consist in the growth kinetics and the corresponding spatial staining patterns for nuclei, different cell states and cell environment, namely, HOECHST for cell nuclei, KI67 for proliferation, TUNEL for dead cells, collagen IV for ECM. We study in how far mechanisms that have not been directly assessed can be inferred by simultaneous matching of simulation results with experimental results on many experimental observables. Our strategy is stepwise: we first develop a model for one growth condition only, and then expand the model to capture additional growth conditions after verifying that the previous (simpler) model stage was incapable of explaining the added growth data. For this, we perform many computer simulations with the “previous” model varying each model parameter within its physiological range. Finally we arrive at a model that can almost completely be projected to the experimentally derived scheme on spheroid growth (compare Fig 1 to reference [8]). By such a stepwise strategy involving experiments, imaging, image analysis and modeling, an order mechanism during liver regeneration could be identified [42], indicating that such a strategy may be powerful in unveiling interplaying mechanisms in multi-cellular organization.

## Results

### Experiments and Image Processing

**Fig 2:**
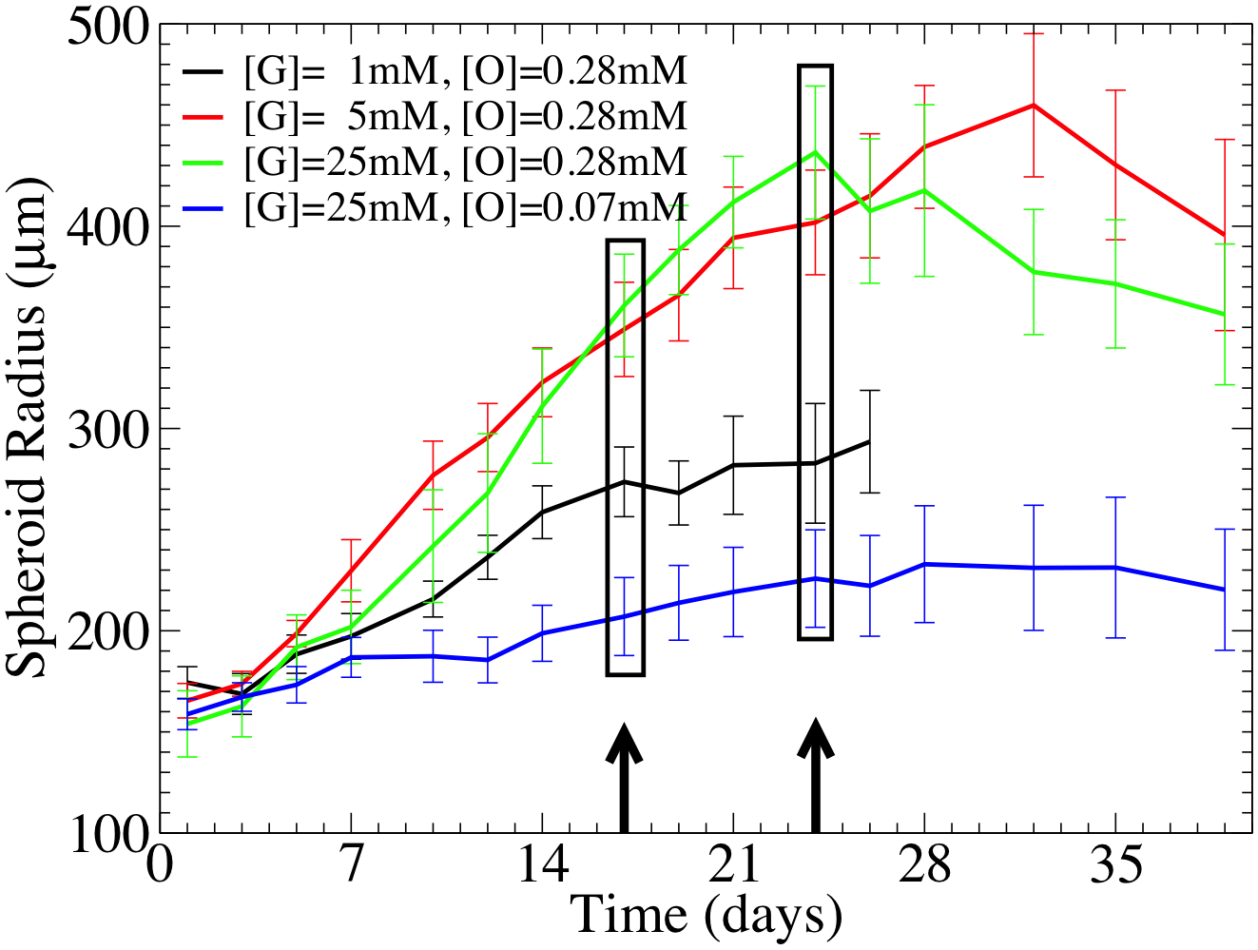
Growth curves of MCTS cultivated under different nutrient conditions. The arrows and boxes indicate the time points where histological images were taken.

**Fig 3:**
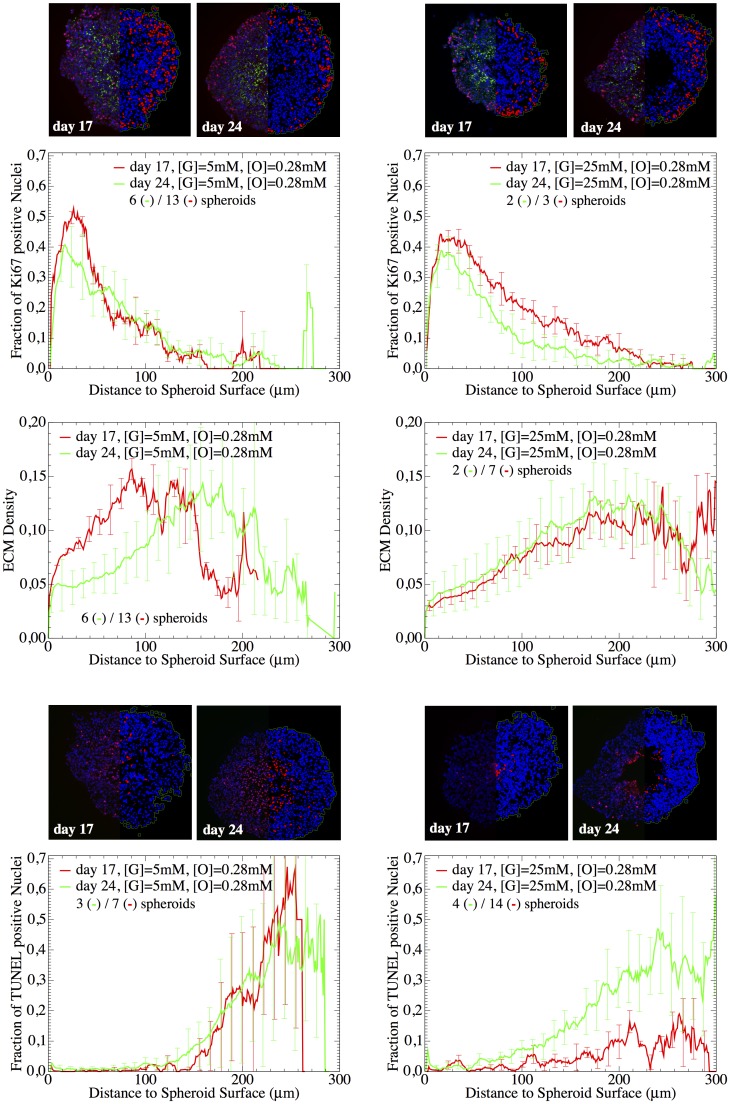
Quantification of proliferating (top) and dying (bottom) cell nuclei as well as of extra-cellular matrix density (center). The images (top) depict cross-sections taken at different time points (T3 = 17d, T4 = 24d) of spheroids grown under different nutrient conditions ([G], [O_2_]). The colors indicate cell nuclei (blue), extra-cellular matrix (EMC) (green) and proliferation (red, left) or cell death (red, right). The curves (bottom) represent the radial profiles of the fraction of proliferating and dying cells, and the ECM density (intensity of ColIagen IV staining), inferred from averages over images from several spheroids growing under the same conditions. Bars indicate the standard deviation. With ECM density we denote the fluorescence value of Collagen IV, which varied in the interval [0, 1].

In order to study the influence of environmental conditions on the growth dynamics of tumor spheroids (Fig 2), SK-MES-1 cells were cultivated in-vitro as multi-cellular tumor spheroids under different nutriment conditions, with the hanging drop method. Then, at different points in time, spheroid size was determined with bright field microscopy and some of the spheroids were frozen, cryosectioned, stained and imaged with fluorescence microscopy (Fig 3). For a detailed description see the Materials and Methods section.

#### Growth curves

Fig 2 shows the temporal growth curves for the spheroid radius for four different culture conditions (see Table 1). The figure suggests the existence of different growth phases. The growth phase in the first two days cannot clearly be characterized but concluding from earlier work ([1, 43]) it may emerge from a transition of an exponential to a sub-exponential growth phase. Between days 2–21, growth is over all approximately linear, followed by saturation or even shrinkage of the spheroid. The crossover points between the growth phases depend on the oxygen and glucose medium concentrations. Hence there must be an inflection point between the initial convex and the final concave (saturation) phase where the 2nd derivative of the radius with respect to time changes from positive to negative values. For [G] = 5mM, 25mM, [O] = 0.28mM, the growth behavior is very similar. Lowering glucose to [G] = 1mM or oxygen to [O] = 0.07mM leads to significantly slower growth. At about 24 days the radius is saturating or even shrinking.

**Table 1:** Nutrient conditions.

**Condition**	**[G]**	**[O_2_]**	**Note**
I	1mM	0.28mM	hyponourished
II	5mM	0.28mM	physiological
III	25mM	0.28mM	hypernourished
IV	25mM	0.07mM	hypoxic

#### Radial profiles of histological quantification

**Fig 4:**
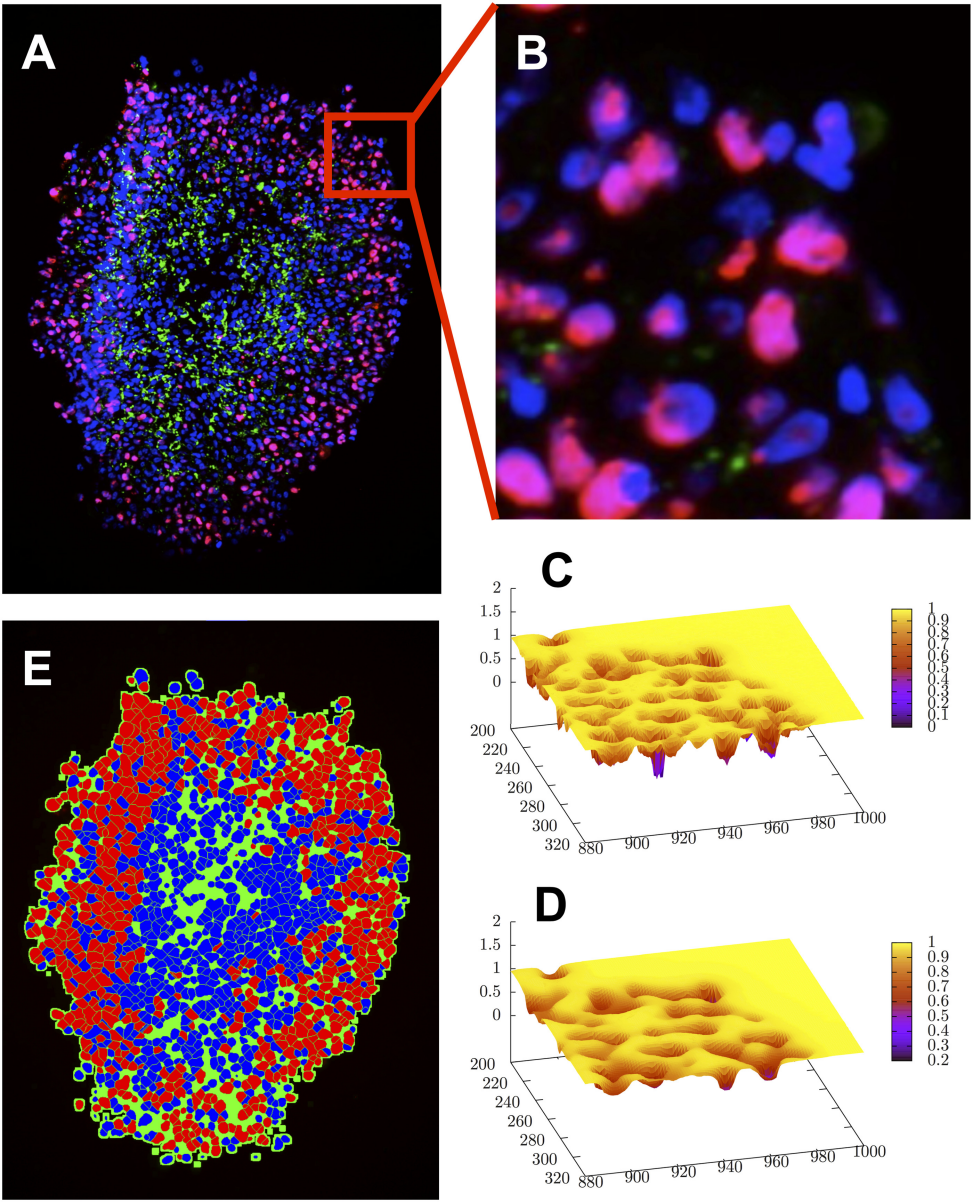
Image smoothing and segmentation. (A) The micrograph shows a cryosection of a spheroid stained with HOECHST (red), Ki67 (blue) and Collagen IV (green). The original image was smoothed with a median filter. For a zoomed-in section of the image (B), the original (C) and smoothed version (D) of the image are visualized as landscapes of the inverted blue color channel intensities, 1 − *I*^*blue*^. (E) The cell nuclei were segmented from the blue color channel and differentiated between Ki67 positive (red) and negative (blue) nuclei by means of the red channel. The spheroid lumen (green) is approximated by inflated nuclei.

In order to obtain spatial information on the distribution of cells, proliferating cells, dead cells and extracellular matrix (ECM), several multi-cellular spheroids were stained and imaged. Fig 3 shows typical images stained with HOECHEST, Ki67, TUNEL and Collagen IV for cell nuclei, proliferating cells, dying cells and extra-cellular matrix (ECM), respectively for [G] = 5mM and [G] = 25mM. The images were segmented and cells classified into being proliferating (if the nucleus was Ki67—positive), dying (if TUNEL—positive) or quiescent (if neither Ki67 nor TUNEL positive) (Fig 4, details see in Materials and Methods). From the segmented images the spatial profiles for proliferating, quiescent and dead cells were investigated, from the border to the interior (for details see section Quantitative Image Analysis). Starting the investigation of spatial profiles from the border (as opposed to from the center of mass) ensured that the profiles at the border were correctly captured even if the MCTS had an ellipsoidal instead of a spherical shape, which was often the case (see for example, Fig 4A and 4E). The disadvantage of this choice was that the number of events (Ki67 positive nuclei, ECM density etc.) inside the tumor was usually very rare so that the data points with distance of more than 200 micro-meters from the border have to be considered with great caution (far inside the tumor the number of events is very small and the fraction depends on the bin-size, see Fig S13 in S1 Document). The fraction of proliferating cells decreases the farther the proliferating cells are inside the spheroid (Fig 3). The proliferation profiles do (almost) not change over time and are very similar for [G] = 5mM and [G] = 25mM in the growth medium. Cell death increases from the spheroid border to its interior. There is a marked difference in the cell death profile between both nutrient conditions in that for 5mM the necrotic core emerges earlier (already at day 17, while for 25mM not until day 24). Interestingly after 24 days, cell death seems to occur at smaller penetration depth for [G] = 25mM than for [G] = 5mM. The TUNEL assay does not permit distinguishing between necrotic and apoptotic cells ([44]). For this reason we also did some pilot experiments selectively staining samples with Caspase 3. In these samples we could see activation of Caspase 3 in the peripheral parts of what we would identify as the “necrotic core”, although this may also not yield a definite distinction between apoptosis and necrosis, as Caspase 3 activation has been shown to be involved in both ([45, 46]). Thus we could not discriminate between different forms of cell deaths in the experiments within this paper. Indeed, as this cell culture system does not include macrophages, removal of apoptotic cell bodies is very slow so that distinguishing between necrotic and apoptotic cells seemed dispensable. This assumption was self-consistently confirmed as very small apoptosis and lysis rates had to be assumed in the model to explain the experimental findings so that apoptosis did not have to be taken explicitly into account.

The amount of extra-cellular matrix (ECM) increases with the penetration depth until 100–200*μm*. For larger penetration depths it seems to decrease again, but the data is too noisy to infer a clear course. Counterintuitively, cells at the spheroid surface have a significantly lower proliferative activity than those a few layers inside.

### Mathematical Modeling: Main Components Needed

Our objective is to explain the experimentally observed growth pattern for different glucose and oxygen medium concentrations within one mathematical model. For this purpose we first searched for a minimal model (in the sense specified in the abstract) explaining the experimental tumor growth observations for medium concentrations of [G] = 25mM and [O] = 0.28mM, and then stepwise extended this model to capture the other growth conditions (for illustration of the stepwise model development strategy, see Fig S3 in S1 Document). We based our choice of possible control mechanisms upon prior knowledge guided by published information and own experiments. We have chosen the condition of maximum glucose and oxygen medium concentration as the similarity of the growth kinetics for [G] = 25mM and [O] = 28mM versus [G] = 5mM and [O] = 28mM suggests that for the former condition neither glucose nor oxygen may be limiting (see also Discussion below). This line of argument is supported by findings of Freyer and Sutherland for another cell type at almost the same oxygen and glucose medium conditions (compare with [1]). We fit at each model development step all parameters again. So the fits shown in this article were the best we could obtain for the respective model. However, due to the large search space and duration of simulation of at least one day (reference computer: Intel(R) Xeon(R) processor X5680 3.33GHz 12M cache 6-core and 144 GB DDR3-RAM 1333 MHz) it cannot completely be excluded that further parameter searches could give additional slight improvements. In order to promote readability, we enumerate the model at each development level.

Usually we performed for each parameter set only a single simulation i.e., a single realization of the stochastic growth process. This can be justified by observing that the growth process, that starts with about 10000 cells as in the experiment, is self-averaging such that the variations for different realizations of the growth process for the same parameters are negligible (Fig S11 in S1 Document).

Our basic model considers each cell individually within an agent-based model i.e. each individual cell is represented by an agent. Molecules, which finally are glucose, oxygen and lactate, as well as extra-cellular matrix and “waste” material released by dying cells, are represented by their local concentrations. We use the term “molecules” in what follows to generically describe these environmental factors that affect the cells. The model is three-dimensional. In the following sections we will introduce briefly the main model components. A detailed description of the model as well as the biological processes mimicked can be found in the material and method section and in the supporting information (S1 Document).

#### Cells

**Fig 5:**
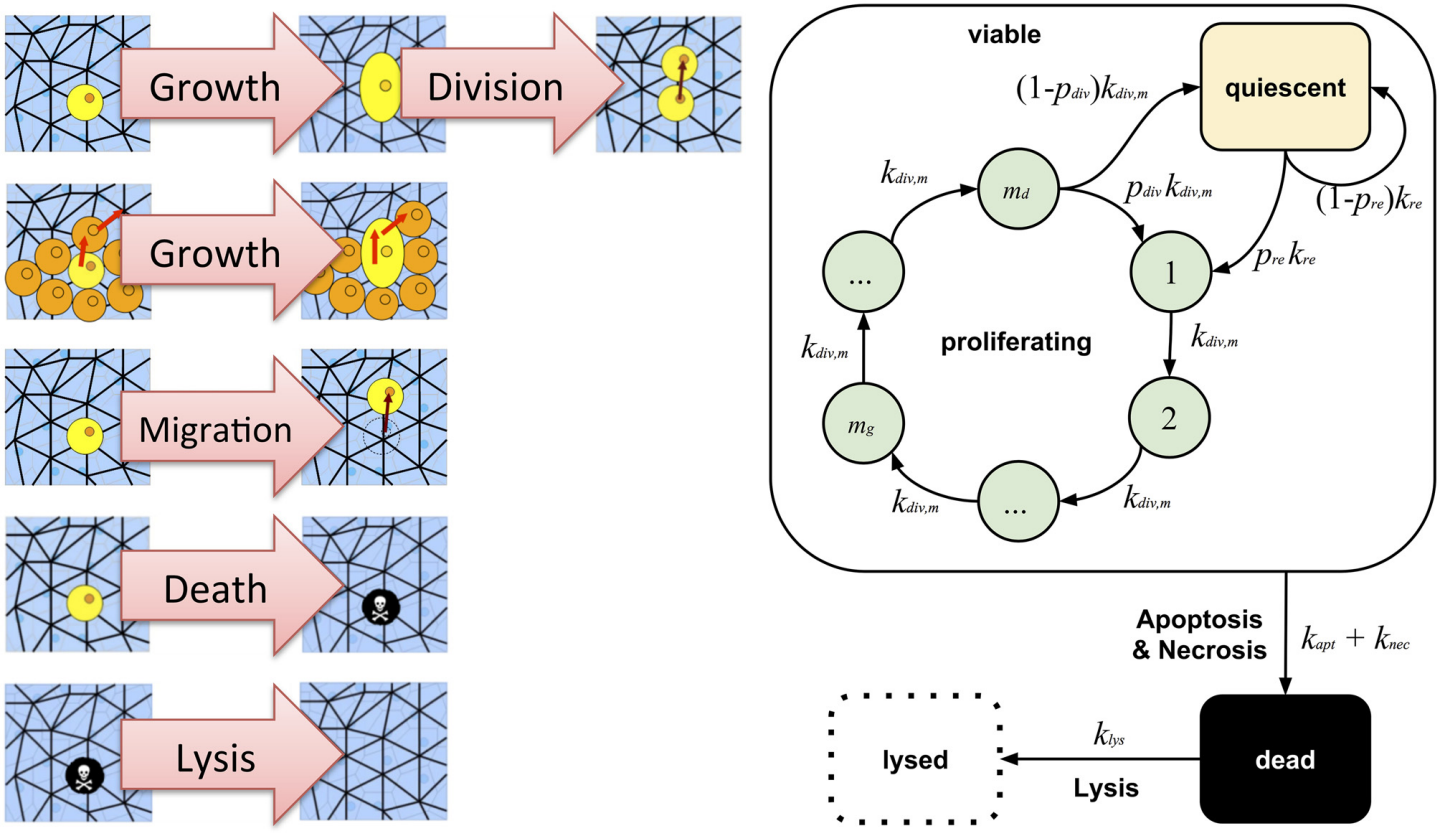
Scheme of cell states and the biological processes a cell can undergo: cell cycle (including cell growth and division), migration, cell death (apoptosis and necrosis) and lysis. Left panel: A cycling cell grows by occupying two neighboring lattice sites after a fraction of transitions in the cell cycle. Growing cells can push a certain number of cells aside. Cells can migrate by hopping from one lattice site to its neighbor lattice site. A cell dies with rate *k*_*neck*_ and is consequently lysed with rate *k*_*lys*_. (The schemes in the left panel are 2D for clarity; the simulations however were all in 3D). Right: All processes are modeled as Poisson processes. A cell in the cell cycle undergoes *m*_*d*_ transitions until splitting into two daughter cells. Composed of *m*_*d*_ sub-processes the cell cycle time ends up following an Erlang distribution. Increasing *m*_*d*_ will lead to sharper cell cycle distributions. A cell transitions from one cell cycle state (CCS) to the next with rate *k*_*div*,*m*_. If a cell is in state m_g_ it will grow. If it is in state *m*_*d*_ it will divide into 2 daughter cells during the next transition. Furthermore, the 2 daughter cells will either enter the first CCS proportional to probability *p*_*div*_ or become quiescent (G0) proportional to probability 1 − *p*_*div*_. A cell in quiescence can reenter the cell cycle with rate *k*_*re*_ and probability *p*_*re*_. Table 2 indicates how all transition rates and probabilities are calculated for models 1–4.

The cells were modeled as individual objects populating the sites of an unstructured lattice (see supplement, [25, 47]) with no more than one cell on a lattice site. The cells could be in either of three states: proliferating, quiescent or dying. Depending on their state and their local environment they were able to progress in or reenter (from G0) into the cell cycle, grow, migrate, die or undergo lysis (Fig 5). Each of these processes required a set of necessary conditions, and was executed with a certain rate. Both, processes and rates depended on the cellular neighborhood and the local molecular concentrations (see below). The temporal dynamics of the cells was described by a “master equation” mimicking the time evolution of the probability of a certain multicellular configuration. This equation was numerically simulated with the Gillespie algorithm (see S1 Document).

#### Molecules

For each molecule type a continuum representation of its concentration was chosen. Its spatial-temporal dynamics was modeled by a partial differential equation (PDE). For each molecule the equation could be casted into the form of a reaction diffusion equation

$$\begin{array}{c} \partial_{t}u=\nabla \cdot \left(D_{u}\left(\sigma \right)\nabla u\right)+r_{u}\left(\sigma ,u\right), \end{array}$$ (1)

where *t* is the time, *u* ∈ {*G*, *O*, *L*, *ECM*, *W*} is the concentration of either glucose, oxygen, lactate, extra-cellular matrix or waste, *D*_*u*_ is the respective diffusion coefficient, and *r*_*u*_ a rate function representing the reactions involving *u*(= *u*(**r**, *t*)). **r** denotes the position in space. The diffusion coefficients as well as the reaction functions depended on the local cell density *σ*(**r**, *t*) = ∑_*k*_ *δ*(**r** − **r**_*k*_), **r**_*k*_(*t*) being the position of cell *k* at time *t*. The reactions were specific to each component (molecules or ECM): cells consumed glucose and oxygen with −*r*_*G*_ and −*r*_*O*_, lactate was produced by under-oxygenated cells with *r*_*L*_, ECM was produced by cells and disintegrated with a certain rate, and waste was released by dying cells.

### Mathematical Modeling: Coupling of Scales and Comparison with Data

A number of parameters related uniquely to cells or molecules were either taken or estimated from literature (e.g. molecular diffusion coefficients, consumption rates, cell cycle time distribution). Others could be inferred from the data presented in this article either directly (e.g. initial conditions, cell size) or by sensitivity analysis (e.g. cellular division, cell death and lysis rates) (see Table S1 in S1 Document). The identification of the main mechanisms coupling the cellular and molecular kinetics and their parameterization (see Fig 5) was subject to the model comparison with data explained below.

The labeling patterns in Figs 3 and 4 confirm a border distance-dependent “zonation” as discussed in the introduction (compare also [8] and Fig 1). In the following we will study the influence of cell-cell-contacts, extra-cellular matrix and metabolic compounds (nutrients/metabolites) to infer the corresponding model mechanisms. For the latter (metabolism) we will compare four different hypotheses (model 1–4). A summary of the equations used to calculate the transition rates and probabilities depicted in Fig 5 for all models studied in the following sections can be found in Table 2.

**Table 2:** Transition rates and probabilities of models 1–4. All models have *p*_*re*_ *k*_*re*_ = 0, *k*_*apt*_ = *const*. and *k*_*lys*_ = *const*. in common. The transition rates *k*_*div*,*m*_ between individual cell cycle steps is given by *k*_*div*,*m*_ = *m*_*d*_ *k*_*div*_. All parameter values can be found in Table S1 in S1 Document.

**Model**	***p*_*div*_** =	***k*_*div*_** =	***k*_*nec*_** =
1	*e*^−*L*/Δ*L*^	$k_{div}^{max}$	$k_{nec}^{max}$
	⋅*H*([*ECM*] − [*ECM*]^*min*^)	⋅*H*([*G*][*O*] − *p*^*oxyglc*^)	⋅*H*(*p*^*oxyglc*^−[*G*][*O*])
2	*e*^−*L*/Δ*L*^	$k_{div}^{max}$	$k_{nec}^{max}$
	⋅*H*([*ECM*] − [*ECM*]^*min*^)	$\cdot H(p_{ATP}-p_{ATP}^{min})$	$\cdot H(p_{ATP}^{min}-p_{ATP})$
3	*e*^−*L*/Δ*L*^	$k_{div}^{max}$	$k_{nec}^{max}$
	⋅*H*([*ECM*] − [*ECM*]^*min*^)	$\cdot H(p_{ATP}-p_{ATP}^{min})$	$\cdot H(p_{ATP}^{min}-p_{ATP})$
			$\cdot \frac{{\left[L\right]}^{n}}{{\left({\left[L\right]}^{max}\right)}^{n}-{\left[L\right]}^{n}}$
4	*e*^−*L*/Δ*L*^	$k_{div}^{max}$	$k_{nec}^{max}$
	⋅*H*([*ECM*] − [*ECM*]^*min*^)	$\cdot H(p_{ATP}-p_{ATP}^{min})$	$\cdot H(p_{ATP}^{min}-p_{ATP})$
	$\cdot H(n_{exp}^{max}-n_{exp})$	⋅*H*(1 − 0.5([*W*] − [*W*]^*max*^))	$\cdot \frac{{\left[L\right]}^{n}}{{\left({\left[L\right]}^{max}\right)}^{n}-{\left[L\right]}^{n}}$
		⋅*H*(1 − 0.5([*O*]^*min*^ − [*O*]))	

#### Mechanical growth-inhibition: Probabilistic cell decision

The growth curves and proliferation profiles for [G] = 25mM and [G] = 5mM look quite similar while the necrotic core sizes at 17 days look very different for [G] = 25mM and [G] = 5mM (Figs 2 and 3). An equivalent observation has been made for data on EMT6/Ro cells [14] and could be shown by model simulations to strongly indicate that proliferation is not controled by non-glucose-related factors but by a mechanical form of contact inhibition [1]. Otherwise, the proliferating rim and the size of the necrotic zone should both be affected. Model simulations with our lattice model confirmed this finding.

In previous articles a biomechanical form of contact-inhibition was assumed to be a deterministic decision depending on the local pressure or deformation in growing cell populations ([1, 2, 48–50]). Nevertheless, as can be seen from model studies of growing multi-cellular spheroids in an off-lattice model in [24] and [34], a deterministic criterion based on a deformation threshold is able to yield a gradual change of the fraction of dividing cells as a consequence of local stochastic events such as cell micro-motility or randomness in the cell cycle duration,which both introduce stochastic fluctuations in deformation and mechanical stress. As demonstrated in refs. ([25, 43]) an extended proliferating rim can be qualitatively captured within a lattice model (as the one chosen in this article) by introducing a maximum distance, Δ*L*, over which a cell is able to push other cells away in order to grow. This probability that a cell enters the cell cycle can then be defined as follows

$$\begin{array}{c} p_{div}:=H\left(L-\Delta L\right), \end{array}$$ (2)

where *H* is the Heaviside function, *H*(*x*) = if *x* < 0, *H*(*x*) = 1 if *x* ≥ 0, and *L* is the distance to the closest free lattice site.

**Fig 6:**
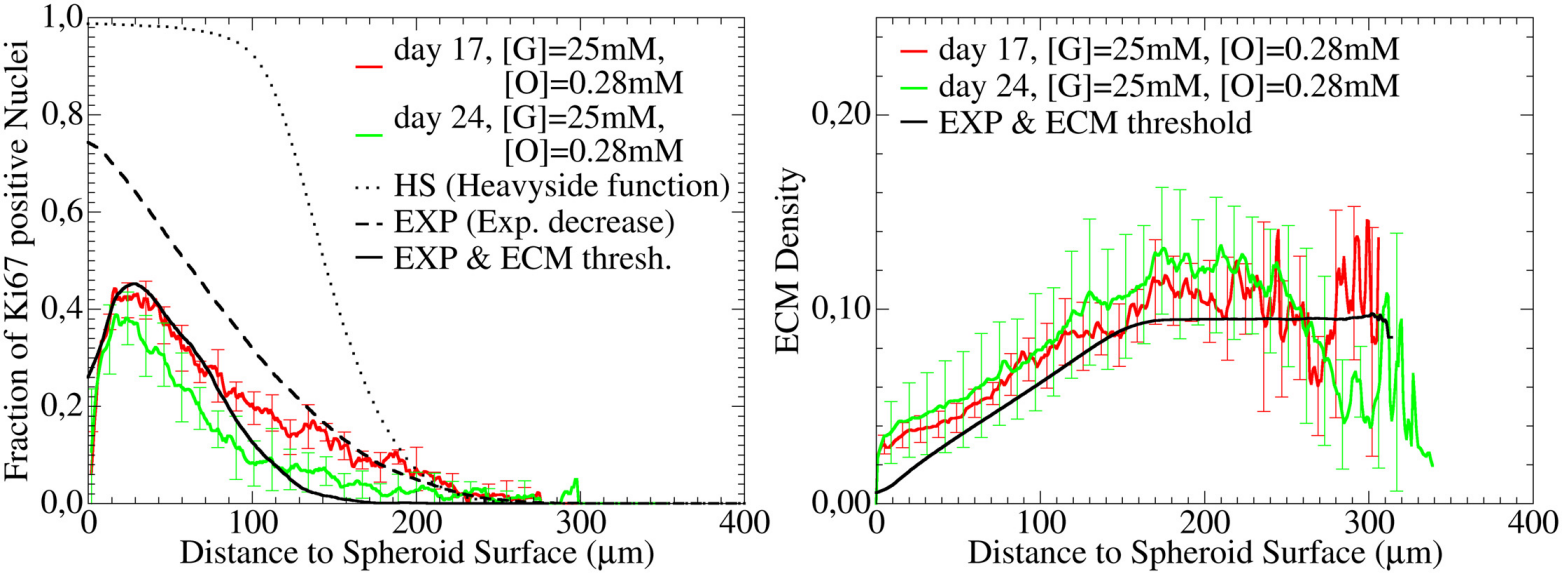
Experimental and model-predicted fraction of Ki67 positive cells (left) and Collagen IV (ECM) density (right) for [G] = 25mM, [O] = 0.28mM at day 17 (red) and day 24 (green) vs. distance from the spheroid surface. A deterministic cell cycle progression depending on the distance of a cell from the surface, generates sharp transitions that are experimentally not observed (left, dotted line), while a probabilistic transition generates a smooth monotonically decreasing function of Ki67 positive cells in the simulations (left, dashed). If in addition cells can progress in the cell cycle only if the local concentration of ECM overcomes a threshold value [*ECM*]^*min*^, the experimentally found Ki67 profile is recovered (left, full line). Right full line: ECM concentration in the computer simulation.

However, as illustrated in Fig 6 (left panel) the introduction of such a sharp cut-off leads to a relatively sharp transition between outer proliferating and inner quiescent rim while the experimental profiles display a wide transition zone with gradual change of the frequency of cells in the cell cycle.The smooth experimentally observed transition could be obtained within a lattice model by choosing a probabilistic approach instead, with cells entering the cell cycle with a probability exponentially decreasing with Δ*L* (Fig 6, left panel):

$$\begin{array}{c} p_{div}:=e^{-L/\Delta L}. \end{array}$$ (3)

Hence the decision of a cell entering the cell cycle is stochastic, meaning that neighbor cells with the same distance to the border may behave differently. This generates local inhomogeneous cell behavior. The cell cycle progression probability (3) generates a profile with the highest Ki67 positive cell fraction at the outermost border. This tendency is a robust phenomenon and is also found in off-lattice models of growing cell population where the cell cycle progression is controlled by a deformation or a pressure threshold ([48, 51]). Hence we conclude that biomechanical control of cell cycle progression by a deformation or pressure threshold alone cannot explain a drop of proliferation at the margin of the multicellular spheroid.

#### Extra-cellular matrix

The experimental data exhibit that border cells have a lower proliferating activity than interior cells (Fig 6, left panel) while at the same time collagen IV staining display a lower ECM concentration close to the spheroid border (Fig 6; right panel). This suggests that the known control function of ECM in the cell cycle progression [50] may be responsible for the lower fraction of proliferating cells at the spheroid border. This assumption is supported by the observation that ECM binding and connected integrin signaling is an important part of proliferative signaling ([52, 53]).

Accordingly we assume that a minimal density of extra-cellular matrix, is required for cells to enter the cell cycle by replacing Eq 3 by

$$\begin{array}{c} p_{div}:=e^{-L/\Delta L}\cdot H\left(\left[ECM\right]-{\left[ECM\right]}^{min}\right), \end{array}$$ (4)

where [*ECM*] reflects the local concentration of ECM, and [*ECM*]^*min*^ is a threshold value (the values do not have a dimension as we directly work with the fluorescence intensity defined in the interval [0, 1]). Extra-cellular matrix is secreted by each cell, and decreases with a certain rate. Choosing [*ECM*]^*min*^ = 0.003, the extracellular matrix concentration behaves as experimentally observed (Fig 6), and the simulated proliferation profile shows good agreement with the experimental profile.

Eq 4 determines the probability of cell cycle entrance of each daughter cell directly after division. For most of the paper we assumed that this decision is irreversible (*p*_*re*_ = 0 in Fig 5) but we also considered the consequence of exit from G0 (*p*_*re*_ > 0 in Fig 5). For the latter we tested different alternative rules (see Fig S4 and S5 in S1 Document) that may be subsumed by the equation for the exit rate, *r*(*L*) = *k*_*re*_ *p*_*re*_(*L*). This equation assumes that the cell probes with rate *k*_*re*_ exit of G0 occurring with probability *p*_*re*_(*L*). The probability that the entrance occurs within time interval [*t*, *t* + Δ*t*) at depth *L*(*t*) is then $p\left(\text{re}-\text{entrance in }\left[t,t+\Delta t\right)\right)=k_{re}p_{re}e^{-\int_{t}^{t+\Delta t}p_{re}k_{re}dt^{\prime }}\Delta t$. In case *p*_*re*_ has the functional form as *p*_*div*_ in Eq 4, the rate *k*_*re*_ must be very small in order to achieve agreement between data and model (Fig S4 in S1 Document). We found *k*_*re*_ = 0.001/2.4*h* (Fig S4 in S1 Document; given by Eq 4). I.e., only in 100/*p*_*re*_ days on the average a specific cell exits G0.

We furthermore tested the hypothesis that cells become quiescent with probability 1 − *p*_*div*_ (as in Eq 4) and can only exit quiescence if their neighbor site becomes free (Fig S5 in S1 Document) which may occur if a cell is able to sense free space. In this case we can also obtain a good fit to the growth data if the threshold ECM concentration at which re-entrance can occur, is elevated. We note, that very few cell cycle reentrances from G0 could be observed during simulations, and that the simulation results were not sensitive at all to changes in the reentrance rates (see S1 Document). We concluded that the presence of cell—cycle re-entrance by exit of the quiescent state is not a critical parameter for MCS growth of SK-MES-1 cells. However, in case of drug therapy, where a drug may kill the outermost cells, cell-cycle re-entrance is expected to modify growth and survival of the treated cell population, and would then become a critical parameter.

In conclusion the cell-cycle re-entrance must be a very rare (negligibly rare) event in order to explain the data which is why we throughout the following neglect cell cycle re-entrance (for more details, see S1 Document).

#### Model 1: Glucose- and oxygen-limited growth and survival

We assumed deprivation from glucose and oxygen to be responsible for cell death. Schaller and Meyer-Hermann [2] proposed that cell survival is possible only if the product of glucose and oxygen overcomes a threshold *p*^*oxgluc*^ while below this threshold cells undergo cell death:

$$\begin{array}{c} k_{div}=k_{div}^{max}H\left(\left[G\right]\left[O\right]-p^{oxgluc}\right), \end{array}$$ (5)

$$\begin{array}{c} k_{nec}=k_{nec}^{max}H\left(p^{oxgluc}-\left[G\right]\left[O\right]\right), \end{array}$$ (6)

where *H* is the Heaviside function, $k_{div}^{max}$ and $k_{nec}^{max}$ are the maximal and *k*_*div*_ and *k*_*div*_ are the actual division and cell death rates, respectively. As no experimental measurements of glucose and oxygen consumption rates for SK-MES-1 cells were accessible, we assumed the rates to obey the same functional relationship as the oxygen and glucose consumption rates measured for EMT6/Ro cells (see Materials and Methods), permitting only the parameters to be different for SK-MES-1 cells than for EMT6/Ro cells.

**Fig 7:**
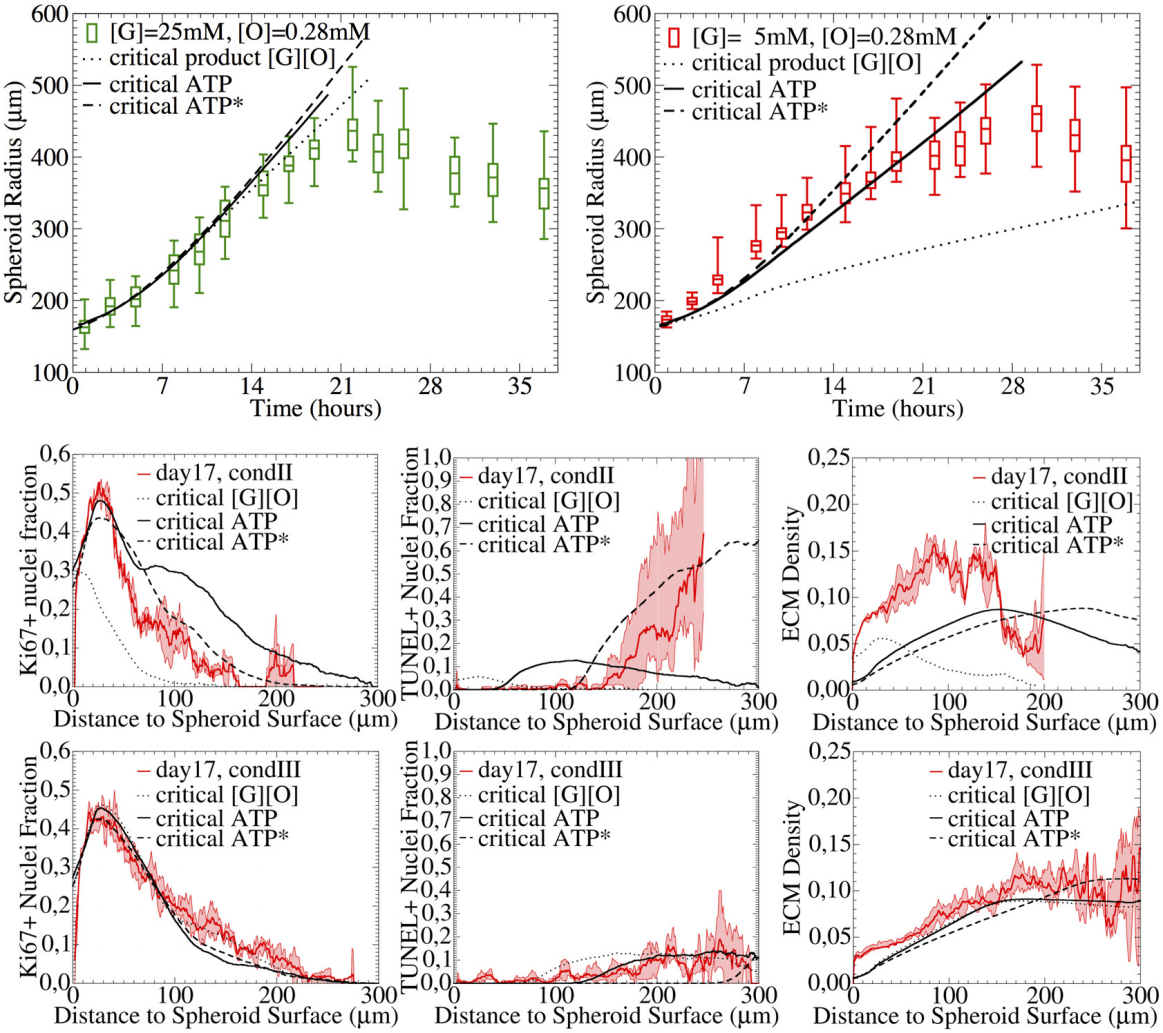
Growth kinetics (upper panel) and spatial profiles of TUNEL, Ki67, Collagen IV for [G] = 5mM, [O] = 0.28mM (condition II, upper right picture and middle panel) and [G] = 25mM, [O] = 0.28mM (condition III, upper left picture and low panel.). The dotted curves show simulations where cell cycle progression requires the product of local glucose and oxygen concentration to exceed a critical threshold, while below this threshold cells become quiescent and die. If the product is replaced by the local concentration of ATP, the agreement improves significantly (solid lines). For dashed curves the diffusion coefficient in medium was assumed to be *D*_*med*_ = 30*D*_*tum*_. The experimental growth curves are shown as boxplots (box: mean, lower & upper quartiles, horizontal dashes: minimum & maximum), and the radial profiles as composition of mean (bold line) and standard deviation (thin line).

Fig 7 shows that for this assumption (dashed lines) and nutrient medium condition III ([G] = 25mM, [O] = 0.28mM) the simulation results are in good agreement with the experimental growth curves and the radial profiles for ECM, proliferation and cell death, but completely fail for condition II ([G] = 5mM, [O] = 0.28mM). The size of the necrotic core and the growth-limitation are over-estimated.

#### Model 2: ATP-limited growth and survival

**Fig 8:**
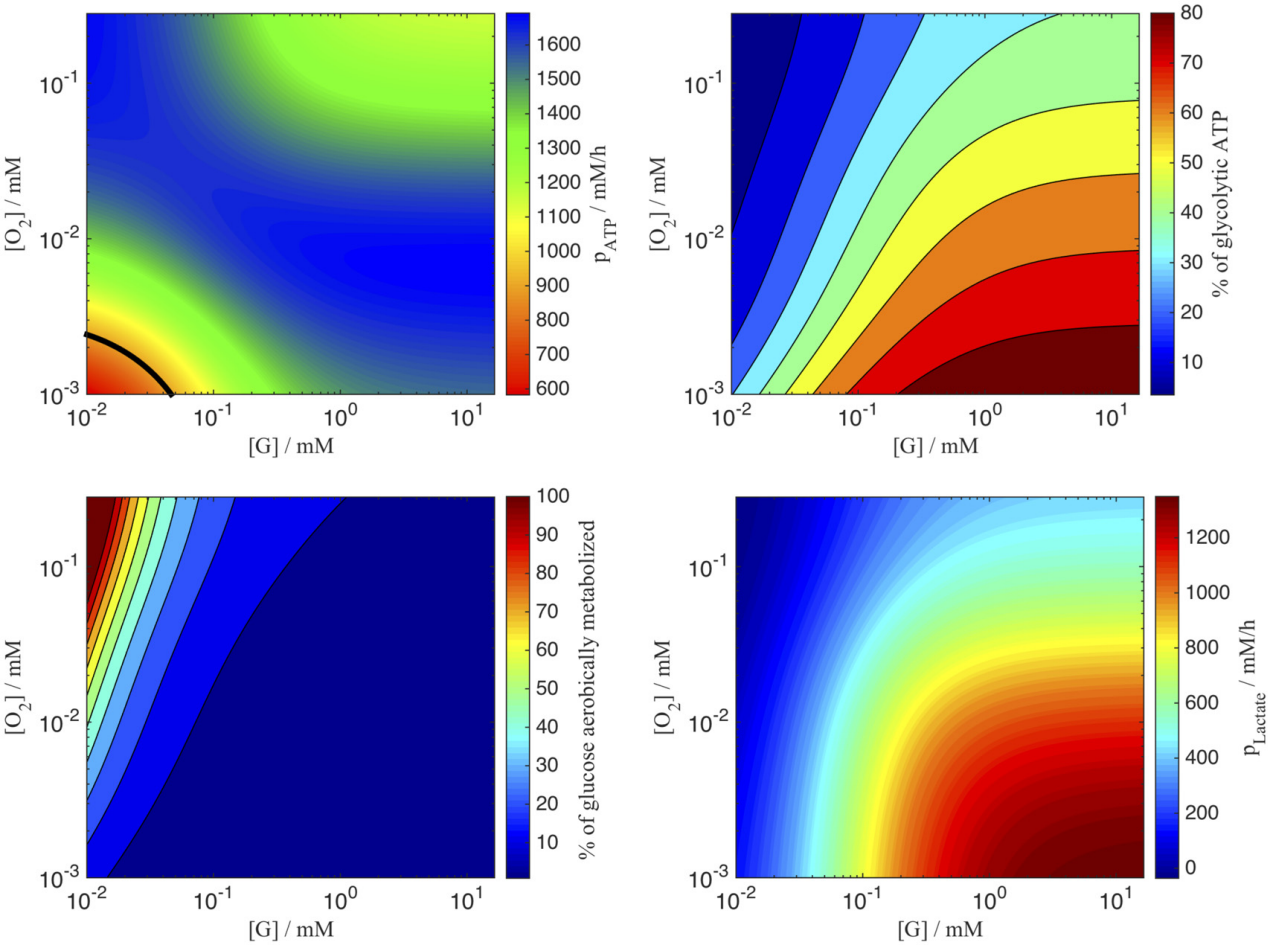
Nutrient-dependent metabolism. Production rates of ATP (top left) and lactate (top right). The threshold value of the minimal ATP requirement is depicted by the black line. Percentage of anaerobically metabolized glucose (bottom left) and glycolytic ATP (bottom right).

In a next step we considered cell cycle progression and death to depend on the yield of adenosine triphosphate (ATP), the main energy currency of a cell [54] a cell can produce from the consumed glucose and oxygen, instead of the local product of oxygen and glucose concentration:

$$\begin{array}{c} k_{div}=k_{div}^{max}H\left(p_{ATP}-p_{ATP}^{min}\right), \end{array}$$ (7)

$$\begin{array}{c} k_{nec}=k_{nec}^{max}H\left(p_{ATP}^{min}-p_{ATP}\right), \end{array}$$ (8)

where *p*_*ATP*_ is the production rate of ATP (see Eq 28), $p_{ATP}^{min}=900mM/h$ a threshold value demarcating the border between proliferating and dead states. We note that cells which are sufficiently supplied with both, oxygen and glucose, produce ATP at a rate of about 1100*mM*/*h* (see Fig 8 which seems to optimally fulfill the cells energy requirement. Interestingly, cells that moderately lack either glucose or oxygen will increase their ATP production up to 1700*mM*/*h*.

Fig 7 shows that ATP-limited growth (solid lines) is in good agreement with the tumor growth kinetics for both conditions, II and III.

The view of an important growth regulating function of ATP is supported by the observations that ATP has been shown to control the cell cycle in developing retinas of chick and mouse [55], and that the most successful drugs targeting the cell cycle inhibit ATP binding to Cyclin Dependent Kinases—Enzymes (CDKs) essential for cell cycle progression ([56, 57]). Moreover, ATP has been found to upregulate proliferation of human cardiac fibroblasts ([58]).

Nevertheless, in the case of 5mM of glucose (condition II) the model predicts a much larger necrotic core than for 25mM of glucose (condition III) while the experimental curves suggest a rather comparable size. Moreover, the fraction of dying cells for condition II is much smaller than experimentally found. The size of the necrotic core changes with the diffusion coefficients of glucose and oxygen as illustrated for another simulation example shown in Fig 7 (dashed lines). Here we assumed the molecular diffusion in the medium to be 30-fold larger than in the spheroids. In this scenario the model shows a better agreement with 5mM of glucose (condition II), but rather underestimates the size of the necrotic core for 25mM of glucose (condition III). Although the size of the necrotic core predicted by model 2 changes with the medium concentration of glucose and apparently the diffusion coefficients, the experimental curves suggest that it is approximately the same for 5mM and 25mM of glucose (compare also day 24 in Fig 3).

#### Model 3: Lactate-induced cell death

Moreover, the experimental profiles for the dying cell fraction (see Fig 3) reveal several differences between both conditions II and III. Firstly, for 5mM glucose the necrotic core starts at a depth of about 150 microns at 17 days as well as at 24 days, while for [G] = 25mM it emerges slowly: it is almost not present at day 17 and becomes big at day 24. Secondly, the transition between necrotic and viable parts is much sharper for [G] = 5mM than for [G] = 25mM. And third, after 24 days, counter intuitively, there are even more dying cells found in the exterior parts (< 150*μm*) for 25mM of glucose compared to 5mM even though the latter is supposed to be more nutrient-limited.

**Fig 9:**
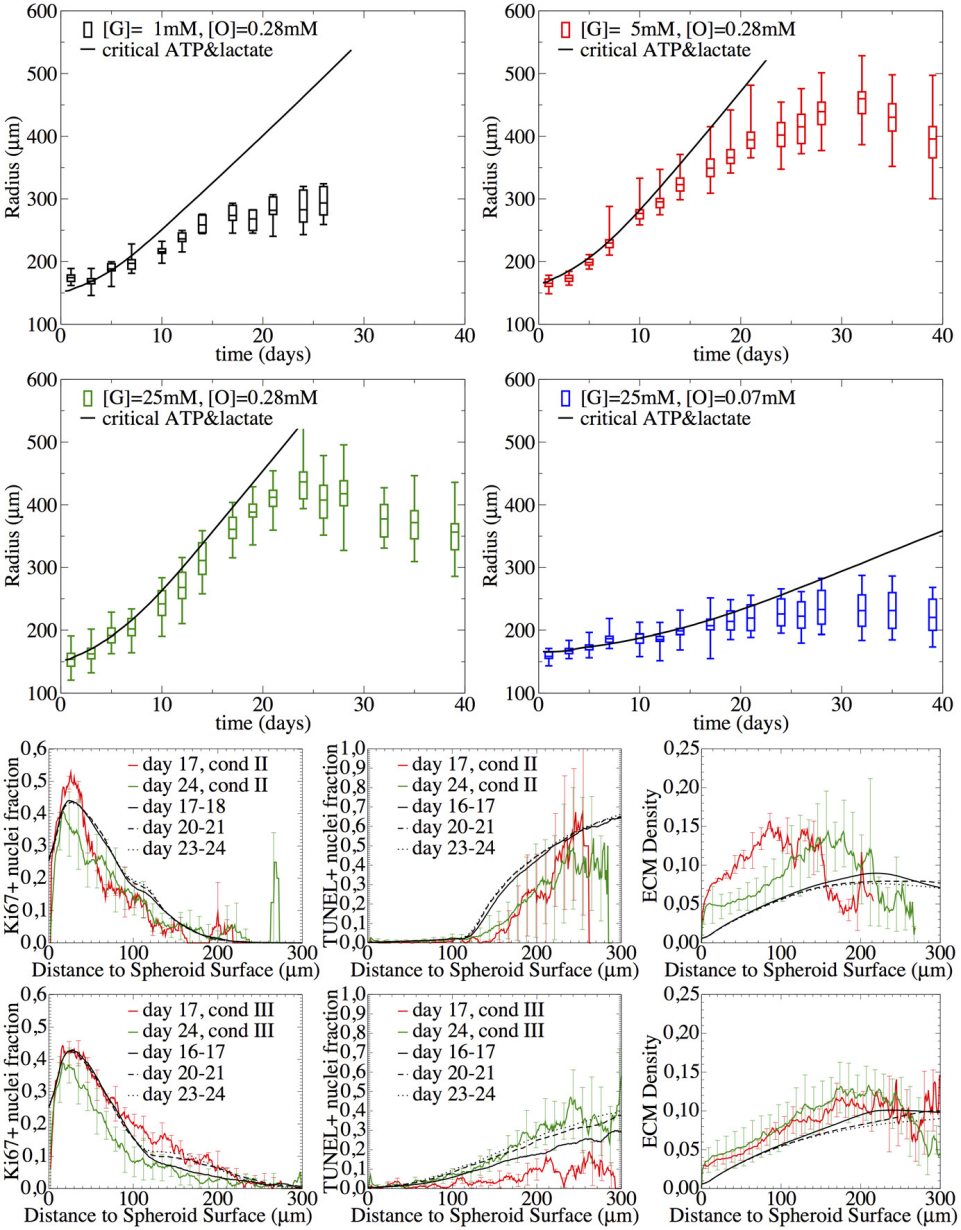
Adding lactate-induced cell death. If above a certain concentration of lactate produced during glucogenesis, cell death occurs, population kinetics and spatial temporal profiles for conditions III ([G] = 25mM, [O] = 0.28mM), and II ([G] = 5mM, [O] = 0.28mM), as well as the growth phases of condition I ([G] = 1mM, [O] = 0.28mM) and condition IV ([G] = 25mM, [O] = 0.07mM) were correctly captured. The diffusion coefficient in the medium was assumed to be D_med_ = 30 D_tum_. The experimental growth curves are shown as boxplots (box: mean, lower & upper quartils, horizontal dashes: minimum & maximum), and the radial profiles as composition of mean (line) and standard deviation (error bars).

This suggests that the cause of cell death may be different for the two conditions. Hence in a third step we studied the consequence of the additional assumption that cell death may also be triggered if the local lactate concentration overcomes a critical threshold (Fig 9). Cells being sufficiently supplied by glucose, but lacking oxygen, produce lactate. We here test the assumption that lactate might have a death-promoting effect by setting the death rate to

$$\begin{array}{c} k_{nec}=k_{nec}^{max}\cdot H\left(p_{ATP}^{min}-p_{ATP}\right)\cdot \frac{{\left[L\right]}^{n}}{{\left({\left[L\right]}^{max}\right)}^{n}-{\left[L\right]}^{n}}, \end{array}$$ (9)

where [*L*]^*max*^ = 20*mM* is the lactate concentration where the cell death rate is half its value, and *n* is the Hill coefficient which determines the sharpness of the switch from no to full cell death response. I.e. *n* = 1 leads to a smooth transition and *n* → ∞ to a Heaviside function. We have chosen the Hill coefficient such that the best fit could be obtained (Fig S8 in S1 Document). A linear dependency or a Heaviside function led to significantly worse agreement to the data than a Hill function with *n* = 2. As lactate is a side-product of anaerobe metabolism Eq 9 will mainly affect conditions with high glucose, but low oxygen supply (see Fig 8).

Fig 9 shows how the combination of ATP-limited and lactate-induced cell death impacts the growth curves for all four conditions and the radial profiles of condition II ([G] = 5mM, [O] = 0.28mM) and III ([G = 25mM, [O] = 0.28mM). Now both, shapes and temporal evolutions of the radial profiles of the proliferating and dying cell fractions are in much better agreement with the data than before.

Moreover, and interestingly, the model, although only calibrated with [G] = 5mM, [G] = 25mM and [0] = 0.28mM, quantitatively predicts the growth phase for very poor glucose medium concentration ([G] = 1mM, [O] = 0.28mM) and very poor oxygen medium concentration ([G] = 25mM, [O] = 0.07mM) without any additional parameter adjustment! This indicates that the functional dependencies on the key factors determining the growth phase of SK-MES-1 spheroids are captured.

It is interesting to notice that growth adverse effects of lactate have been observed in mouse hybridoma ([59]). High concentrations of lactate (55mM) were found to inhibit cell growth, with accompanying increase of death rates (even though the authors attribute the latter to higher osmolality). However, the overall dependency of the death rate on lactate concentration is very similar to those predicted by our model (Fig S7 in S1 Document). A similar finding has been made in CHO (Chinese Hamster Cells; [60]), immortalized cells.

#### Model 4: Waste and under-oxygenation mediating quiescence

Finally, although the initial growth phases observed for all four conditions can be well explained by ATP and lactate dependent cell dynamics, they fail to explain saturation, which occurs for all conditions after about 20 days. As for small times for all growth conditions for which [O] = 0.28mM growth is faster than linear, saturating at about 20–30 days (i.e., turning into a phase in which growth is slower than linear), there must exist a point of inflection, above which the expansion speed starts to decrease. However, as long as the nutrient concentration in the medium is maintained, the outer cells are fed by nutrients and a decrease of the expansion speed cannot be nutrient related. Hence the reduction in the expansion speed must emerge from growth inhibition originating from processes inside the tumor. Lactate can be excluded as a candidate for such a growth inhibitor as it only appears in the cases of oxygen-deprivation (III & IV). Moreover, mechanical stress can be excluded to be responsible for saturation as the environment of the multi-cellular spheroid is liquid, not a medium that responds with higher resistance upon compression ([48, 61, 62]).

We tested the hypothesis that the cytoplasmic waste material, released by lysing dying cells, may act as growth inhibitor, by the assumption that cells being exposed to waste for longer than a certain time span become quiescent:

$$\begin{array}{c} n_{exp}=\int_{-\infty }^{t}\left[1-H\left({\left[W\right]}^{max}-\left[W\right]\left(r,\tau \right)\right)\cdot H\left(\left[O\right]\left(r,\tau \right)-{\left[O\right]}^{min}\right)\right]\cdot k_{div}d\tau \geq n_{exp}^{max}, \end{array}$$ (10)

where *H* is the Heaviside function, *n*_*exp*_ is the number of cell cycles a cell was either exposed to waste material or deprived of oxygen, or both, $n_{exp}^{max}$ is the number of cell cycles a cell can maximally stay under this condition without being growth inhibited, [*W*](**r**, *τ*) and [*O*](**r**, *τ*) the local concentrations of waste and oxygen molecules, [*W*]^*max*^ and [*O*]^*min*^ the respective threshold concentrations. I.e., a cell that has accumulated too much waste becomes quiescent, and does not (re-)enter the cell cycle anymore. Notice, that this dynamics can also be expressed by a stochastic dynamics and hence incorporated into the master equation by introducing an internal state variable (counter) for each cell that increases with a certain constant rate when the external local concentration of waste overcomes the threshold concentration, and assuming cell cycle (re-)entrance to be inhibited when this state variable overcomes a critical value. If the number of states and the rate of increasing the counter by one unit are both large, the emerging waiting time until the threshold is reached is Erlang-distributed with very small standard deviation (i.e. comes very close to the deterministic limit represented by Eq 10). Instead of counting the number of cell cycles the cell is exposed to a critical concentration of waste or to insufficient oxygen (or both) as in Eq 10, we tested alternatively the condition, that the exposure is integrated over absolute time (instead of multiple of the cell cycle times). In this case, only the fit to condition IV changes by that the deviation between experiment and simulation increases by about 5%.

In summary, the transitions between proliferating and quiescence for model 4 are determined by Eqs 4 and 10.

Moreover, it can be observed that the radius expansion slows down under all four conditions after about 200…300 hours. Thus, we further assume that cells under waste exposure or oxygen deprivation grow with a reduced speed of

$$\begin{array}{c} k_{div}=k_{div}^{max}\cdot H\left(p_{ATP}-p_{ATP}^{min}\right)\cdot \left(1-0.5H\left(\left[W\right]-{\left[W\right]}^{max}\right)\right)\cdot \left(1-0.5H\left({\left[O\right]}^{min}-\left[O\right]\right)\right), \end{array}$$ (11)

where [*O*]^*min*^ and [*W*]^*max*^ are the critical concentrations of oxygen and waste, respectively. The growth rate reduction for low oxygen supply was previously reported by Stolper et al. [63], while the growth-inhibiting effect of waste originating from necrotic debris was already proposed by Greenspan [64].

**Fig 10:**
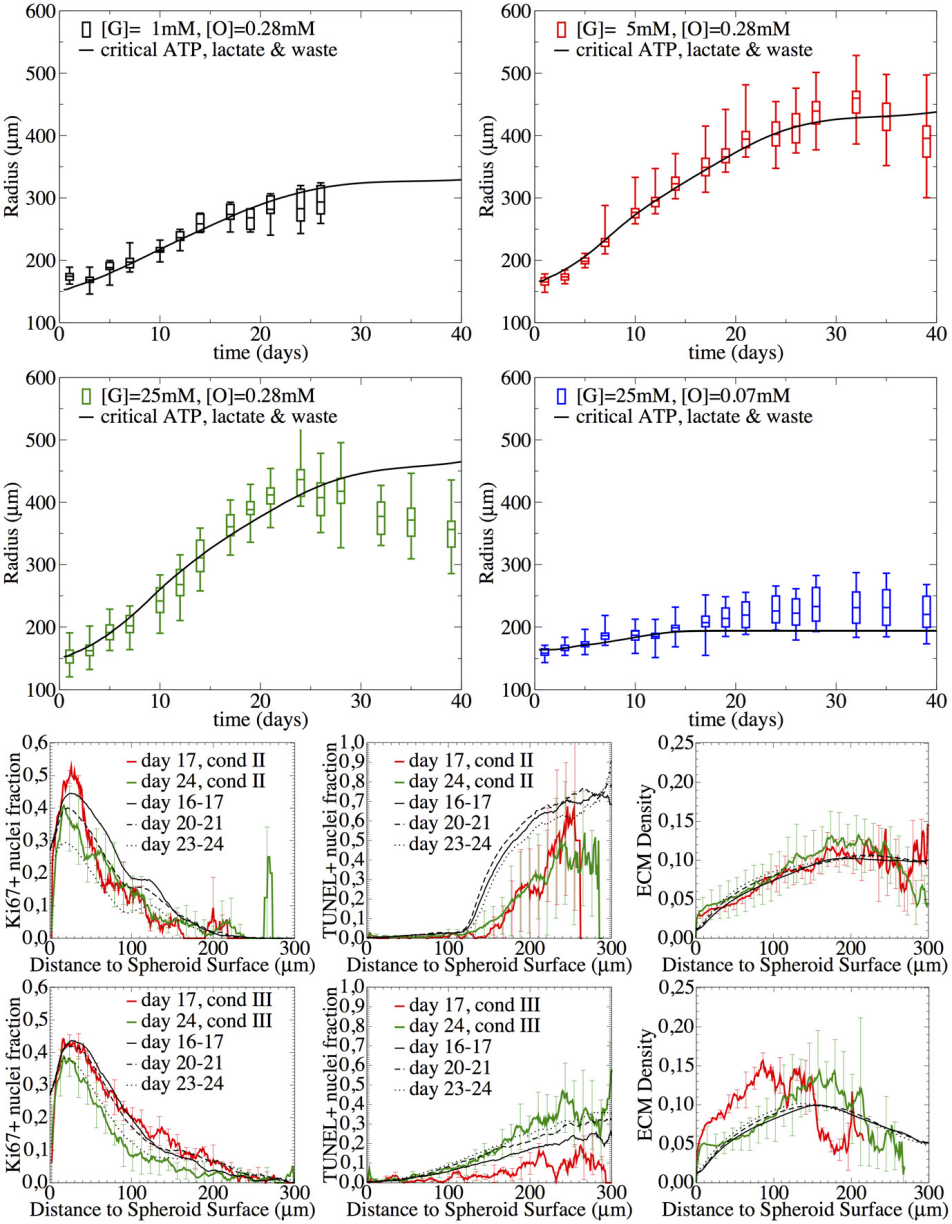
Waste & under-oxygenation mediating quiescence. If a cell was exposed to waste (cellular debris from cellular lyses of dead cells) or deprived from oxygen for a certain number of cell cycles, $n_{exp}^{max}$, it will become quiescent after its next division. The population kinetics of all conditions I-IV during all growth phases including the saturation as well as the spatial temporal profiles for conditions III ([G] = 25mM, [O] = 0.28mM), and II ([G] = 5mM, [O] = 0.28mM) were correctly captured. The graphs show a comparison between experimental observation and model simulation. The experimental growth curves are either shown as boxplots (box: mean, lower & upper quartiles, horizontal dashes: minimum & maximum), or as composition of mean (line) and standard deviation (error bar) for the radial profiles. The simulation curves are shown as black lines.

Fig 10 shows the simulation results with the extended model, including the lactate and waste dependency for two different thresholds of the waste exposure time. Condition II and III show a similar initial growth phase, followed by two linear regimes—the first dominated by ATP-limitation and the second by waste-intoxication—and finally terminating in saturation.

On the other hand, conditions I and IV saturate almost immediately after a very short initial growth phase driven by waste exposure (condition I) or oxygen deprivation (condition IV). Note that saturation is only intermittent if cell quiescence is delayed by a sufficient number of cell cycles under exposure. While for a larger delay of 8 cell cycles (Fig 10) the spheroids permanently stop to grow, for a 4 cell cycle delay (not shown) the spheroid expansion would only temporarily slow down and then recover its initial pace. This is related to the larger amount of waste, which can pile up and spread during longer delay times and thus affect all cells and not only the ones closest to the necrotic core (for simulation snapshots of all conditions see Fig S14 in S1 Document).

## Discussion

In this paper we inferred a mathematical model of tumor spheroid growth for the non-small cell lung cancer cell line SK-MES-1 from image data of growing tumor spheroids. Cell nuclei, proliferating cells, extra-cellular matrix and dying cells (by either necrosis or apoptosis) were labeled at different points in time and under different oxygen and glucose medium concentrations. The model was built by an iterative procedure, which we propose as a general template for modeling tissue organization processes. We started by developing a minimal model for one growth condition only, then stepwise extending this model by further mechanisms whenever the previous simpler model turned out to be insufficient to reproduce the experimental observations for an additional growth condition. Before adding a new mechanism to an existing model version we verified by extensive computer simulations (usually hundreds of runs), that within the parameter range for each parameter of the existing model no satisfying agreement between model and data could be achieved. Minimal is here to be understood as sufficient to explain the data and containing as least mechanisms as possible, whereby the building blocks of the model were chosen from those mechanisms that have already been described somewhere for any cell population. A similar iterative strategy was pursued for liver regeneration after drug induced damage predicting a previously unrecognized and subsequently validated order mechanisms [65].

We studied four different combinations of glucose and oxygen in the medium. To explain the growth kinetics, the proliferation, ECM, and cell death for the condition with high glucose and high oxygen medium concentration ([G] = 25mM, [O] = 0.28mM), the second with intermediate concentration of glucose and high oxygen concentration ([G] = 5mM, [O] = 0.28mM), we needed to assume that the cell cycle progression is possible only above a critical local production rate of ATP (= *mM*/*h*). A second necessary condition was, that the local density of extra-cellular matrix had to be higher than a critical value (0.003). This is in accordance to literature, where dependence of cancer progression on the ECM has been shown for skin cancer [66], breast cancer [67] and NSCLC [68], where Collagen IV can regulate crucial cell signaling. If both conditions (enough ATP and ECM) were fulfilled, cells could reenter the cell cycle after a cell division. Here, cells, which were closer to the spheroid surface and thus needed less energy in order to expand, had an increasing chance to continue proliferation and not to become quiescent. Interestingly the decision whether a cell in a certain condition became quiescent, had to be stochastic. This introduced some heterogeneity in subsets of cells in the same conditions. A deterministic scenario could not have explained the smooth transition from proliferating to quiescent zones.

The production rate of ATP depended on the local oxygen and glucose concentrations. Thereby, the ratio between both dictates to which extent a cell is in the aerobic Krebs cycle or the anaerobic lactate fermentation. Warburg stated in [69] that all cancer cells suffer from an injured respiration and thus have an exclusively anaerobic metabolism. In opposition, Zu and Guppy [70] disproved this hypothesis due to the lack of evidence and rather claimed the metabolism in cancer cells to be functional, but mainly glycolytic due to hypoxia. Here we come to a partially different conclusion: if cells are sufficiently supplied with glucose (independent from the oxygen supply), the metabolism will remain glycolytic (90%), and only if the glucose supply is getting short, the metabolism will favor the aerobic Krebs cycle (see Fig 8). Besides lactate acidity (> 20*mM*), the depletion of carbon sources to maintain a critical ATP production (= 900*mM*/*h*) and not hypoxia were the main reasons of death. The latter was also recently suggested by Kasinskas et al. [71], while, in contrast to our assumptions, they excluded lactate as source of acidity and instead assumed it to be an important secondary metabolic resource. However, either growth adverse or death promoting effects were described for high lactate concentrations [59]. So here further clarification of the dominating role of lactate would be necessary. The functional forms of the oxygen and glucose consumption rates were inferred from experimental findings of Freyer, Sutherland and co-workers in EMT6/Ro cells. The lactate and ATP production rates were then directly derived from those rates by the single assumption that cells transform the consumed glucose in an optimal way with respect to ATP output. For wide ranges of glucose and oxygen concentrations the ATP production rates remain stable between 80…130 × 10^−17^ *mol*/*cell*/*s* or 1000…1700*mM*/*h* respectively, assuming a reference cell volume of 2700*μm*. In literature values can be found between 4.6…15.3 × 10^−17^ *mol*/*cell*/*s* ([72–75]). The difference could be either due to differences in energy needs between different cell types, or to the model simplification that glucose in our model is exclusively used for metabolism.

Interestingly and importantly, the model, despite only having been calibrated with two of the four growth conditions, were subsequently able to correctly and quantitatively predict the growth phase of the other two growth conditions ([G] = 1mM, [O] = 0.28mM and [G] = 25mM, [O] = 0.07mM, respectively). This indicates that the model did capture the functionalities necessary to explain the data for different glucose and oxygen conditions. To further permit independent validation of our model, we performed additional simulations for other glucose and oxygen medium concentrations (Fig S12 in S1 Document).

However, all growth curves showed saturation and partially even shrinkage after some time. The saturation phase could be largely captured by adding the potential effect of a waste produce being released in the extracellular space from cells undergoing lysis. Shrinkage could be added if dying cells at the border detach and enter the growth medium; however, we did not consider this process, as it was not observed in the experiments (for example, for A549 cells, another NSLC cell line, a massive detachment of cells from the spheroid could be observed in the experiments). Interestingly, model simulations with a lysis rate of 0.35/h, a typical value in-vivo, turned out to be incompatible with the in-vitro data. A lysis rate of a few hours as observed in-vivo would lead to a very fast removal of dying cells and thus almost no dead cells in the tumor center, in sharp contast to the in-vitro experiments. We obtained a much smaller value of about 0.01/h by comparison of model simulation results and the spatial cell death and proliferation profiles i.e., only such a small lysis rate permits the occurrence of a “necrotic core” as observed in the in-vitro experiments. For such a low lysis rate we found that the apoptosis—if present—would need to be very slow, as it affects also cells in the viable rim in order to agree with the experimental observation of only very little dead cells in the viable rim. For this reason, apoptosis could be neglected in explaining the experimental results in this paper. The small value of the lysis rate, even though surprising on a first view, may be explained by noticing that stromal cells (such as e.g. macrophages) digesting dead cells are not present in-vitro. Hence lysis might be expected to be slower in-vitro than in-vivo.

Contact inhibition seems to be a crucial element. Suppressing contact inhibition with varying combinations of the other mechanisms in each case leads to complete failure of match between data and model simulations (see Fig S6 in S1 Document, where the parameters of model 4 has been used). This observation supports the view expressed previously in the paper that a mechanical growth inhibition plays an important role in multicellular spheroids.

We moreover tested the possibility that cells may actively migrate towards the necrotic zone by necrotaxis (Figs S9, S10 in S1 Document). As to keep a sufficiently large necrotic core as experimentally observed the lysis rate had to be small, significant migration could not be observed. On the other hand, if the lysis rate was chosen large, then significant migration of cells could be observed but the necrotic core was too small, as cells in the center were too quickly eliminated by lysis. In the latter case, the necrotic core with increasing migration rate became smaller (Figs S9, S10 in S1 Document). We concluded that migration driven by morphogens towards the central necrosis in SK-MES-1 cells is small.

**Fig 11:**
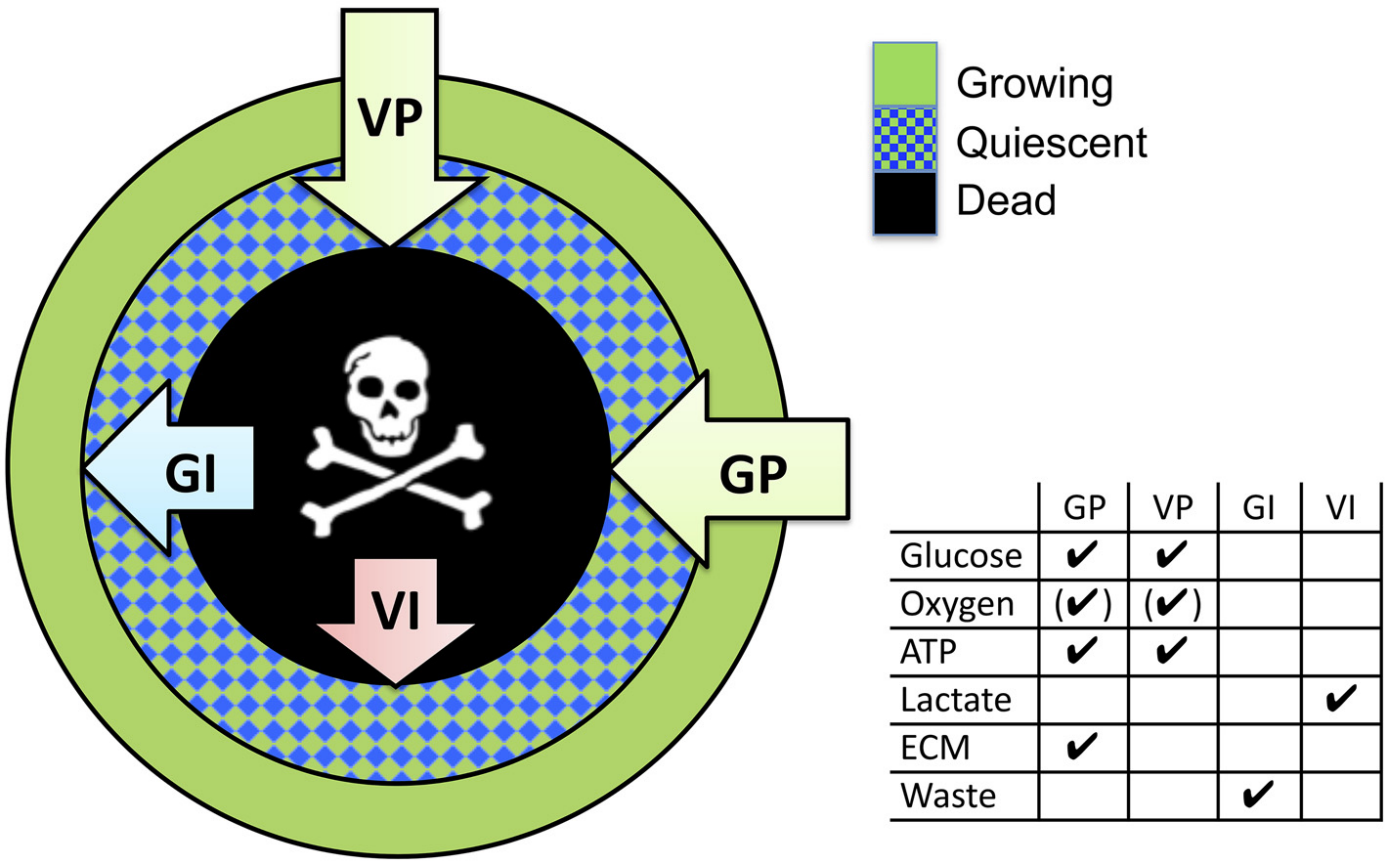
Spheroids show a radial organization of cell phenotypes: proliferating (green), quiescent (blue) and dying cells (black). The thickness of those layers is controlled by different factors as growth promoters (GP), growth inhibitors (GI), viability promoters (VP) and inhibitors (VI). The different control molecular components can largely be mapped on the control molecules emerging in our final model.

Interestingly the final model emerging from this stepwise, image-guided inference strategy closely resembles the hypothesis on growth control of MCTS by growth promoters (GP), growth inhibitors (GI), viability promoters (VP) and inhibitors (VI) (Fig 11).

In order to permit validation of our model, we simulated a number of predictions (Fig S13 in S1 Document). We predicted the spatial temporal growth dynamics for [G] = 1mM, [G] = 3mM, O_2_ = [0.28mM], and [G] = 25mM, [O] = 0.07mM. In this context we would like to remind that our model was able to predict the growth kinetics (L(t)) for [G] = 1mM, [O_2_] = 0.28mM, and [G] = 25mM, [O_2_] = 0.08mM correctly.

In order to quantify the goodness of the fits shown in Figs 7, 9 and 10 we calculated the log-likelihoods for subsets of curves (see Table 3A) as well as the ensemble of all curves (see Table 3B). We assumed that the measurement error is additive, normally distributed as well as independent and identically distributed (i.i.d.). Accordingly, the likelihood of the measured data mean *μ* given the parameter *θ* and the corresponding model prediction *x*(*θ*) is given by $L\left(\theta \right)=\prod_{i}\frac{1}{\sqrt{2\pi \sigma_{i}^{2}}}exp(\frac{{\left(x{\left(\theta \right)}_{i}-\mu_{i}\right)}^{2}}{2\sigma_{i}^{2}})$, where the index *i* runs over the data points. The uncertainty of the data points is determined by the standard deviation, *σ*. Accordingly, points with large uncertainties *σ* are weighted less. Despite some deviations in single profiles, the final model considering ATP, lactate and waste is found to be the most likely to explain the experimental data. On the other hand, an increase of the likelihood correlates with the increasing number of model parameters and the risk of over-fitting. Especially model 2 and 3 have a small relative difference in log-likelihood. As a measure for model quality accounting for the number of parameters we used the Akaike information criterion (AIC) (see Table 3D), which also confirms the final model to be the best choice. We note that the number of parameters had no influence on the ordering of the models as the absolute differences of their log-likelihoods Δln*L* is many orders of magnitude larger than the difference in number of parameters Δ*k* (see Table 3B and 3C).

**Table 3:** Model comparison. The table shows the log likelihoods (A, B) and the Akaike information criterion (D) comparing three models to experimental data. The growth curves of all nutrient conditions and the radial profiles of proliferation, death and ECM at a specific time were compared individually (A) as well as a whole (B, D). We also calculated the values according to the Bayesian information criterion (BIC), which led to the same ordering (not shown). Notice that the penalty terms in the AIC (2*k*) and BIC (*k* log(*n*), *n*: number of data points) play no role in the evaluation as the difference in the penalty terms are much (4–5 orders of magnitude) smaller than ln *L*. (Δ AIC = AIC(Model row-index)—AIC(Model column-index).)

	**Log-likelihood**	**(Model 2)**	**(Model 3)**	**(Model 4)**	**#Data Points**
	ln *L*	ATP	ATP & Lactate	ATP, Lactate & Waste	*n*
A	growth curves (all conditions)	-8.61e+02	-7.42e+02	-1.72e+02	60
	proliferation (conditions II & III, day 17)	-1.83e+05	-1.60e+05	-1.83e+05	460
	death (conditions II & III, day 17)	-2.69e+03	-5.15e+03	-6.16e+03	532
	ECM (conditions II & III, day 17)	-1.23e+06	-1.23e+06	-8.66e+05	524
	proliferation (conditions II & III, day 24)	-1.97e+02	-1.40e+02	1.18e+02	532
	death (conditions II & III, day 24)	-7.47e+02	2.16e+01	-3.11e+02	616
	ECM (conditions II & III, day 24)	-1.82e+01	3.08e+01	3.26e+02	609
B	total	-1.42e+06	-1.39e+06	-1.06e+06	3333
C	#model parameters *k*	reference	+2	+9	
D	(Model 2)	0.00E+00	-5.00E+04	-7.30E+05	
	ΔAIC = 2Δ*k* − 2Δln *L* (Model 3)	5.00E+04	0.00E+00	-6.80E+05	
	(Model 4)	7.30E+05	6.80E+05	0.00E+00	

To conclude, we would like to stress the key message demonstrated in this paper: quantitative comparison of spatial profiles observed from cells states in different experimental conditions and time-points, generates information so rich that one may infer even molecular control mechanisms and parameters of spatio-temporal growth and death patterns. Hence, careful imaging, image processing and image analysis may serve as an important source of information to infer mechanistic knowledge on tissue growth and organization processes. Such an approach would gain to be more fully explored.

Our model is hybrid. It integrates as separate components the cell and molecules, and as functional components a mechanical form of contact inhibition, a metabolic component comprising oxygen, glucose, ATP, lactate, and waste, several of the molecules acting as morphogens. It would be interesting to see in how far the same model can capture the growth behavior of other cell lines and of other cell types.

We think that the possible imaging techniques and image analysis software in combination with modeling could permit a screening of growth dynamics and subsequent quantitative classification of multicellular spheroids. For example, EMT6/Ro cells ([14]) show a very similar but not equal growth phenotype as SK-MES-1 cells: (1) detachment of cells is rare (as opposed to, for example, A549 cells, that reveal significant detachment in-vitro (and in-vivo)), (2) under sufficient oxygen supply, the growth of the outer spheroid diameter remains unaffected (or almost unaffected) by glucose, while (3) reduction of oxygen from 0.28mM to 0.07mM reduces growth dramatically in SK-MES-1 cells even if glucose medium stays high, while in EMT6/Ro cells no reduction is observed as long as nutrient medium concentration stays high: this demarcates a difference between the EMT6/Ro and SK-MES-1 cells. On the other hand, glucose affects the size of the necrotic core. Reduction from 16.5mM glucose to 0.8mM glucose medium concentration in EMT6/Ro cells (at 0.28mM oxygen) increases the necrotic core (as one can infer from comparing the cell count with the diameter, see [34]), which can also be observed in SK-MES-1 cells if glucose is reduced from 25mM to 5mM (at 0.28mM O_2_). However, in SK-MES-1 cells the necrotic core for richer glucose ([G] = 25mM vs. 5mM) occurs later but at about 24 days is about the same size.

Models can provide a quantitative framework to test how far such differences can be attributed to parameters with the same model, or whether “another” model needs to be used by adding or dropping mechanisms. For example in the first case, can the same model be used to capture a wide range of cell lines with regard to their MCS growth behavior by only adjusting its parameters—indicating only quantitative changes, or, in the 2nd case, does one need to implement mechanisms for one cell type that are not observed within the physiological range of parameters for another cell type? Given how much multicellular spheroids are still in use as biological model system, we think it would be of fundamental interest to do such an analysis as a community effort, even though this might be considered as on the first view as a “step-back” as the growth dynamics of multi-cellular spheroids could have been measured 20—30 years ago. Modern technology could largely permit automated analysis if pipelines were constructed for that purpose, hence avoiding the largely manual and tedious analysis applied for the work in this paper. Adding more and more cell lines would permit to refine the model one starts with, and zoom into the so far still highly simplified representation of mechanisms without the threat of having a far to large number of fit parameters that cannot be controlled. In this way, identification of necessary model components and adjustment of parameters linking the components could be achieved.

## Materials and Methods

### Cell Culture

NSCLC cell line SK-MES-1 used in this study was obtained from ATCC (Manassas, VA, USA) and cultivated in a humidity controlled incubator at 37 C and 5% CO_2_ in 150cm^2^ tissue culture dishes (TPP) in DMEM (Dulbecco’s modified Eagle’s medium, LONZA, Verviers, Belgium) supplemented with 10% FCS (fetal calf serum, Southern America, GIBCO, Germany) and 1% Penicillin/Streptomycin (Biochrom AG, Berlin, Germany). Cells were used between passages 10 and 30 and passaged at a split ratio of 1:4 to 1:6. Cultures were routinely tested for mycoplasm contamination as described by Stacey and Doyle 1997 and always found to be negative. Additional medium for the test cultures was DMEM w/o Glucose (GIBCO, Germany) supplemented with 10%FCS and 1mM Glucose (Carl Roth GmbH, Germany), and DMEM with 1.0 g/L glucose w/o L-Glutamine (LONZA, Verviers, Belgium) supplemented with 10% FCS and 25mM L-Glutamin (SIGMA, Germany). Additionally, cells were kept in a humidity controlled incubator at 37 C and 5% CO_2_ and either normal atmospheric 20% O_2_ (corresponding to 0.28mM) or 5% O_2_ (corresponding to 0.07mM). This choice permits comparison to classical work in literature (e.g. [12]) and takes into account that lung is rich in oxygen. NSLC cells originate from lung epithelium having at least partially direct contact to the inhaled air so are at least initially not limited to the blood oxygen level.

#### Solutions

2% Methocel solution was prepared by stirring 6g Methylcellulose (SIGMA, Germany) in 250ml propagation medium at 60 C for 20min. Then a further 250ml of medium were added and stirred at 4 C over night. The solution was aliquotted in 50ml Falcon tubes, centrifuged two times for 99min at 4000 rpm to concentrate long Methylcellulose-Fibers at the bottom of the tube. Tubes were then stored upright at 4°C.

2% Agar-Solution was prepared by solving 5g bacterial grade Agar (GIBCO, Germany) in 250ml of H20. This solution was autoclaved and then kept at 60 C until 24 well-Plates were coated.

#### Spheroid generation and cultivation

For the generation of spheroids with defined size and cell number, a hanging drop assay was employed. Here cells were first trypsinized, counted, then centrifuged and resuspended in propagation medium with 20% Methocel-Solution. Drops of 20*μl* of this cell suspension were pipetted on the lid of a 150*cm*^2^-culture dish, which was subsequently carefully inverted back onto the dish. In this approach, all suspended cells in the resulting hanging drop contribute to the formation of a single spheroid. After 48h in the hanging drop the spheroids were transferred on 24 well plates, by carefully pipetting with a cut 200ml pipette tip, with one spheroid in one ml of respective medium per well. The wells were pre-coated with 250*μl* of 2% agar each to prevent attachment of spheroids. Medium was changed once a week. Four different glucose/oxygen conditions were employed (see Table 1). Spheroids were cultivated over a period of over 48 days.

### Data Acquisition

#### Spheroid growth curves

Growth of spheroids was monitored by acquisition of bright field images through an Olympus IX-70 microscope fitted with an AxioCam Erc5s camera (Zeiss, Germany) twice a week. The projected area of spheroids was determined using ImageJ, software version 1.43u. Mean areas and standard deviations were calculated using Microsoft Excel. At least 4 spheroids were evaluated per time point and condition.

#### Cryosectioning and immunofluorescence staining

Spheroids were taken out at specified time points (see Table 4), embedded in TissueTek (SAKURA Finetek, Netherlands) cryo-medium, frozen over liquid nitrogen and processed for cryostat sectioning. Cryosections of 6–8 *μm* thickness were mounted on slides, air dried and then fixed with 4% PFA for 20 min at RT, washed in PBS for 30 min at RT, permeabilized with ice cold 0.1% TritonX-100 / 0.1% sodium citrate for 2min on ice. To stain for dead cells we employed the In Situ Cell Death Detection Kit (No. 12 156 792 910 Roche Applied Science) according to the manufacturer’s protocol. Staining for proliferating cells and ECM component Collagen IV was performed with anti human Ki67, mouse monoclonal (No. M7240 DakoCytomation, Glostrup, Denmark) and anti Collagen IV, rabbit polyclonal (No. 10760, Progen, Heidelberg, Germany) antibody in 12% BSA respectively. Secondary antibodies used were anti mouse, donkey, Cy3 (No. 715-166-151, Dianova, Hamburg, Germany) and anti rabbit, goat, Alexa 488 (No. A-11034 Molecular Probes USA/NL) in 12% BSA. In both cases, nuclei were stained with HOECHST (Bisbenzimide H 33258 SERVA, Heidelberg, Germany) in 12%BSA. Sections were examined and photographed using a Leica DM RBE (Leica, Germany) microscope fitted with epifluorescence optics.

**Table 4:** Timepoints of cryosectioning.

**Name**	**Time**
T3	17days
T4	24days
T5	34days
T6	46days

### Quantitative Image Analysis

The images acquired (see above) are raw data. They consist of a set of pixels with position $\left(x,y\right)\in N^{2}$ and color intensities for the three color channels red, green and blue defined by

$$\begin{array}{c} \begin{array}{cc} & I^{channel}\left[x,y\right]\in \left[0,1\right),\\ & I^{channel}:N^{2}\to R, \end{array} \end{array}$$ (12)

where *channel* ∈ {*red*, *green*, *blue*}. In the following, the tools used to preprocess the raw images (e.g. to reduce noise) and to identify or segment objects (e.g. cell nuclei, spheroid border) will be introduced. Then the preprocessed images have been analyzed quantitatively as described subsequently.

#### Nuclei segmentation

The cell nuclei were stained with HOECHST staining (blue color channel, see Fig 2). In order to segment the single nuclei, the images were smoothed by a series of four median filters with kernel size 3 × 3 (see below) as a preprocessing step. A larger kernel would smooth the image too much. In the following, the watershed algorithm(see below) was applied to the inverted blue color channel, 1 − *I*[*x*, *y*] (*I*[*x*, *y*]∈[0, 1)) for pixels above a certain threshold *I*[*x*, *y*]^*blue*^ > *I*^*HOECHST*^ (see Table 4). Introduction of a threshold avoids the segmentation of the whole image, focusing only on regions clearly stained by HOECHST and thus associated with the cellular nuclei.

#### Segmentation (watershed)

The watershed algorithm is a segmentation algorithm separating an image into different regions using the concept of watersheds. It was first proposed in ref. [76], and later enhanced by a fast algorithm presented in ref. [77]. The idea of the algorithm is to treat the images as landscapes, where color intensities *I*[*x*, *y*] correspond to the amplitude. The landscape representation of an example image is shown in Fig 4. The local minima of the landscape are identified with catchment basins where water would accumulate. To detect the pixels belonging to the different basins, the landscape is successively “flooded” and pixels of the current altitude are associated to the basin of their neighboring pixels (already associated to a basin). When two basins touch each other a watershed line between them is created and expansion is stopped. The algorithm either stops at a chosen maximum altitude (color intensity), or when none of the basins can expand anymore.

#### Noise-reduction (median filter)

As each local minimum is giving rise to one basin, it is important to smooth the images thereby reducing noise in the image before performing the segmentation in order to avoid an over-segmentation into many fragmented pieces. For this purpose a number of different linear (e.g. mean filter, Gaussian blur) and non-linear filters (median) were applied. We used a sequence of median filters known to result in an edge-preserving smoothing, i.e. while removing noise it conserves the original features of the image (e.g. nuclei shapes) in contrast to, for example, Gaussian blur. The impulse response *I*′[*x*, *y*] for each input pixel (*x*, *y*) is the median of neighboring entries within a certain ‘window’ of size *n* × *n*:

$$\begin{array}{c} I^{\prime }\left[x,y\right]=median_{|x-x^{\prime }|\leq a,|y-y^{\prime }|\leq a}\left\{I[x^{\prime },y^{\prime }]\right\}, \end{array}$$ (13)

where *a* = (*n* − 1)/2.

#### Spheroid lumen and border

The basis for estimating the spheroids borders was the cell nuclei (see above). By applying a series of dilation filters a coarse approximation of the cell shapes around the nuclei was achieved. A dilation can be described as the following:

$$I^{\prime }\left[x,y\right]=max_{|x-x^{\prime }|\leq a,|y-y^{\prime }|\leq a}\left\{I[x^{\prime },y^{\prime }]\right\}.$$

Finally, the empty spaces (necrotic areas) in the center of the spheroids were removed by hole-filling algorithms (e.g. by flood filling). Fig 4 shows the estimated spheroid lumen around the cell nuclei in green color for an example picture. The spheroid border is assumed to be the interface between the green (lumen) and the black (background) area(s).

#### Radial profiles, binning and averaging

As the spatial arrangement of cell phenotypes (proliferating, quiescent and dead) and ECM density could be assumed to be approximately concentric, the following sections focus on how to extract the spatial statistical profiles of the quantities stained. As Fig 4 shows, the spheroids were not completely spherical, but typically slightly elongated with irregular surfaces. To account for this, all statistics have been performed as a function of distance to the spheroid border, defined by Δ*L*, as opposed to the distance to the center of mass, which would be the method of choice in case the MCTSs would have been perfectly spherical. For estimation of the radial profiles binning was utilized. I.e. Δ*L* was divided into small intervals Δ*L*_*i*_ = [*h*(*i*), *h*(*i* + 1)), so-called bins, of *h* = 1*μm* length (image resolution: pixel = 0.98*μm*^2^). Then each nucleus that contains at least one pixel, whose distance to the closest border pixel is Δ*L* ∈ Δ*L*_*i*_, enters into the statistics of bin *i*. The average curves were generated from the radial profiles of the individual images by calculating the average values of the corresponding bins with the same index.

#### Nuclei density and cell diameter

**Fig 12:**
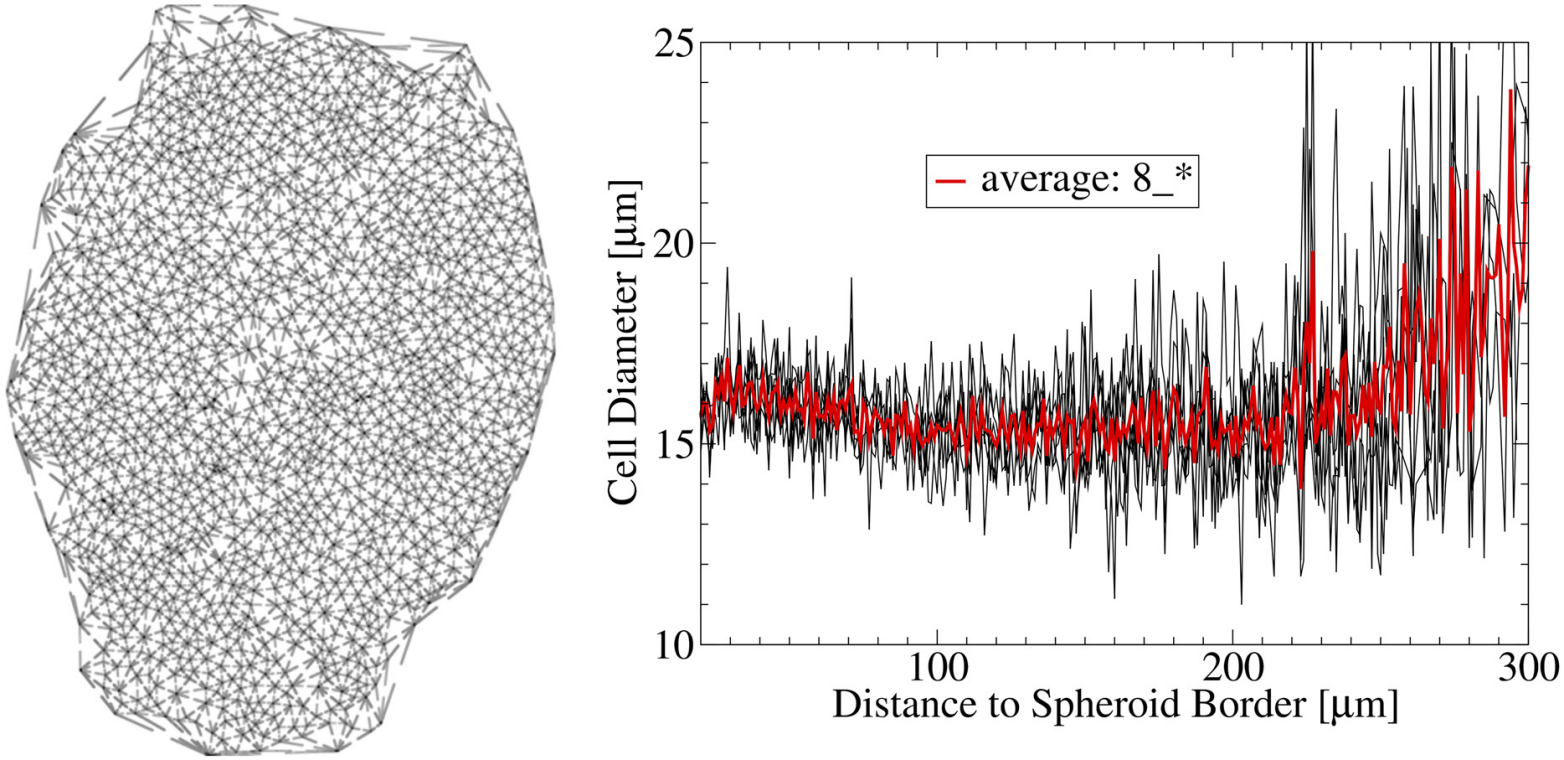
Cell density and cell size estimation. Left: The Delaunay triangulation of all segmented nuclei serves to estimate the cell sizes via its dual, the Voronoi diagram. Right: Average cell diameter as a function of distance to the spheroid border. The black curve is the average profile of six images (condition III, T3) with bin size 1*μm* and the red curve is the gliding average with window size 10*μm*.

The local cell density can be estimated from the number of nuclei per area. Its inverse is the area per nucleus. The nuclei served as construction points for a Voronoi diagram. In a Voronoi diagram all points closer to one construction point than to any other construction point belongs to one Voronoi cell. Voronoi cells are polygonal in shape. Accordingly, the Voronoi cell belonging to one nucleus was identified with the 2D projection area of the cell having that nucleus. We estimated the cell diameter to be 16.8 (±0.6)*μm* (Fig 12).

#### Automated staining detection: Thresholding

Beside HOECHST staining for all nuclei, the sliced MCTS were specifically stained for proliferation using Ki67 and cell death using TUNEL.

In order to quantify cell proliferation, Ki67 positive nuclei (red) had to be distinguished from Ki67 negative nuclei (blue only). Moreover background noise (due to staining from different layers, image artifacts, etc.) had to be eliminated to minimize the number of “false positive” and“false negative” nuclei.

Via an intensity threshold, *I*^*Ki*67^, we accepted a pixel (*x*, *y*) to be Ki67 positive only if its red color intensity *I*^*red*^(*x*, *y*) was above a certain value:

$$\begin{array}{c} Ki67\left(x,y\right)=\left\{\begin{array}{c} 1,\text{if}\;I^{red}\left(x,y\right)\geq I^{Ki67},\\ 0,\text{else}. \end{array}\right. \end{array}$$ (14)

Via the fractional threshold, *φ*^*Ki*67^, we decided whether a nucleus *X* typically composed of many pixels was Ki67 positive or not:

$$Ki67(X)=\{\begin{array}{l} 1,\text{if }\frac{1}{\sum_{(x,y)\in X}1}\sum_{(x,y)\in X}Ki67\left(x,y\right)\geq \varphi^{Ki67},\\ 0,\text{else}. \end{array}$$ (15)

Parameters *I*^*Ki*67^ and *φ*^*Ki*67^ control the classification and had to be chosen such that the results minimize the number of false negative and false positive nuclei.

#### Choice of detection thresholds

**Fig 13:**
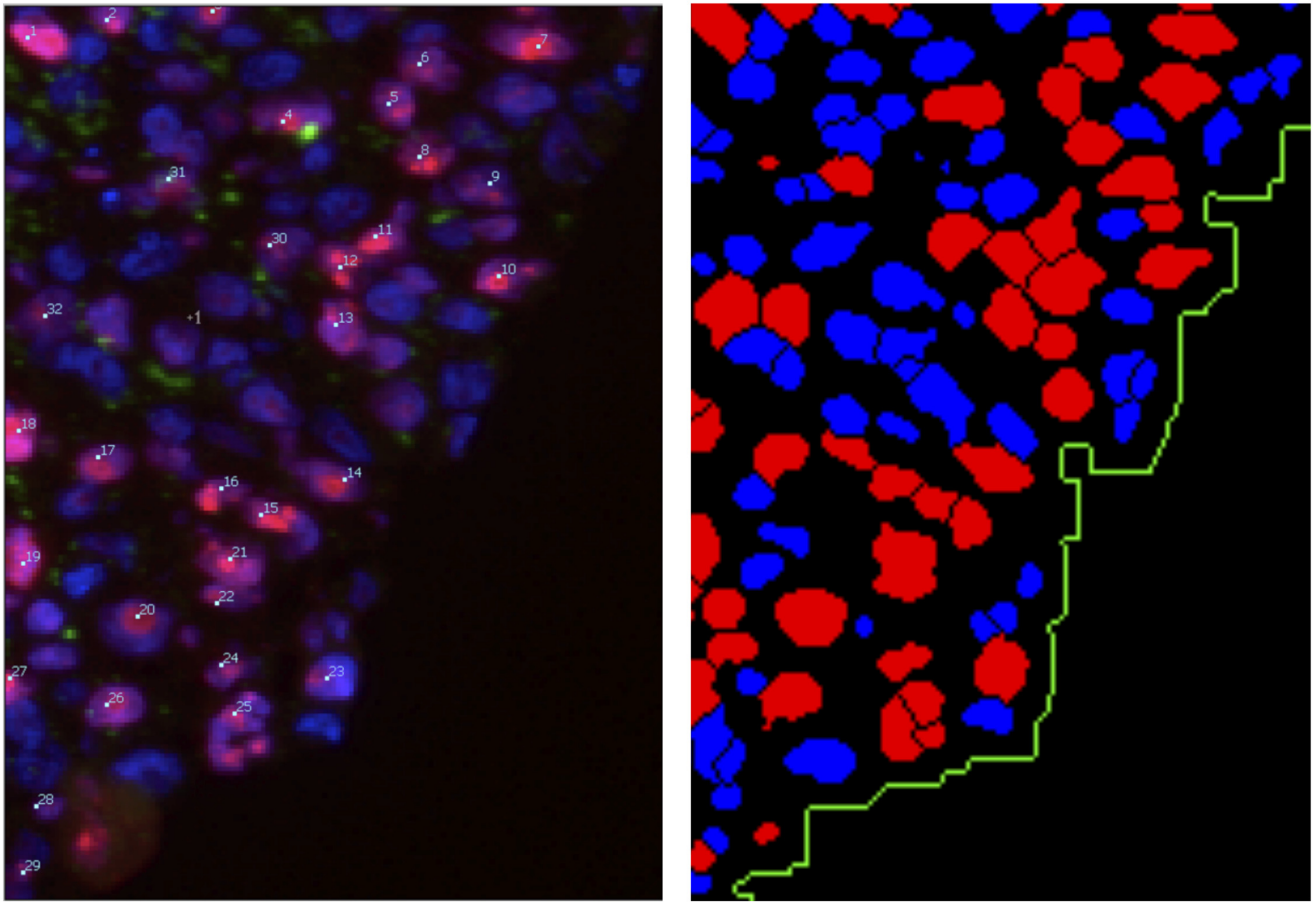
Automatic versus manual detection of Ki67 positive nuclei.

**Fig 14:**
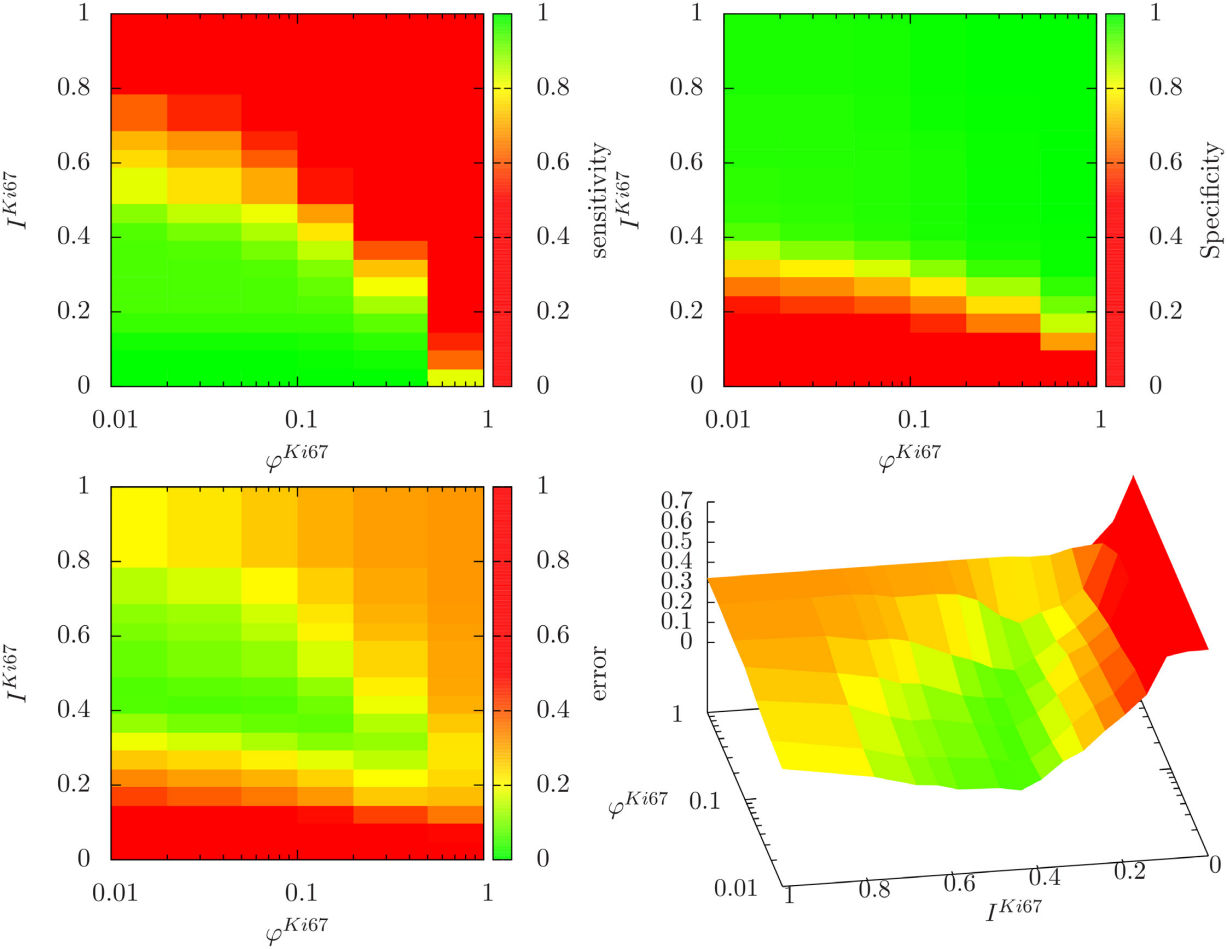
Sensitivity analysis for threshold parameters *I*^*Ki*67^ and *φ*^*Ki*67^. The plots show the sensitivity, specificity and classification error comparing the manual detection with the automated detection for a combination of {*I*^*Ki*67^, *φ*^*Ki*67^}.

The threshold values were chosen such that sensitivity and specificity of the classification were simultaneously and robustly maximized. The sensitivity (TPR = true positive ratio) is given by

$$\begin{array}{c} TPR=\frac{TP}{TP+FN}, \end{array}$$ (16)

where TP denotes the true positive and FN the false negative nuclei. The specificity (TNR = true negative ratio) is defined by

$$\begin{array}{c} TNR=\frac{TN}{TN+FP}, \end{array}$$ (17)

with TN being the true negative, FP the false positive nuclei. A “perfect” method would yield specificity and sensitivity both being one, i.e., no false negative and no false positive would be detected. In order to distinguish between TP, FN, TN and FP, a reference (“gold standard”) is needed. For this purpose part of the images were manually evaluated for Ki67-positive (true positive) and Ki67-negative (true negative) nuclei (Fig 13). Subsequently, the threshold values of the automated image analysis method were calibrated such that TPR and TNR were maximized. This was true in a certain range for both threshold values (Fig 14). Simultaneous optimization of TPR and TNR minimizes the classification error given by

$$\begin{array}{c} \epsilon =\frac{FP+FN}{TP+FP+TN+FN}. \end{array}$$ (18)

Fig 14 shows the values of TPR, TNR and *ϵ* applying automated detection to the benchmark image in Fig 13 (left) for wide parameter ranges of *I*^*Ki*67^ and *φ*^*Ki*67^.

A good agreement (small classification error) between automated and manual Ki67 detection was reached for 0.35 ≤ *I*^*Ki*67^ ≤ 0.5 and *φ*^*Ki*67^ ≤ 0.2. The parameter values used in the following are denoted in Table 5 and the corresponding comparison of manually and automatically detected Ki67-positive nuclei is shown in Fig 13.

**Table 5:** Image processing parameters.

**Parameter**	**Value**
*I*^*HOECHST*^	0.15
*I*^*TUNEL*^	0.45
*φ*^*TUNEL*^	0.20
*I*^*Ki*67^	0.35
*φ*^*Ki*67^	0.05

### Cellular Automaton

The cellular automaton model, which extends on previous work ([43, 47]), is defined by a set of rules:

#### Cell

One biological cell can occupy either one or two sites on a Voronoi diagram (Fig 5, Fig S1 in S1 Document). The term Voronoi lattice site is used from now on to distinguish the Voronoi cell from a biological cell. At the beginning of the cell cycle, i.e. after division, it always occupies only one Voronoi lattice site. Later in the cell cycle (specified below) it grows by occupying a neighboring lattice site as well.

#### Cell cycle progression/replication

The cell cycle is subdivided into *m*_*d*_ intervals (Fig 5). At the beginning of the cell cycle the cell internal counter *M* is set to *M* = 0. If the nutrient conditions are permissive for proliferation according to the conditions detailed in the main text and in the supporting information (S1 Document) then a cell enters the cell cycle. This was mimicked by the transition *M* = 0 → *M* = 1. A proliferating cell successively increased its internal counter starting from *M* = 0 with a rate *m*_*d*_ *k*_*div*_ by one until *M* = *m*_*d*_. Here *k*_*div*_ = *τ*^−1^, where *τ* is the expectation value of the cell cycle time, and depends on the environmental conditions (see Eq 11). Note that the transitions are Poisson processes; the emerging total distribution of cell cycle time is an Erlang distribution [43].

#### A) Cell growth

At *M* = *m*_*g*_ ≤ *m*_*d*_ a cell grows by occupying a neighbor Voronoi lattice site in addition to the one it occupies already. Consequently, for the choice *m*_*g*_ < *m*_*d*_ the doubling of the mass took place somewhere in the cell cycle but not in the moment of cell division as it is the case in the classical Eden model [78] or more recent work [43]. If all neighboring lattice sites are already occupied, then the cell first liberates one of them by pushing adjacent cells toward the closest free lattice site and then occupies the original site and the liberated neighbor site (for details see supplementary material).

#### B) Cell division

When *M* = *m*_*d*_ the cell splits into two daughter cells with each of the daughter cells occupying a single Voronoi lattice site. After the division each cell decides whether to reenter the cell cycle or to become (permanently) quiescent. The probability to reenter the cell cycle, *p*_*re*_, depends on the environmental conditions (see Eq 4).

#### Cell death

Under certain conditions specified in the main body of the text a cell dies with the rate *k*_*nec*_ (see Eq 9).

#### Cell lysis

Dying cells are lysed with the rate *k*_*lys*_. Consequently the corresponding Voronoi lattice sites are set free.

#### Cell migration

Cells are able to move either by being pushed if another cell grows, or by active migration. Active migration is mimicked by hopping to a neighboring Voronoi site. The choice of the hopping rate depends on the considered biological process:

#### A) Random walk/free diffusion

In the case of unbiased cell movement the hopping rate has been chosen to *λ*_*i*_ = *λ*/*n* for all *n* neighboring sites *i* = 1…*n*. This corresponds to a diffusion process such that *λ* = 6*D*/*l*^2^, with *D* the diffusion coefficient and l the average distance between two adjacent lattice sites.

#### B) Biased random walk

The dynamics of the multi-cellular configuration is determined by a master equation for the multivariate probability to find a certain multi-cellular configuration, *X* where *X* = {*x*_1_, *x*_2_, …} denotes the state vector of the multicellular configuration. One way is to enumerate all lattice sites and denote by *x*_*k*_ the state vector of the cell localized at lattice site *k*. If this lattice site is empty, the state is zero. The dynamics is then formalized by

$$\begin{array}{c} \frac{\partial p(X,t)}{\partial t}=\sum_{X^{\prime }}e^{\frac{-\Delta E_{X^{\prime }\to X}}{F_{T}}}\cdot {\left(\tau_{X^{\prime }\to X}\right)}^{-1}\cdot p\left(X^{\prime },t\right)-e^{\frac{-\Delta E_{X\to X^{\prime }}}{F_{T}}}\cdot {\left(\tau_{X\to X^{\prime }}\right)}^{-1}\cdot p\left(X,t\right). \end{array}$$

In the case of cell-cell adhesion Δ*E*_*X* → *X*′_ represents the energy difference resulting from a change of cell-cell contacts when moving from the current (configuration *X*) to the new lattice site (configuration *X*′). *p*(*X*, *t*) is the probability of the system being in configuration *X* at time *t*. τ_y → z_ denotes the time between two successive hops from y → z. F_T_ is a characteristic fluctuation energy [1]. An alternative explained in the supplementary information is to enumerate all cells, and for each cell the processes it can perform. A process is then chosen with a probability that corresponds to its relative weight, calculated as the rate for this process divided by the sum of the rates for all other processes (for algorithmic details, see supplementary information).

### Metabolism

Glucose and oxygen are among the main metabolites of most biological cells. In normal tissue they are provided mainly by the vascularization. Tumor spheroids as avascular tumors (in-vivo) are mainly fed by nutrients diffusing into the interior from the border. Above a certain size they display regions lacking glucose (hypo-nutrition) and/or oxygen (hypoxia). This occurs if the nutrients entering the tumor via its borders are consumed completely before reaching the center.

We modeled the diffusion and uptake of glucose and oxygen by a reaction-diffusion equation:

$$\begin{array}{c} \partial_{t}u=\nabla \cdot \left(D_{u}\left(\sigma \right)\nabla u\right)+r_{u}\left(\sigma ,u\right), \end{array}$$ (19)

where *D*_*u*_ is the molecule diffusion coefficient and *r*_*u*_(*σ*, *u*) denotes the reaction term, *u* ∈ {*G*, *O*}. The diffusion coefficient was chosen differently in the nutrient medium than inside the tumor spheroid. The reaction term mimicked the cells consumption with the choice

$$\begin{array}{c} r_{u}\left(\sigma ,u\right)=-q_{u}\left(u\right)\sigma =-q_{u}\left(u\right)\sum_{k}\delta \left(r-r_{k}\right). \end{array}$$ (20)

#### Glucose and oxygen consumption rates

In experiments on EMT6/Ro cells for varying medium concentrations the cell consumption rates for each of the two molecules were observed to depend on each other ([12, 79]). Casciari et al. [80] proposed an extended Michaelis-Menten-like consumption rate for glucose,

$$\begin{array}{c} q_{G}=V_{G}^{max}\frac{\left[G\right]}{\left[G\right]+k_{G}^{G}}, \end{array}$$ (21)

and oxygen,

$$\begin{array}{c} q_{O_{2}}=V_{O_{2}}^{max}\frac{\left[O_{2}\right]}{\left[O_{2}\right]+k_{O_{2}}^{O_{2}}}. \end{array}$$ (22)

**Fig 15:**
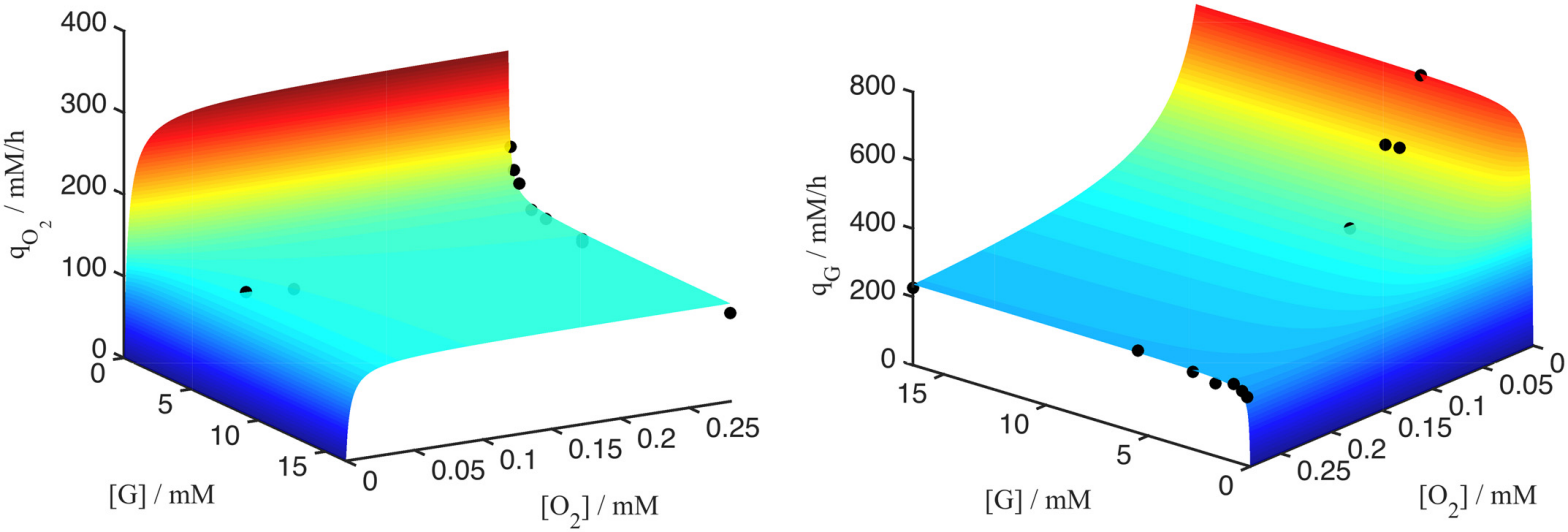
Oxygen (right) and Glucose (left) consumption. The graphs show a comparison between experimental measurements of glucose and oxygen consumption rates of EMT6/Ro cells [80] (dots) and predictions of Eqs 21 and 22 (grid). The rates are functions of the local extra-cellular glucose and oxygen concentrations. (The original values were rescaled from *mol*/*cell*/*s* to *mM*/*h*(= *mol*/*m*^3^/*h*) for an average cell volume of 2700*μm*: 1*mol*/*cell*/*s* = 13.3 × 10^17^ *mM*/*h*.)

By direct comparison to their data we found the following cross-linking terms to fit well the experimentally observed consumption rates (Fig 15):

$$\begin{array}{c} \begin{array}{cc} V_{G}^{max} & =q_{G}^{max}\left(1-\left(1-\frac{q_{G}^{min}}{q_{G}^{max}}\right)\frac{\left[O_{2}\right]}{\left[O_{2}\right]+k_{G}^{O_{2}}}\right),\\ V_{O_{2}}^{max} & =q_{O_{2}}^{max}\left(1-\left(1-\frac{q_{O_{2}}^{min}}{q_{O_{2}}^{max}}\right)\frac{\left[G\right]}{\left[G\right]+k_{O_{2}}^{G}}\right). \end{array} \end{array}$$ (23)

Within this paper we assumed the same functional form and parameter values for the consumption rates of the SK-MES-1 cells as no equivalent measurements as those for the glucose and oxygen consumption rates of EMT6/Ro cells were available for SK-MES-1 cells. The parameters of Eqs 21, 22 and 23 were inferred from [12] (see Fig 15 and Table 6).

**Table 6:** Model parameters for glucose and oxygen consumption.

**Parameter**	**Unit**	**Value**
$k_{G}^{G}$	*mM*	0.068
$k_{G}^{O_{2}}$	*mM*	0.031
$q_{G}^{min}$	*mM*/*h*	186.67
$q_{G}^{max}$	*mM*/*h*	706.67
$k_{O_{2}}^{O_{2}}$	*mM*	0.031
$k_{O_{2}}^{G}$	*mM*	0.100
$q_{O_{2}}^{min}$	*mM*/*h*	120.00
$q_{O_{2}}^{max}$	*mM*/*h*	306.66

#### ATP as energy currency of the cell

As glucose or oxygen-dependent thresholds for cell cycle progression or cell death led to results disagreeing with the experimental observations, in a next step we investigated the potential impact of glucose and oxygen on cell division and death. Besides being utilized as cell material, glucose is mainly used to gain energy for cell maintenance and reproduction. This happens by oxidation of glucose, which under standard conditions reads [54]:

$$\begin{array}{c} C_{6}H_{12}O_{6}+6O_{2}\to 6CO_{2}+6H_{2}O. \end{array}$$

The freed energy (Δ*G* = −2880*kJ*/*mol*) is thereby stored in ATP. We below denote in a highly simplified form by summarizing reactions the contributions of glucose and oxygen to the ATP production. For one mol of glucose, within Glycolysis,

$$\begin{array}{c} Glucose+2NAD^{+}\overset{k_{1}}{\to }2Pyruvate+2ATP+2NADH. \end{array}$$ (24)

Under anaerobic conditions (lack of oxygen), pyruvate is metabolized into lactate in order to convert NADH back to NAD^+^ (see Eq 29).

Otherwise, if sufficient oxygen is available, the two moles of NADH are turned into 4–6 ATP molecules by oxidadive phosphorylation, depending on the shuttle system transporting the electrons of the NADH through the mitochondrial membrane from the cytosol into the mitochondria (the inner mitochondrial membrane is impermeable for NADH or NAD^+^). For glycerin-3-phosphat-shuttle it is 4 ATP. While glycolysis occurs in the cytosol, the citric acid cycle occurs in the mitochondrial matrix, and oxidative phosphorylisation at the inner mitochondical membrane. In the presence of sufficient oxygen, under aerobic conditions, one mol of pyruvate is transformed to one mol of acetyl-CoA, and introduced into the citric acid cycle whereby one mol of NADH is generated. In the citric acid cycle, from one mol of acetyl-CoA, 3 moles of NADH, one mole of FADH_2_, and one GTP are generated. By oxidative phosphorylation, one mol of NADH yields 3 moles of ATP, and one mol of FADH_2_ yield 2 moles of ATP. For each mol of NADH or FADH_2_ entering oxidative phosphorylation, 1/2 mol of O_2_ is consumed. Summarizing, in the presence of sufficient oxygen, for one mol pyruvate, entering the citric acid cycle, 4 moles of NADH yielding 12 moles of ATP, 1 mol of FADH_2_ yielding 2 moles of ATP, and 1 mol GTP energetically corresponding to one mol of ATP are produced. As NADH from glycolysis (see Eq 24) is only turned to ATP in presence of sufficient oxygen, we add the emerging 2–3 ATP per NADH to the r.h.s. and the NADH to the l.h.s. of the aerobe pathway reaction:

$$\begin{array}{c} Pyruvate+NADH+3O_{2}\overset{k_{2}}{\to }17\left(18\right)ATP+NAD^{+}. \end{array}$$

As mentioned above, the reactions take partially place in the cytosol, partially in the mitochondria, and the ATP yield from NADH depends on the shuttle system used which is why the numbers of ATP yield can slightly differ. We here continue our calculation with 17 moles ATP per mol pyruvate.

We assume that the changes in intra-cellular glucose and oxygen concentrations mainly depend on the metabolic rates, *k*_1_ and *k*_2_, and the uptake rates from the extra-cellular space, *q*_*G*_ and *q*_*O*_2__,

$$\begin{array}{cc} \frac{d[G]}{dt} & =-k_{1}\left[G\right]+q_{G}, \end{array}$$ (25)

$$\begin{array}{cc} \frac{d[O_{2}]}{dt} & =-3k_{2}\left[Pyruvate\right]\left[NADH\right]{\left[O_{2}\right]}^{3}+q_{O_{2}}. \end{array}$$ (26)

The changes in ATP concentration can be written as following:

$$\begin{array}{c} \frac{d[ATP]}{dt}=2k_{1}\left[G\right]+17k_{2}\left[Pyruvate\right]\left[NADH\right]{\left[O_{2}\right]}^{3}. \end{array}$$ (27)

Assuming the equilibrium conditions of Eqs 25 and 26 are reached very fast we can reformulate Eq 27, and in case of [*G*] > [*G*]^*crit*^ obtain

$$\begin{array}{c} \frac{d[ATP]}{dt}=2q_{G}+\frac{17}{3}q_{O_{2}}=p_{ATP}. \end{array}$$ (28)

In case [*G*] ≤ [*G*]^*crit*^, the concentration of pyruvate becomes zero, hence no ATP is produced. To avoid that cells completely deprived of glucose are still capable of producing ATP, we assume the critical amount of glucose to be [*G*]^*crit*^ = 0.01*mM*.

Fig 8 illustrates how cells regulate their consumption of glucose and oxygen under very different circumstances, hyponutrition (low glucose) and hypoxia (low oxygen), in such a way that a yield of ATP is maintained at values between 1000 and 1700 ×10^−17^ *mM*/*h* per cell. This observation suggests that continuously dividing EMT6/Ro cells need at least 1000*mM*/*h*. On the other hand Eq 28 implies that EMT6/Ro cells metabolize only 10% of the consumed glucose in the Krebs-cycle under sufficient glucose and oxygen supply (see Fig 8). The other 90% are either fermented to lactate or used to build up other cell components (e.g. amino acids, nucleotides) nevertheless important for cell reproduction. Jiang et al. [22] indicated a much lower fraction (75%) of anaerobically metabolized glucose.

#### Lactate—the side product of anaerobe metabolism

Under anaerobic conditions the cell accumulates pyruvate molecules generated in glycolysis as these cannot be further processed in the citric acid cycle (or Krebbs cycle) without the presence of oxygen. On the other hand, *NADH* cannot be oxidized back to *NAD*^+^, which is needed for the glycolysis to continue. To over come this shortcoming of *NAD*^+^, pyruvate is reduced to lactate by incorporating a proton of *NADH*.

$$\begin{array}{c} Pyruvate+NADH\overset{k_{3}}{\to }Lactate+NAD^{+}. \end{array}$$ (29)

This process is called the lactic acid fermentation.

In the model we assumed lactate to be a direct side product of glucose metabolized anaerobically i.e., in absence of oxygen. For each molecule of glucose entering the anaerobic lactate fermentation the cell will produce 2 molecules of lactate. Assuming the lactate production rate to be *p*_*L*_ ≈ 2*q*_*G*_ (where *q*_*G*_ corresponds to anaerobic rate of glucose metabolisation) and lactate to leak out of cells and diffuse in the tissue, results in the following formulation of the lactate dynamics

$$\begin{array}{c} \frac{\partial [L]}{\partial t}=\nabla \cdot \left(D_{L}\left(\sigma \right)\nabla \left[L\right]\right)+2\left(q_{G}-\frac{q_{O_{2}}}{6}\right), \end{array}$$ (30)

where *D*_*L*_ is the diffusion coefficient (see Table S1 in S1 Document), *q*_*G*_ the glucose consumption rate (Eq 21) and *q*_*O*_2__ the oxygen consumption rate (Eq 22). σ is the local cell density.

#### Extra-cellular matrix

Components of the ECM are produced by resident cells, and secreted into the ECM via exocytosis [81]. Once secreted, they aggregate with the existing matrix. In the model ECM is produced by cells, diffuses within the interstitial space and degrades with a constant rate

$$\begin{array}{c} \frac{\partial [ECM]}{\partial t}=D_{ECM}\Delta \left[ECM\right]+k_{gen}^{ECM}\sigma -k_{deg}^{ECM}\left[ECM\right], \end{array}$$ (31)

where is the density of viable cells, [*ECM*] is dimensionless and proportional to the ECM concentration divided by a reference concentration which in Col IV stained micrographs correspond to the highest color intensity. Thus [*ECM*] ranges from 0 to 1 permitting a direct comparison with the image data.

#### Waste material

Cells dying either by apoptosis or necrosis are finally lysed and release their intra-cellular material into the interstitial space. We assumed some of these molecules inhibiting for cell cycle progression of other cells. The dynamics was modeled by a reaction diffusion equation:

$$\begin{array}{c} \frac{\partial [W]}{\partial t}=D_{W}\Delta \left[W\right]+k_{gen}^{W}\sigma_{D}-k_{upt}^{W}\sigma \left[W\right], \end{array}$$ (32)

where *σ*_*D*_ is the local density of dead cells (no matter if by necrosis or apoptosis) but not yet lysed cells, [*W*] is the density of waste, *D*_*W*_ is the waste molecule diffusion constant, $k_{gen}^{W}$ is the rate at which waste molecules leak into the interstitial space, $k_{put}^{W}$ is the rate at which waste molecules are taken up by viable cells.

### Temporal Evolution & Numerical Treatment

The cellular dynamics was modeled by a master equation describing the time evolution of the probability of the multi-cellular configuration. The molecular dynamics was modeled by a system of deterministic partial differential equations.

#### Cell kinetic: Gillespie algorithm

The master equation is solved numerically by the Stochastic Simulation Algorithm proposed in [82]. The idea is to investigate the expected time interval until the next event (cell migration, cell cycle progression, necrosis, apoptosis, division, …) and then choose an event randomly, where the probability for each choice reflects its relative weight compared to all possible configuration changes. A detailed description of this part can be found in [83].

#### Molecular kinetic: Steady state solution

As the temporal scale of the cellular dynamics (> hours) and molecular dynamics (< minutes) differ by several orders of magnitude, we could assume that the molecular concentrations reached their steady state very quickly after a change in the cellular configuration. Consequently, we updated the molecular gradients with a constant time step of 0.1h by solving the system of PDEs (Eqs 19, 28, 30, 31 and 32) for their steady state solution. We verified that choosing a smaller time step did not have any significant impact on the model kinetics, and by solving the full time dependent equation for selected parameter combinations, that the time scale at which the concentrations reached their steady state values were short compared to the time scale of cell growth, division and death considered here. To calculate the steady state solutions all the PDEs were solved by a finite difference scheme, with a central difference discretization of the diffusion operator, on the regular lattice used to construct the unstructured lattice populated by the cells (see S1 Document). The coupled equations for glucose and oxygen were solved monolithically, with a semi-implicit iterative scheme. The other PDEs were solved with the same spatial discretization. BiCGSTAB was used to solve all the linear problems.

## Supporting Information

S1 Document Details about the tumor growth model, model developement, additional simulation results and additional information on the image analysis. (PDF)
