# Supplementary material for: Inferring Growth Control Mechanisms in Growing Multi-cellular Spheroids of NSCLC Cells from Spatial-Temporal Image Data
Source: PLoS Comput Biol. 2016 Feb 11;12(2):e1004412. doi: 10.1371/journal.pcbi.1004412 (PMC4750943; doi:10.1371/journal.pcbi.1004412)
Supplement: S1 Document — (PDF) [file pcbi.1004412.s001.pdf]

Supporting Information - Model Details:

Details of numerical simulations, cell processes and parameters of the tumor growth model

In this work a three-dimensional (3D) multi-scale model for tumor growth is developed, where each cell is considered as an individual agent ([1], [2], [3] and [4]) and all molecular key players are represented as continuous function of concentrations in time and space. Parts of the model description are adaptations from [4].

Spatial Discretization

For numerical simulations of the cells a three dimensional Voronoi tessellation is implemented, where each lattice point can host only one cell at any time (see Fig. S1). The construction points of the Voronoi tessellation are uniformly distributed among the cubes of a square lattice with lattice constant a. One point in that tessellation is randomly placed into each cube. Constant a is chosen such that the average volume of a Voronoi cell, a^3, corresponds to that of a cell, $V = \pi/6d^3$. The cell diameter was found to be $d = 16.8\mu m$ in this work. I.e. $a = \sqrt[3]{V}$. The domain of numerical simulation is divided into $100x100x100$ lattice points, which corresponds to a total volume of about $2.4mm^3$. For the numerical simulations of the molecular dynamics the initial square lattice is used as spatial discretization. We assume that the molecular concentrations are piecewise constant among the cubes.

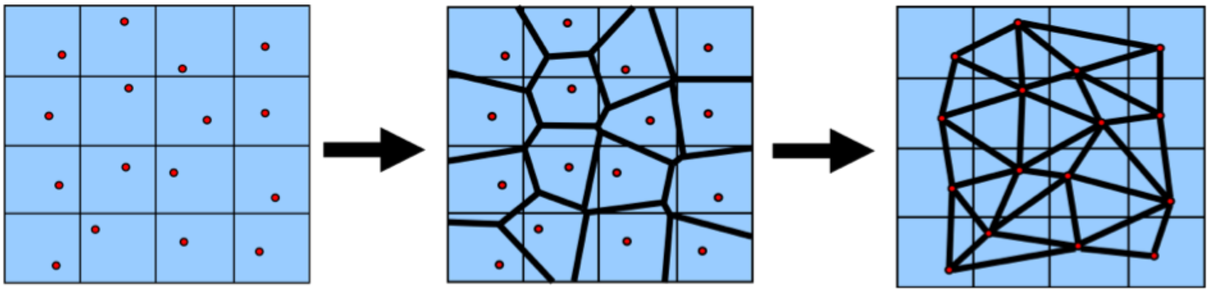

Figure S1: Spatial discretization. Schematic representation of the lattice topologies.

Actually, the selected modeling framework permits simulations to be scaled up to cubic centimeter sizes, though at the expense of lower spatial and functional resolution. Alternatively, hybrid models might be used, zooming in at the cell scale in regions of interest. However, we have chosen a CA model representing each cell individually to allow for cell heterogeneity at the cellular scale.

Cell Processes

The cell processes considered in this model are summarized as follows:

Cell Division: A Poisson process implies exponentially distributed waiting times. On the other hand, a chain of m consecutive Poisson processes leads to an Erlang-distributed waiting time of the whole chain. Here m determines the sharpness of the distribution around average waiting time. In the model the cell cycle is modeled as such a chain of m_d sub-processes. Beginning in cycle step $i = 1$, a

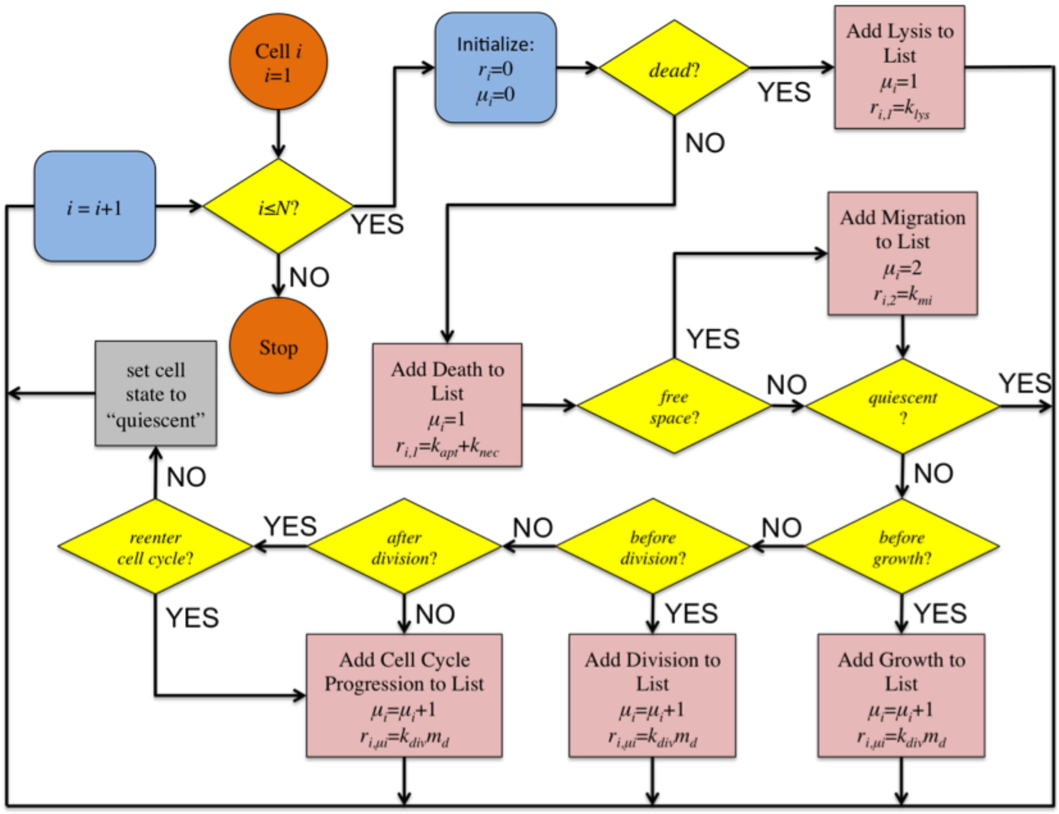

Figure S2: Flowchart of all accessible states, processes and rates. Schematic representation of how the rates of all the biological processes a cell can perform in its actual state/condition are set iteratively for all cells.

cell progresses in the cell cycle from i to $i+1$ with rate $m_d k_{div}$ with k_{div} being the division rate. When $i = m_g$ the cell expands to a random free neighbor site. If other cells already occupy all neighboring lattice sites then one neighboring site will be freed first by pushing adjacent cells along the shortest path toward the closest free lattice. When $i = m_d$ then the cell divides into two daughter cells of which one keeps occupying the mothers lattice site and the second a randomly chosen free neighbour site. Then both cells decide with probability p_{div} whether to reenter the cell cycle, setting $i = 1$, or to become quiescent.

Cell Quiescence: Once quiescent, a cell can reenter the cell cycle with rate $p_{re}k_{re}$.

Migration: In our model, cells are able to move to a randomly chosen free neighbor site with same (hopping) rate kmig (see Table S1 and Figure S1). This migration rate k_{mig} can be obtained from the relation $k_{mig} = D/l^2$, where D is the diffusion coefficient of the cell and l the hopping distance (= cell diameter) [5]. The diffusion coefficient depends on the properties of the cell and the surrounding (liquid) media $D = k_BT/(6\pi\eta R_0)$, where $k_B(= 1.38 \cdot 10^{-23}JK^{-1})$ is the Boltzmann constant, T (= 310.15K) is the temperature, η (= $700\mu Nsm^{-2}$ for H_2O at 37°C) and R_0 is the hydrodynamic radius of the cell which can be assumed to be approximately the cell radius, R0 = Rcell. For an average cell diameter of $20\mu m$ and a medium viscosity close to that of water k_{mig} will thus be about 1.75 h^{-1} (approximately 1 cell diameter in 105 minutes). Similar estimates have been independently derived in [6] and references therein.

Apoptosis (programmed cell death): All cells can undergo apoptosis, a programmed cell death (see Figure S2). When this occurs, cells activate the apoptotic pathway, which will lead to cell shrinkage, nuclear fragmentation, chromatin condensation, chromosomal DNA fragmentation and cell fragmentation into apoptotic bodies. The corresponding process is accounted for in the model by changing from a proliferating or quiescent stage to a dead stage at a rate k_{apt} (see Table S1). Within the simulated sensitivity analysis we varied the apoptosis rate and found that only very small apoptosis rates were in agreement with the dead cell profile in the data, so that we finally neglected apoptosis.

Necrosis: If dying cells are not able to initiate the apoptotic pathways due to cellular injury, intoxication or a dysfunctional apoptotic pathway as in the case of many cancer cell lines they will undergo necrosis. In the model cells, which are deprived by nutriments and exposed to lactate, change from a proliferating or quiescent stage to a dead stage at a rate k_{nec} (see Table S1).

Lysis: Disposal of cellular debris resulting from apoptosis or necrosis is carried out by a lysis process [7], for which a lysis rate $k_{lys} = 0.035h^{-1}$ (about 30h) will be assumed (see Figure S2 and Table S1). This is about 10 times slower than phagocytosis (digestion of cellular debris by macrophages) observed in vivo ([8]), but within the range reported for in vitro cultures (0.002 h^{-1} for Fibrobacter succinogenes [9]; 0.07 h^{-1} for VO 208 Hybridoma cell line [10]). It should be noted in this context that tumor growth within the size limits considered in this work is closer to in vitro cultures than to in vivo situations. Lysis is mimicked in our model by means of a Poisson process, which removes dead cells from the lattice.

Time Evolution of the System

We implement a version of the Gillespie algorithm [14], adapted to the cell population system considered in this work. To this end, some explanations are in order. We shall index by μ a class of a process that cells can perform, say proliferation, migration, apoptosis or necrosis and lysis. The related process rate, denoted by r_μ does not need to be constant for all cells. For example, proliferating rates depend on the cell lines considered. Therefore, a process $P_{x,\mu}$ is specified by its process class μ, but

In the following Table S1 the numerical simulations parameters are listed:

	Parameter	Symbol	(Range*) Value	Unit	Source*
**Domain**	Lattice size (nb. lattice sites)	$h \times w \times d$	$100 \times 100 \times 100$		assumed
	Lattice constant	a	13.44	μm	estimated
**Initial**	Initial spheroid radius	R_{init}	175	μm	fitted
**Condition**	Initial fraction of quiescent cells	p_{qui}^{init}	(0...0.99) 0.75		fitted
**Cell**	Migration rate	k_{mig}	$1.75 \times a$	h^{-1}	estimated
**Processes**	Apoptosis rate	k_{apt}	0.000417	h^{-1}	assumed
	Necrosis rate	k_{nec}^{max}	(0...0.1) 0.01	h^{-1}	fitted
	Lysis rate	k_{lys}	(0...0.1) 0.035	h^{-1}	fitted
	Division rate	k_{div}^{max}	0.032	h^{-1}	estimated
	Division depth	ΔL	130	μm	estimated
	Cell cycle steps until growth, division	m_g, m_d	2, 10		assumed
**Molecular**	Oxygen diffusion coefficient**	D_O	6300000	$\mu m^2 h^{-1}$	[11]
**Kinetics**	Glucose diffusion coefficient**	D_G	378000	$\mu m^2 h^{-1}$	[11]
	Lactate diffusion coefficient**	D_L	756000	$\mu m^2 h^{-1}$	[12]
	ECM diffusion coefficient**	D_W	0	$\mu m^2 h^{-1}$	assumed
	Waste diffusion coefficient**	D_W	$(10...10^6)$ 100000	$\mu m^2 h^{-1}$	fitted
	ECM production	k_{gen}^{ECM}	(0...0.1) 0.0005	h^{-1}	fitted
	ECM degeneration	k_{deg}^{ECM}	(0...0.1) 0.0033	h^{-1}	fitted
	Waste production	k_{gen}^{W}	(0...100) 10	h^{-1}	fitted
	Waste degeneration	k_{deg}^{W}	(0...100) 0	h^{-1}	fitted
**Coupling Scales**	critical product of glucose and oxygen concentration	p^{oxgluc}	0.025	mM^2	[11]
	critical ATP-production rate	p_{ATP}^{min}	900	mMh^{-1}	estimated
	Critical waste conc.	$[W]^{max}$	(0...1) 0.008	mM	fitted
	Critical oxygen conc.	$[O]^{min}$	(0...1) 0.07	mM	fitted
	cell cycles under waste exposure and oxygen deprivation	n_{exp}^{max}	(4...8) 8		fitted
	critical lactate concentration	$[L]^{max}$	20	mM	fitted, [13]
	Lactate hill coefficient	n	(1...3) 2		fitted
	critical ECM concentration	$[ECM]^{min}$	(0...0.1) 0.003		fitted

* Those parameters not found in the literature were either *estimated* or *fitted* from the experimental data presented in this article or in referenced work. The impact of choices on the resulting effects was subsequently analyzed in sensitivity analysis (within ranges indicated). ** The diffusion coefficients in medium were assumed to be 30 times larger compared to the cellular phase of the spheroids as indicated in this table.

Table S1: Model parameters used in numerical simulations.

also by the cell x where it takes place. For instance if μ represents proliferation, the corresponding replication process can be summarized as follows:

$$P_{x,\mu} : x \to 2x, \tag{S1}$$

where 2x represents the two daughters arising from x. The algorithm describing the temporal evolution of the system is shortly described in the provided pseudo code. Lines within braces at each step refer to the procedure sketched at the end of the description.

Step 0 (lines 1 to 8 - Initialization): The tumor spheroid is initialized in the center of the domain. Within a sphere of radius R_{init} cells, where a fraction of p_{qui}^{init} is quiescent from the beginning, occupies all lattice sites. Then the list of all possible processes at this state of the system P is set

accordingly. Set the time variable t to zero and initialize the unit-interval uniform random number generator (URN).

Steps 1 to 4 (lines 9 to 34): A step-by-step time evolution is processed within a loop. The following steps 2 to 5 are repeated until the system reaches the end time t_{max}, when there are no more processes to execute (for instance all cells are dead) or the maximum number of cells considered is reached ($N_{cells}^{max} = 10^6$ cells).

Step 1 (line 11 - Total Transition Rate): Calculate and store as r_Σ the sum of rates $r_{x,\nu}$ of all processes $P_{x,\nu} \in P$.

Step 2 (lines 13 and 14 - Time Increment): Generate a random number $u1$ using the unit-interval uniform random number generator (URN), and calculate $\tau = -ln(u1)/r_\Sigma$.

Step 3 (lines 15 to 24 - Process Selection): The process $P_{y,\mu}$ to perform during this iteration is chosen randomly from the list of all processes P taking into account that the probability of each process $P_{x,\nu} \in P$ to be chosen is proportional to its rate $r_{x,\nu}$. As proposed in (Gillespie, 1977) this can be done as follows: generate a second random number $u2$ using the unit-interval uniform random number generator. Then step-by-step sum up as r'_Σ the rates $r_{x,\nu}$ of all processes $P_{x,\nu} \in P$ until the condition $r'_\Sigma - r_{x,\nu} < u2 \cdot r_\Sigma \leq r'_\Sigma$ is satisfied. Then store $P_{x,\nu}$ as the selected process in $P_{y,\mu}$.

Step 4 (lines 25 and 26 - Update cellular system): Adjust the cell set to account for what has happened during process $P_{y,\mu}$ (e.g. remove cells, add cells, move cells or change environment of a cell). Update the list of possible processes according to the new cell set (e.g. add/remove processes after an environmental change).

Step 5 (lines 27 to 31 - Update molecular concentrations): For a fix time step dt the molecular concentrations are updated to the steady state solutions of the respective equations 19, 28, 29 and 30.

Step 6 (lines 32 to 3 - Update time): Increase t by τ.

Pseudo Code Guidelines

Algorithm 1 Stochastic multiscale simulation algorithm

{***Input parameters:***}
t_{max} := maximum duration of numerical simulation
dt := time step for updating molecular concentrations
$[G]^{med}$, $[O]^{med}$:= medium concentrations of glucose and oxygen
{***Initialization:***}
Input the initial set of cells $C := \{x | x$ is a cell within the volume $V\}$
Determine the process array $P := \{P_{x,\nu}|$ if $x \in X$ and x is reactant of $\nu\}$
Set $t := 0$
repeat
    {***Total transition rate:***}
    Calculate $r_\Sigma := \sum_{P_{x,\nu} \in P} r_{x,\nu}$.
    {***Time step:***}
    Generate $u1$ from URN
    Take $\tau := -ln(u1)/r_\Sigma$
    {***Select random process regarding its probability:***}
    Generate $u1$ from URN
    Set $r'_\Sigma := 0$
    for all $P_{x,\nu} \in P$ **do**
        if $r'_\Sigma - r_{x,\nu} < u2 \cdot r_\Sigma \leq r'_\Sigma$ **then**
            $y := x$
            $\mu := \nu$
        end if
        Put $r'_\Sigma := r'_\Sigma + r_{x,\nu}$
    end for
    {***Execute process:***}
    Adjust C and P according to $P_{y,\mu}$
    {***Update molecular concentrations:***}
    if $\exists i \in \mathbb{N} : t < i \cdot dt \leq t + \tau$ **then**
        Solve for steady state solutions of eqns. (19), (28), (29) and (30)
        Update $u \in \{G, O_2, L, ECM, W\}$ such that $\frac{\partial u}{\partial t} = 0$
    end if
    {***Update time:***}
    Put $t := t + \tau$
until $t \geq t_{max}$ **or** $P = \emptyset$ **or** $|C| \geq N_{cells}^{max}$

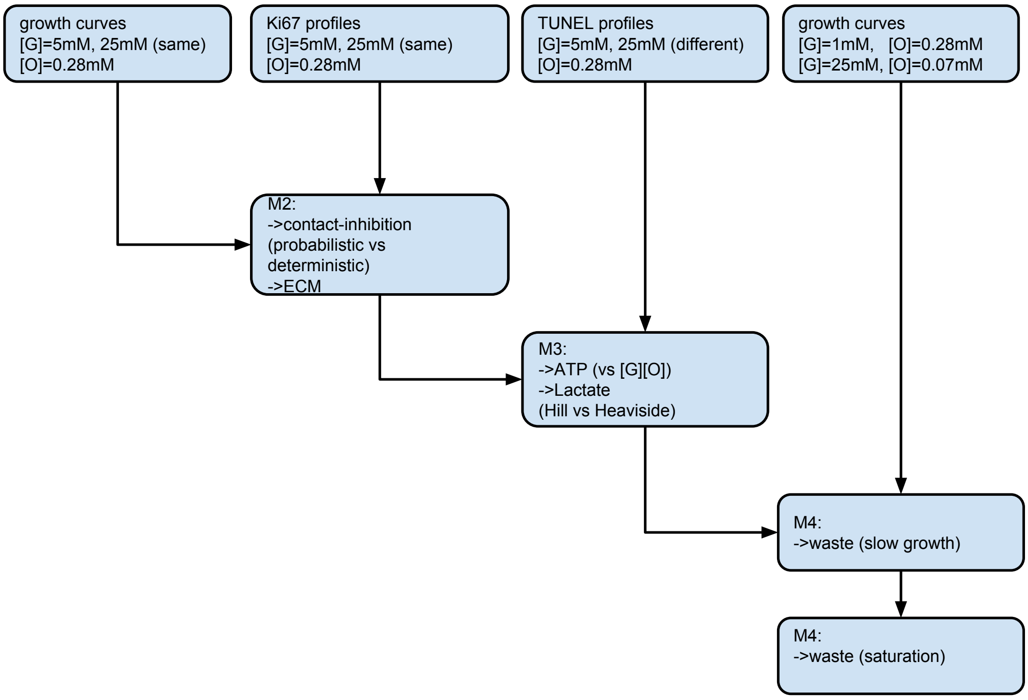

Figure S3: The figure illustrates the stepwise development of the final model 4 (M4) in the paper by stepwise including further data sets and adding further mechanisms (going from M1 to M2, M2 to M3, M3 to M4) until the final model M4 has been accomplished.

Supporting Information - Alternative Model Mechanisms & Parameters Sensitivity

In this section we present some sensitivity analysis as well as alternative mechanisms which are not mentioned, but largely guided our decisions toward the final models presented in the main text. The core part of the propose hybrid model are the transitions between internal cell states (cell cycle, G_0 and death) depicted in figure 5. Here the model outcome largely depends on the respective transition rates and their dependency on the cell arrangement and local molecular concentrations.

Cell Cycle & Quiescence

As described above and shown in figure 5, the decision whether a cell continues to proliferate or becomes quiescent is made at a check-point directly following cell division and depends on probability p_{div}. Once quiescent, the decision to reenter the cell cycle depends on p_{re}. On the other hand, k_{re} determines how fast cells reenter the cell cycle from G_0 under favorable conditions. Several examples are next shown on how the choice of both probabilities influences growth curves and radial profiles.

Hypothese I: Long-range contact inhibition controlling cell cycle progression

First, we assume that cell cycle progression and reentrance (from G_0) are controlled in a similar manner by extra-cellular matrix (ECM) and contact-inhibition

$$p_{div} = p_{div}^{max} H([ECM] - [ECM]^{min}) e^{-L/\Delta L} \tag{S2}$$

$$p_{re} = p_{re}^{max} H([ECM] - [ECM]^{min}) e^{-L/\Delta L}, \tag{S3}$$

where p_{div}^{max} and p_{re}^{max} are the base probabilities, L the cells distance to the closest free space, ΔL the respective reference distance, and $[ECM]^{min}$ the minimally required ECM.

Figure S4 shows, how varying k_{re}, or p_{re}^{max} (asymmetric scenario), or p_{div}^{max} and p_{re}^{max} simultaneously (symmetric scenario) effect the growth curves and radial distribution of proliferating cells. In the entirely symmetric case, i.e. the effective rates $p_{div}k_{div,m}$ and $p_{re}k_{re}$ are kept equal, the model is in good agreement with the experimental growth curves for low progression and reentrance probabilities. On the other hand, the fraction of proliferating cells is either underestimated at the outer border or underestimated at the center. In the asymmetric case, growth curves and radial profiles are in much better agreement between experiment and model, if either p_{re}^{max} or k_{re} are close to zero.

Hypothese II: Short-range contact inhibition controlling cell cycle reentrance

An alternative assumption is, that quiescent cells reenter the cell cycles only if they "sense" free space in their direct vicinity, for example via membrane sensors:

$$p_{re} = p_{re}^{max} H([ECM] - [ECM]^{min}) \cdot \begin{cases} e^{-L/\Delta L}, \text{if } \exists \text{ free site in direct vicinity} \\ 0, \text{ else.} \end{cases} \tag{S4}$$

Figure S5 shows that reentrance which can only be initiated if free space is accessible in direct vicinity, is more stable to different reentrance rates k_{re}. Here, the critical amount of ECM a cell needs to progress in the cell cycle has a much larger impact on the proliferating cell fraction. Modeling ECM, especially at the outer border, is essential to explain the low fraction of proliferation cells observed experimentally.

Symmetric case: Varying $p_{re}^{max} = p_{div}^{max}$, keeping $k_{re} = k_{div,m}$:

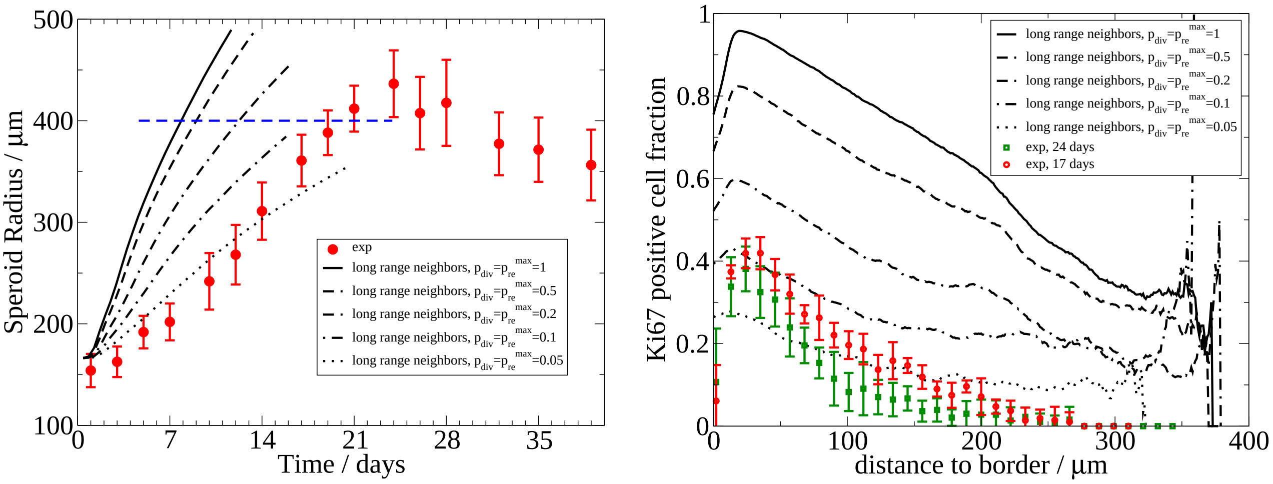

Asymmetric Case: Varying k_{re}, keeping $p_{re}^{max} = p_{div}^{max} = 1$:

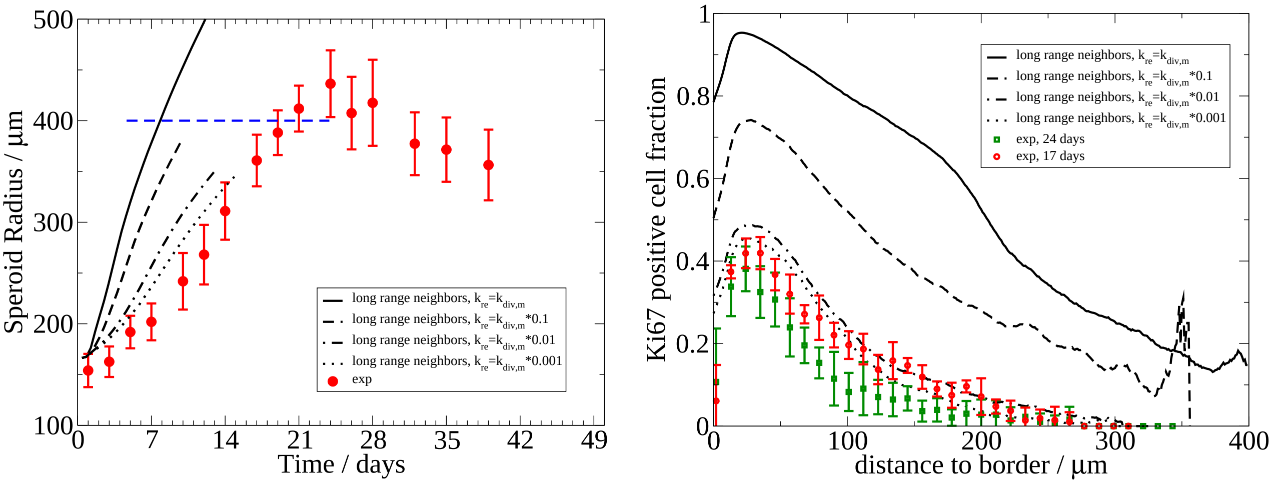

Asymmetric case: Varying p_{re}^{max}, keeping $p_{div}^{max} = 1$ and $k_{re} = k_{div,m}$:

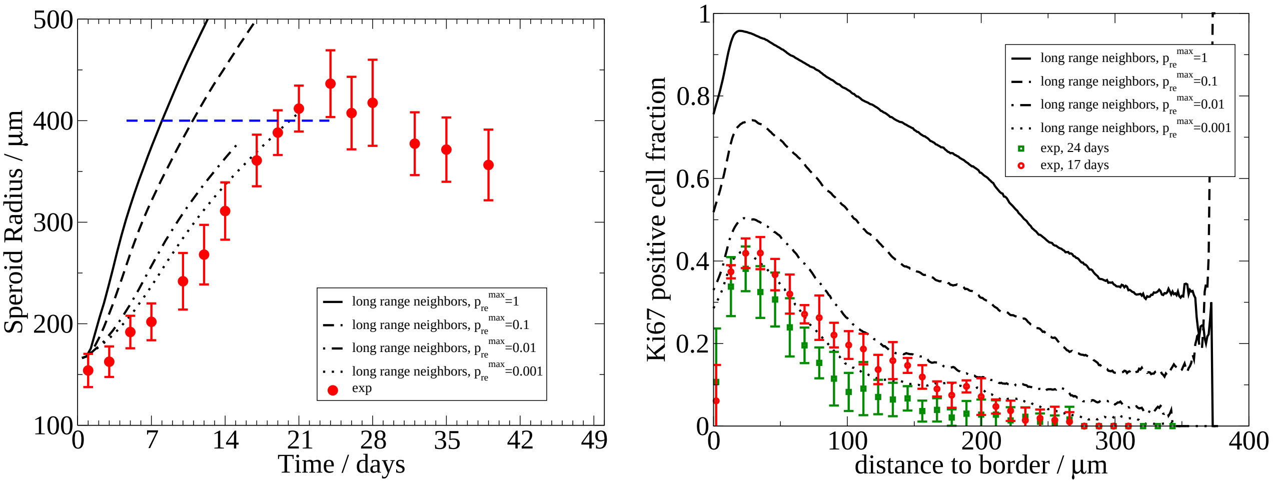

Figure S4: The reentrance mechanism is similar to the division: the probability to proceed in the cell cycle depends on available space in the extended neighborhood. Experimental (mean, standard deviation) growth curves (left) and radial profiles of Ki67-positive cells (right) are compared to model simulations (black lines). Notice that the solid black curves are precisely the same as $p_{re} = p_{div}$ and $k_{re} = k_{div}$. Moreover, by construction the curves in the bottom row become identical to those in the center row, if for the modifier $0 < \alpha \leq 1$ in the bottom row the setting $p_{re}^{max} = \alpha$ is chosen, and in the center row $k_{re} = k_{div,m}\alpha$ is chosen.

Vary k_{re}:

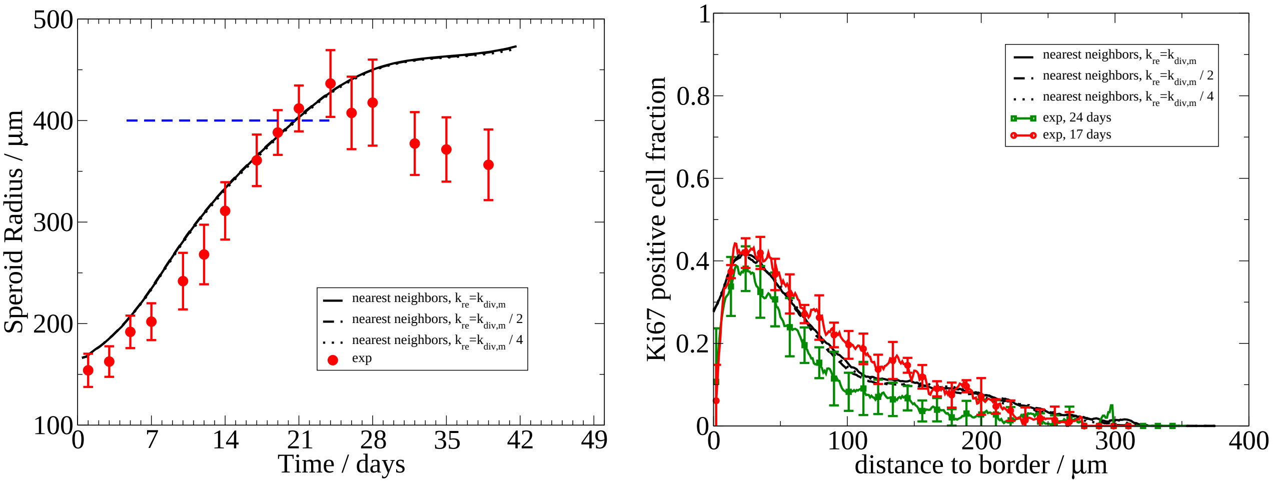

Vary $[ECM]^{min}$

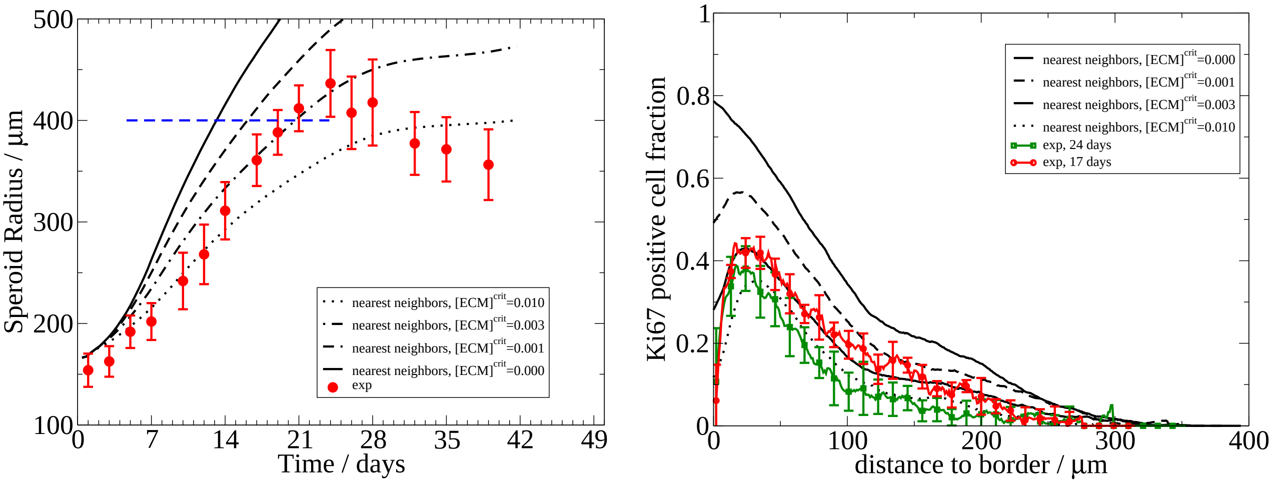

Figure S5: The reentrance mechanism is distinct from division: the probability to proceed in cell cycle depends on available space in direct neighborhood. Experimental (mean, standard deviation) growth curves (left) and radial profiles of Ki67-positive cells (right) are compared to model simulations (black lines). The model simulations were done for different reentrance rates k_{re} (top) and the ECM growth threshold $[EMC]^{min}$.

Hypothese III: cell cycle progression not controlled by contact-inhibition

Figure S6 shows simulation results with different combinations of mechanisms from model 4 for condition III and in each case with suppression of contact inhibition. The parameters have been chosen as in Fig. 10 (main text). Suppressing of contact inhibition leads to large deviations from experimental data, particularly for the KI67 profile. As long as quiescence due to waste exposure is not present, the KI67 profile remains stationary, otherwise it drops with time. So for the former one might speculate that a proper calibration of parameters may permit reproduction of the KI67 profile. As explained in the main text of the paper, our and previous findings in models and the data of Sutherland and co-workers (see text) suggest that nutriment - dependent proliferation control is not sufficient to explain the experimental findings: Firstly, between [G]=25mM and [G]=5mM the experimental proliferation pattern does almost not change while a difference in glucose medium concentration would cause a difference in the size of the proliferating rim if proliferation would depend on the glucose concentration [G]. A different size of the proliferating rim is basically not observed between [G]=25mM, 5mM (this was also the case in EMT6/Ro cells, see Freyer, Sutherland, 1986). However, if cell cycle entrance

were independent of [G], then [G]=1mM would not lead to a drop in proliferation. Still it would be an interesting future goal to fit all model parameters new when dropping contact inhibition to see how close to the data one could come, which as it is very time consuming, we would leave for the future, when automated fitting procedures for stochastic cell models in high dimensional parameter spaces are available.

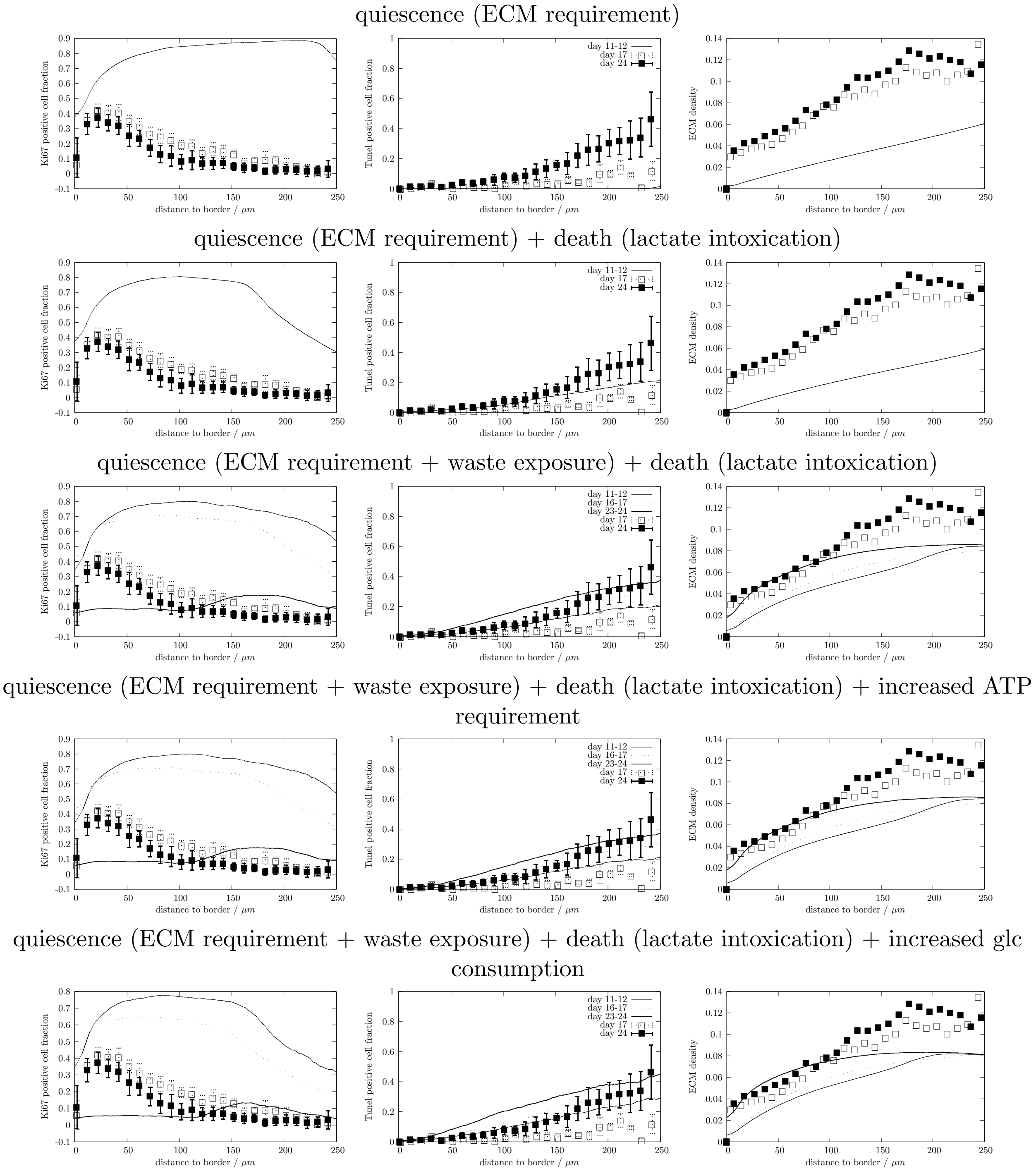

Figure S6: No contact-inhibition. The figures show a comparison between experimental measures (medium concentrations: $[G] = 25mM$, $[O] = 0.28mM$) with model predictions for different cell requirements for growth and survival. All simulations are based on model 4 (Tab. 6) assuming $\Delta L = \infty$. Involved parameters are the minimal ECM density $[ECM]^{min}$, maximal lactate concentration $[L]^{max}$, critical waste concentration $[W]^{max}$, critical ATP production rate p_{ATP}^{max} and glucose consumption rate q_G^{max}.

Choice of Death-Rate-Lactate-Dependency

Experiments show that cells exposed to larger lactate concentrations also have an increased death rate (e.g. ref.[15, 13]). Figure S7 shows measurements done by Ozturk et al. [13]. We tested how the radial distribution of necrotic cells changes if the relation between death rate and local lactate concentration is described by either a linear dependence, a Hill equation, or a Heaviside function.

$$linear : k_{nec} \sim [L]/[L]^{max} \tag{S5}$$

$$Hill : k_{nec} \sim \frac{[L]^n}{([L]^{max})^n + [L]^n} \tag{S6}$$

$$Heaviside : k_{nec} \sim H([L]^{max} - [L]) \tag{S7}$$

Very interestingly, we obtained the best agreement between model simulations and experimental findings for parameter values which closely resemble the lactate-dependent death rate found independently by Ozturk et al. [13] (Fig. S7). These authors observed a death rate increasing with lactate.

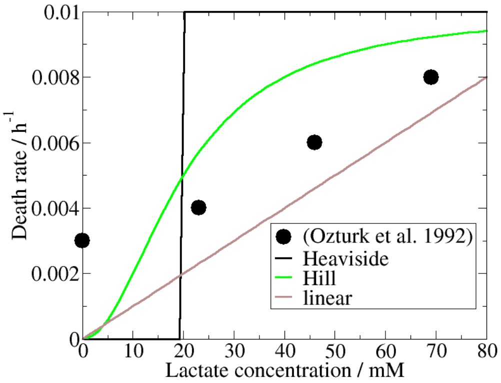

Figure S7: Relation between lactate concentration and death rate. Death rates measured by Ozturk et al. [13] for different lactate concentrations (dots) are compared to three theoretical descriptions: Heaviside, Hill and linear function.

Figure S8 shows a comparison of the three modeled kinetics for high glucose medium concentrations (25mM) with the corresponding experimental data. The growth curve is not affected by the choice of kinetics. On the other side, the necrotic profiles directly depend on it. While the Heaviside function (see figure S8(d)) leads to too sharp transition between viable and necrotic tissue regions, a linear dependency (see figure S8(a)) would lead to over-smoothed transitions. Only the Hill equation (see fig. S8(b)) could explain the radial profiles shape. For $25mM$ glucose and $0.28mM$ oxygen, oxygen becomes the limiting factor before glucose. Thus, initially the viable rim is much thicker as cells still have enough glucose to survive in anaerobe conditions. But over time, lactate accumulates as a direct side-product of anaerobic metabolism until it reaches toxic concentrations.

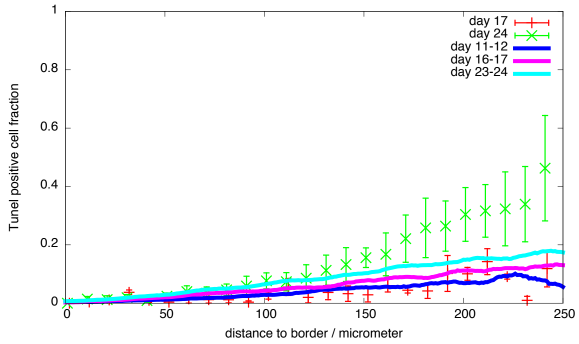

(a) Linear function

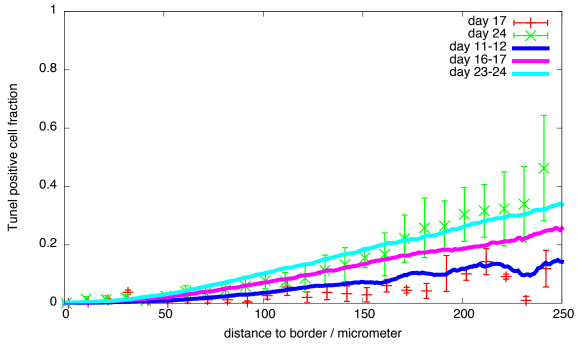

(b) Hill function

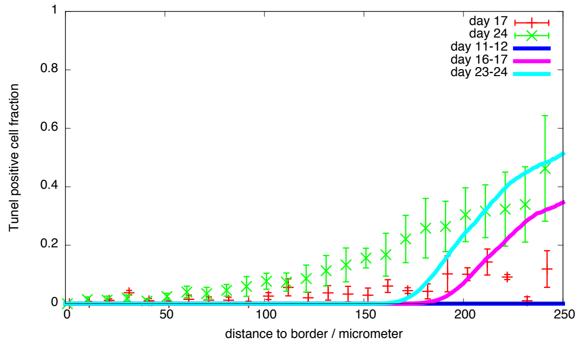

(c) Heaviside function

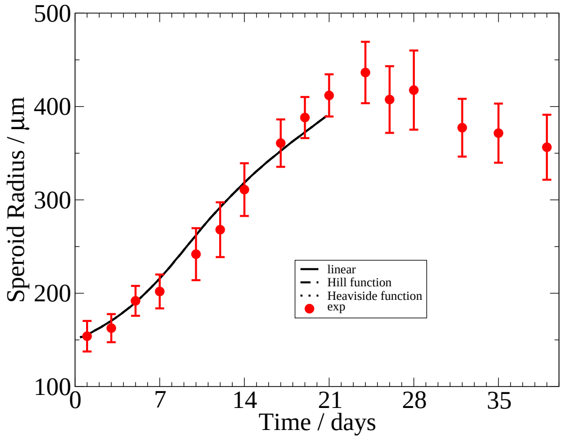

(d) growth curves (linear, Hill and Heaviside function)

Figure S8: Comparison of different lactate-death-kinetics with experimental data for medium concentrations $[O] = 0.28mM$ and $[G] = 25mM$. (a),(b),(d) show the radial profiles of the necrotic cell fraction at different time points separately for linear, Hill and Heaviside functions.

Cell migration and Necrotaxis

.

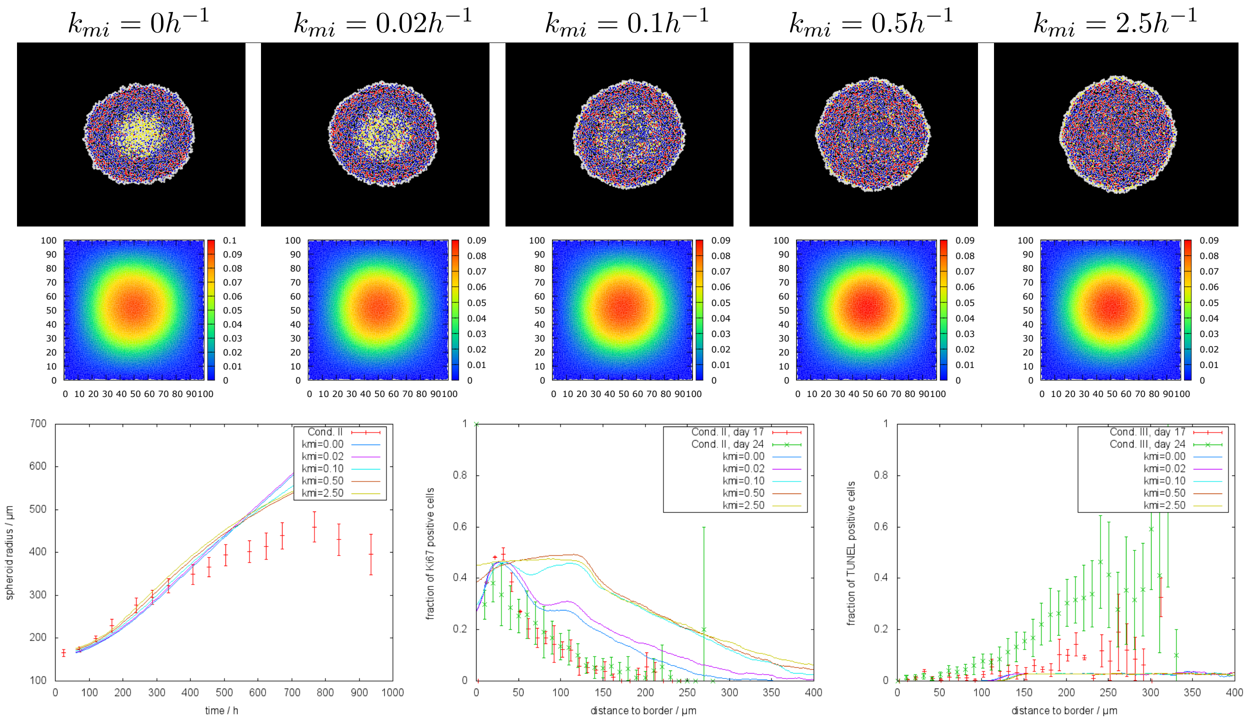

Figure S9: Condition II: $[G] = 5mM, [O_2] = 0.28mM$. top: The images show the distribution of Ki67-positive cells (red), Ki67-negative cells (blue) and necrotic debris (yellow) in a cross section of a simulated spheroid after 15days. Middle: In the same cross section the concentrations of waste is depicted by a color code ranging from blue=0 to red=0.1. The columns represent different hopping rates k_{mi} with which cells move up the waste gradient originating from necrotic cells. Bottom: the growth curves, proliferation profiles and necrotic profiles predicted by the different hopping rates are compared to experimental measurements for the same medium concentrations. For all migration rates, the necrotic cell fraction in the simulations is far below the experimentally observed profile.

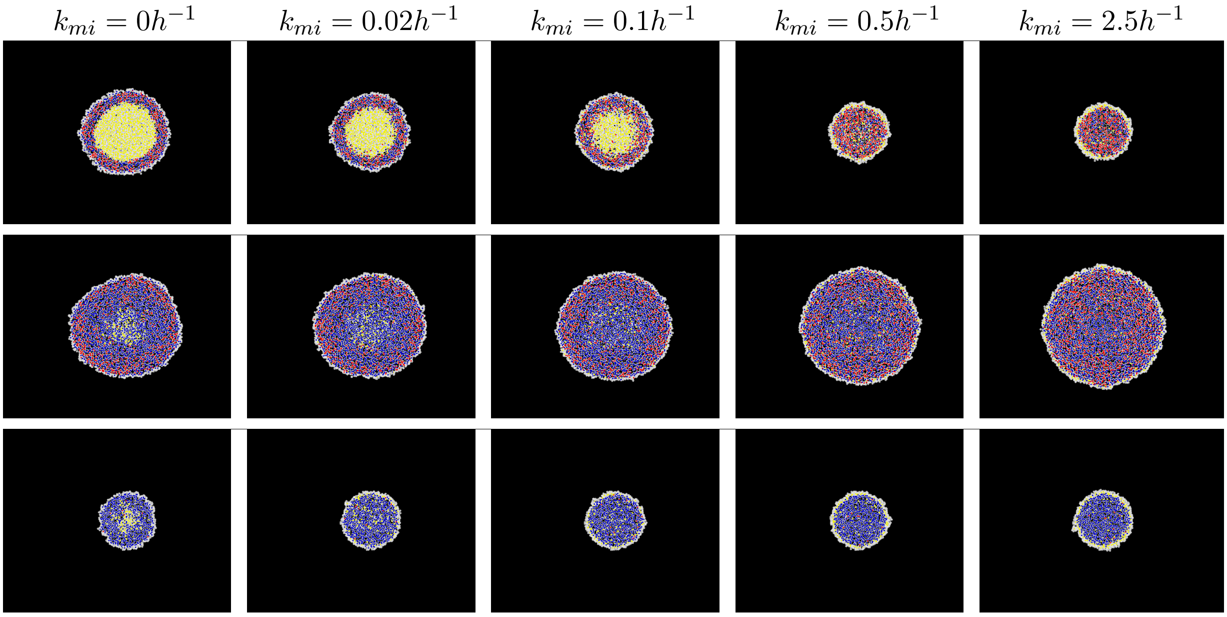

Figure S10: Condition I, III & IV. The images show the distribution of Ki67-positive cells (red), Ki67-negative cells (blue) and necrotic debris (yellow) in a cross section of a simulated spheroid after 15days. The columns represent different hopping rates k_{mi} with which cells move up the waste gradient originating from necrotic cells. The rows represent different nutriment concentrations in the surrounding growth medium (top: $[G] = 1mM, [O] = 0.28$; top: $[G] = 25mM, [O] = 0.28$; bottom: $[G] = 25mM, [O] = 0.07$).

Stochasticity & Fluctuations of Model Output

We tested how the model results vary starting from the same initial condition, but with different random seeds. Figure S11 shows the growth curves as well as the radial profiles of 10 single simulations compared to the mean and standard deviation of the corresponding experiments. We note that the fluctuations in the experimental data seems to be much higher than between independent simulations. Possible reasons may be heterogeneities in the experimental initial conditions as well as measurement errors which add up to the true underlying biological fluctuations.

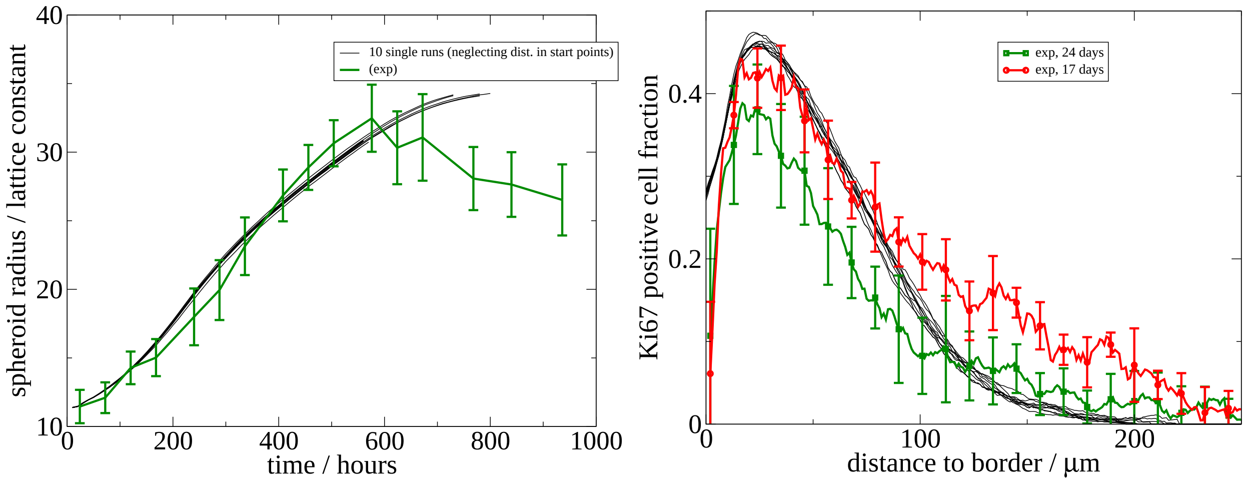

Figure S11: Stochastic fluctuations between individual simulations. The figures show 10 single model simulations (black lines) with identical initial conditions, but different random seeds. The corresponding experimental data is depicted by average and standard deviation (green/red).

Model Predictions

The following figure shows the temporal evolution of the radius as well as the radial profiles predicted for 4 nutriment conditions where no experimental data was available.

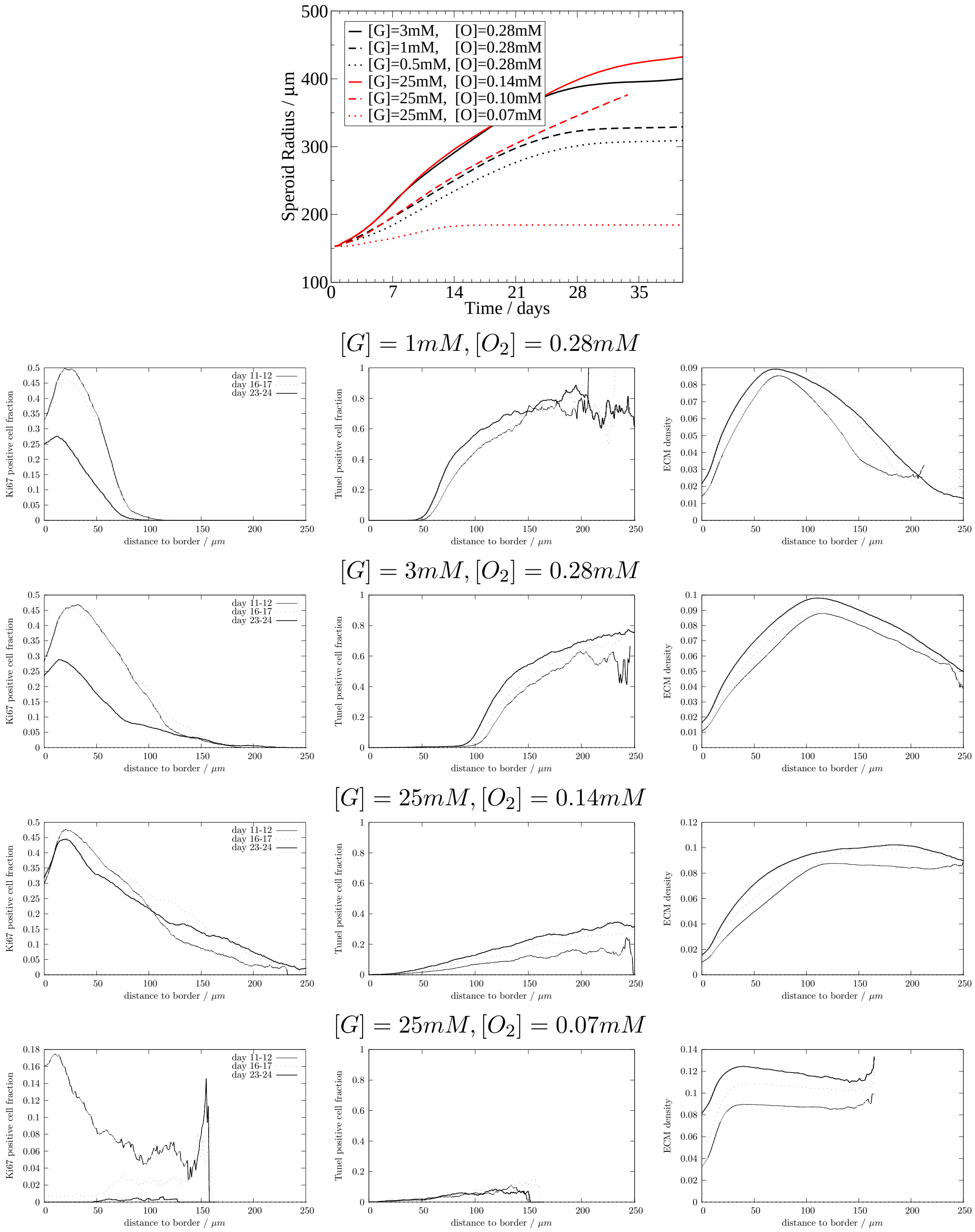

Figure S12: Model predictions. The curves shown here are model predictions for four different nutrient conditions where no histological data was available. They show the growth curves (top), the radial profiles of the proliferating cell fraction (left), the necrotic cell fraction (middle) and the extra-cellular matrix density (right) at three different time points (12, 17 and 24 days).

Figure S13 shows how the radial information is extracted from the micrographs, in this specify example from a Ki67-stained slice of a tumor spheroid grown in a well-nourished medium ($[G] = 25mM, [O_2] = 0.28mM$) and sacrificed after 24 days. First all nuclei and those stained positively with Ki67 are counted for different intervals, or *bins*, of distances to the outer spheroid border. The bin size is $1\mu m$. Then the values in both bins are divided by each other to get the fraction. Finally from the fractions of all the individual images one can calculate the mean and standard deviation over many micrographs of different spheroids.

As figure S13 shows, increasing bin size is not changing the overall shape of the radial fraction profile, but rather smoothes out some noisy spikes toward the spheroid centre which were mainly a result of lower cell counts in the central regions.

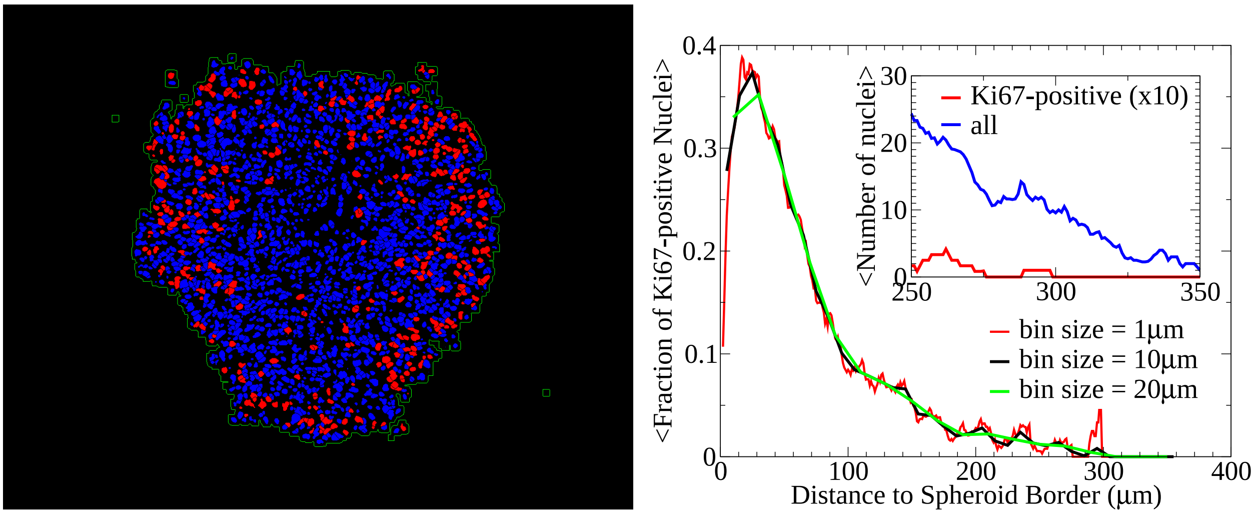

Figure S13: Analysis of segmented micrographs. *Left:* the image is the segmentation result of a Ki67-stained spheroid slice. The colors indicate the approximated spheroid border (green), Ki67-positive (red) and Ki67-negative (blue) nuclei. *Right:* the curves show the fractional cell count of Ki67-positive nuclei per total number of nuclei as function of the distance to the spheroid border. Here the different colors correspond to different bin sizes used to create the curves. The inset shows the raw cell count (bin size $= 1\mu m$) of Ki67-positive (red) and all nuclei (blue) in the region where the fraction of Ki67-positive nuclei is close to 0. All curves are averaged over 6 individual images. The Ki67-positive cell counts were multiplied by 10 for better visibility.

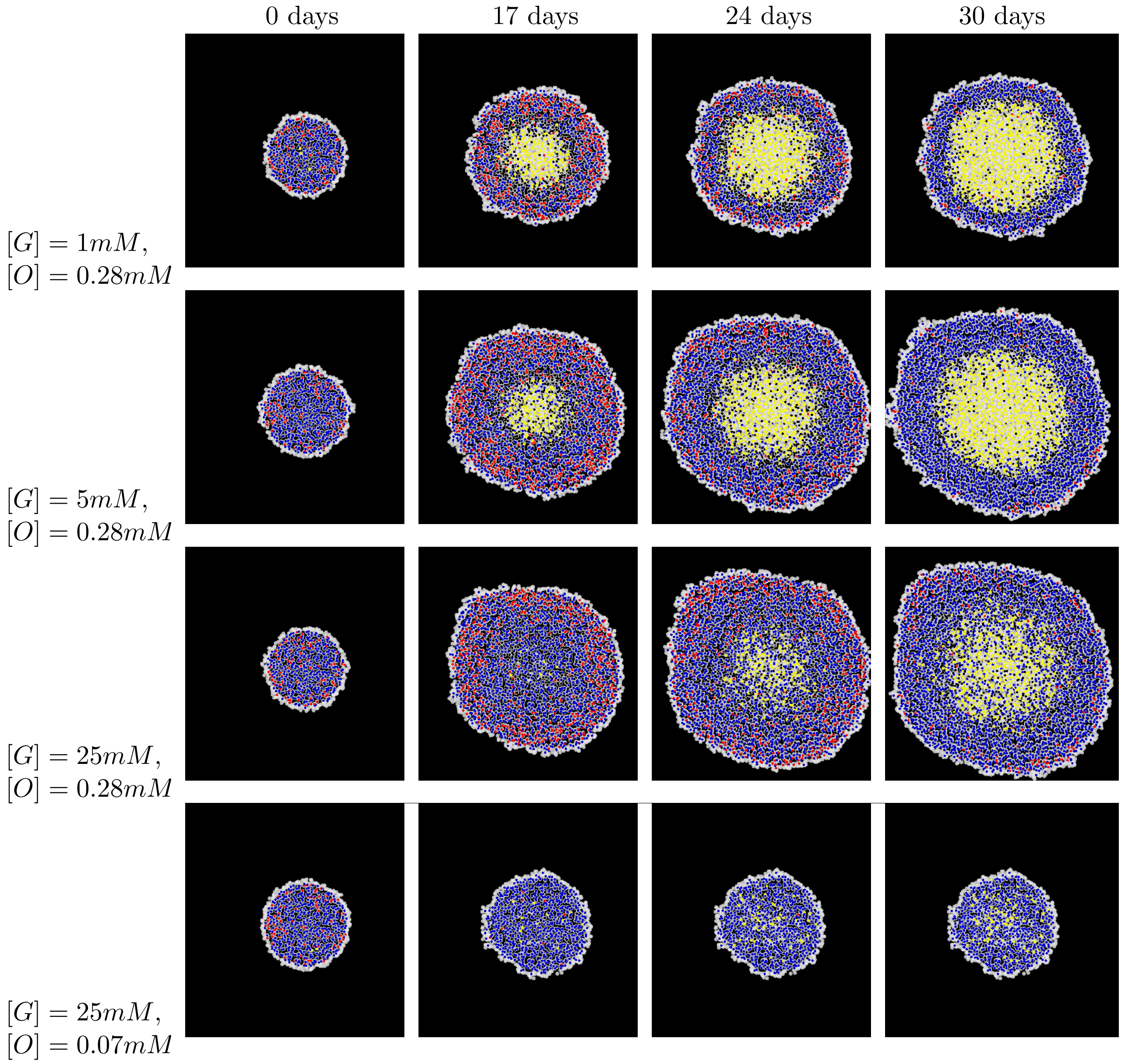

Figure S14: Simulation snapshots. The images show the distribution of Ki67-positive cells (red), Ki67-negative cells (blue), dying cells (black) and necrotic debris (yellow) in a cross section of a simulated spheroid after 0, 16, 24 and 30 days. The rows indicate the different medium concentrations of glucose and oxygen the simulated cell populations were exposed to.

References

[1] D. Drasdo and S. Hoehme, "A single-cell based model to tumor growth in-vitro: monolayers and spheroids," *J. Phys. Biol.*, vol. 2, pp. 133–147, 2005.

[2] M. Radszuweit, M. Block, J. G. Hengstler, E. Schöll, and D. Drasdo, "Comparing the growth kinetics of cell populations in two and three dimensions.," *Physical review. E, Statistical, nonlinear, and soft matter physics*, vol. 79, p. 051907, May 2009.

[3] M. Block, E. Schoell, and D. Drasdo, "Classifying the growth kinetics and surface dynamics in growing cell populations," *Phys. Rev. Lett*, vol. 99, pp. 248101–248104, 2007.

[4] J. C. López Alfonso, N. Jagiella, L. Núñez, M. A. Herrero, and D. Drasdo, "Estimating dose painting effects in radiotherapy: A mathematical model," *PLoS ONE*, vol. 9, p. e89380, 02 2014.

[5] D. Drasdo, "Coarse graining in simulated cell populations," *Adv. Complex Syst.*, vol. 8, no. 2 & 3, pp. 319–363, 2005.

[6] X. Gao, J. T. McDonald, L. Hlatky, and H. Enderling, "Acute and fractionated irradiation differentially modulate glioma stem cell division kinetics.," *Cancer Res*, vol. 73, pp. 1481–1490, Mar 2013.

[7] S. V. Cruchten and W. V. den Broeck, "Morphological and Biochemical Aspects of Apoptosis, Oncosis and Necrosis," *Anat. Histol. Embryol.*, vol. 31, pp. 214–223, 2002.

[8] W. Bursch, L. Kleine, and M. Tenniswood, "The biochemistry of cell death by apoptosis.," *Biochem Cell Biol*, vol. 68, pp. 1071–1074, Sep 1990.

[9] J. E. Wells and J. B. Russell, "The effect of growth and starvation on the lysis of the ruminal cellulolytic bacterium fibrobacter succinogenes.," *Applied and Environmental Microbiology*, vol. 62, pp. 1342–1346, 04 1996.

[10] J. L. Goergen, A. Marc, and J. M. Engasser, "Determination of cell lysis and death kinetics in continuous hybridoma cultures from the measurement of lactate dehydrogenase release," *Cytotechnology*, vol. 11, no. 3, pp. 189–195, 1993.

[11] G. Schaller and M. Meyer-Hermann, "Multicellular tumor spheroid in an off-lattice Voronoi-Delaunay cell model," *Phys. Rev. E*, vol. 71, p. 16, May 2005.

[12] Z. Rong, E. Leitao, J. Popplewell, B. Alp, and P. Vadgama, "Needle Enzyme Electrode for Lactate Measurement In Vivo," *IEEE Sensors Journal*, vol. 8, pp. 113–120, Jan. 2008.

[13] S. S. Ozturk, M. R. Riley, and B. O. Palsson, "Effects of ammonia and lactate on hybridoma growth, metabolism, and antibody production.," *Biotechnology and bioengineering*, vol. 39, pp. 418–431, Feb 1992.

[14] D. T. Gillespie, "Exact Stochastical Simulations of Coupled Chemical Reactions," *J. Phys. Chem.*, vol. 81, no. 25, pp. 2340–2361, 1977.

[15] T. Omasa, K. Higashiyama, S. Shioya, and K. Suga, "Effects of lactate concentration on hybridoma culture in lactate-controlled fed-batch operation.," *Biotechnol Bioeng*, vol. 39, pp. 556–564, Mar 1992.
